# Adjusted imbalance ratio leads to effective AI-based drug discovery against infectious disease

**DOI:** 10.1038/s41598-025-15265-5

**Published:** 2025-08-12

**Authors:** Ons Masmoudi, Afef Abdelkrim, Emna Harigua-Souiai

**Affiliations:** 1 Laboratory of Molecular Epidemiology and Experimental Pathology-LR16IPT04, Institut Pasteur de Tunis, Université de Tunis El Manar, Tunis, Tunisia; 2 Tunisia Polytechnic School, University of Carthage, Tunis, Tunisia; 3 Research Laboratory Smart Electricity & ICT, SE&ICT Lab., LR18ES44, National Engineering School of Carthage, University of Carthage, Charguia, 2035 Tunisia

**Keywords:** Computational biology and bioinformatics, Drug discovery, Mathematics and computing

## Abstract

The success of AI-based pipelines in Drug Discovery (DD) heavily depends on three components: the dataset (size, content, imbalance ratio), the encoding system and the predictive model. This study focuses on optimizing these elements to develop robust predictive models for the biological activity of chemical molecules. We trained five machine learning and six deep learning algorithms to predict the anti-pathogen activity of chemical compounds, derived from PubChem Bioassays targeting four infectious diseases. The datasets exhibited a significant imbalance towards the inactive class. To address this, we implemented a K-ratio random undersampling approach (K-RUS) to determine optimal imbalance ratios (IRs), and compared them to conventional resampling approaches. Across all simulations, a moderate IR (1:10) significantly enhanced models’ performance. Through external validation, we assessed the generalization power of the top-performing models and observed optimal balance between true positive and false positive rates with the 10-RUS configuration. Furthermore, we investigated the chemical similarity between active and inactive classes, which revealed the underlying mechanisms of misclassification. Our findings emphasized the need for optimizing imbalance ratios and leveraging chemical diversity to improve AI-driven DD against infectious diseases.

## Introduction

Over the past years, Artificial Intelligence (AI) has gained wide interest to ground itself into the field of Computer-Aided Drug Discovery and Design (CADD) due to the increasing availability of large datasets useful for training Machine Learning (ML) and Deep Learning (DL) models^1^. These advancements have enhanced the Drug Discovery (DD) process, introducing faster, more cost-effective, and automated computational techniques that are widely used and applied in various tasks, including the prediction of chemical compounds’ properties such as toxicity^2^, biological activity against specific organisms or enzymes^3^, solubility^4^, among others^5,6^. They also apply in the different stages of the CADD process, including predicting drug-target interactions^7^, optimizing therapeutic candidates^8^, and even designing novel compounds^9,10^.

Nonetheless, challenges related to data quality, quantity and availability in DD remain significant. Datasets originating from High-throughput Screening (HTS) involve testing of a large number of chemical compounds for their biological activity against a specific biological target^11^, and result in a notable majority of compounds with no activity, leading to a high number of inactive samples and a small fraction of active entities^12^. This results in highly imbalanced datasets, constituting a critical challenge for ML and DL algorithms and leading to biased predictions toward the majority class (inactive) and difficulties in effectively learning the features associated with the minority class (active)^13,14^. Various potential solutions have been used to address this issue which can be categorized into algorithm-level, data-level and hybrid-level approaches^15^.

Algorithm-level methods focused on developing new models, modifying existing algorithms to improve their ability to handle imbalanced class distributions, leveraging ensemble learning techniques, or using cost-sensitive learning approaches that assign penalties for errors or appropriate weights to the minority class. For example, Li et al. proposed the Granular Support Vector Machines Repetitive Undersampling (GSVM-RU) approach, a variant of the SVM model that selects informative inactive samples and combines them with all active samples to construct support vectors^16^. Chen et al. developed the Weighted Random Forest algorithm, which assigns weights to each class, prioritizing the minority class by assigning greater weights^17^. Additionally, Korkmaz studied the effects of various factors such as batch size, learning rate, and the minority class rate on the performance of a Deep Neural Network in classifying drug compounds. Their results revealed that the minority class weighting played a crucial role in improving the model performance, particularly in imbalanced datasets^18^. While algorithm-based approaches proved effective, they required adjustments tailored to each specific algorithm.

In contrast, data-level methods rather addressed the imbalance issue from the data perspective by using data augmentation and resampling techniques^19–21^. For image-based data, augmentation methods included rotation, flipping, cropping, and applying filters to artificially enrich datasets^22^. In cheminformatics, SMILES enumeration was used to generate multiple valid representations of a single chemical compound to increase the dataset size^19^. Resampling techniques aim to create balanced datasets through generating synthetic samples (SMOTE)^23^, randomly duplicating instances from the minority class (Random OverSampling) or undersampling the majority class, where a subset of instances is randomly removed to match the minority class size^21^. Various research applied both undersampling and oversampling techniques to address imbalanced data issues^24–26^. While undersampling can lead to potential information loss, oversampling often leads to information duplication with overfitting or bias risks. Another work, applied a variety of resampling methods, including both random oversampling and undersampling techniques, and an augmented variant (AugRandOS /AugRandomUS) which used the most common feature fingerprints to reduce information loss^27^. The authors also implemented synthetic sample generation using SMOTE variants, such as SMOTETC and clustering-based sampling methods like k-medoids to select representative samples^27^. Other groups employed undersampling techniques to create different bootstrap samples with equal class sizes in the training set to address the reduction in dataset size^28,29^. Effectiveness of undersampling versus oversampling remains a matter of discussion in the literature^27^. Our group has previously investigated an enrichment strategy through adding a few novel active instances that did not affect the Imbalance Ratio (IR), while significantly enhanced classification outcomes as compared to classical oversampling and undersampling techniques. These findings indicated that the imbalance problem is not well addressed by traditional resampling techniques while the enrichment was a successful strategy to enhance the models performances^30^. Nevertheless, the data enrichment approach involved manually collecting data from the literature through a time-consuming and labor-intensive process.

In addition to algorithm-level and data-level approaches, hybrid approaches have also been explored to leverage the strengths of both strategies. Zakharov et al. demonstrated that undersampling approaches and hybrid approaches that consist of reducing the majority class size to three times that of the minority class (1:3 ratio) and optimizing decision thresholds using leave-one-out cross-validation proved effective in improving prediction accuracy^31^.

In the present work, we focused on exploring the impact of the IRs on the performances of different classifiers in predicting the biological activity of chemical compounds. We trained five Machine Learning (ML), four graph-based and two pre-trained models on highly imbalanced bioassay datasets. We used three synthetic resampling techniques and two random resampling techniques and introduced a novel strategy, the K-Ratio Undersampling which is based on Random UnderSampling (RUS) to create three specific ratios (1:50, 1:25, and 1:10) for each imbalanced dataset. We then evaluated the impact of these ratios on the aforementioned models through F1-scores. Then, we assessed the robustness of all models through external validation on unseen datasets. Finally, we performed an in-depth investigation to assess the impact of each dataset content on models’ performances.

## Results

### Models’ performance on the original and resampled datasets

In our study, we trained five ML and six Deep Learning (DL) algorithms including four graph-based models and two pre-trained transformer-based models namely: Random Forest (RF), Multi Layer Perceptron (MLP), K-Nearest Neighbors (KNN), eXtreme Gradient Boosting (XGBoost), Naive Bayes (NB), Graph Convolution Network (GCN), Graph Attention Network (GAT), Attentive Fingerprint (AFP), Message Passing Neural Network (MPNN), ChemBERTa and MolFormer. The models were trained on four highly imbalanced PubChem bioassay datasets targeting four different infectious diseases namely the Acquired Immune Deficiency Syndrome (caused by HIV), Malaria, Human African Trypanosomiasis (*Trypanosoma brucei*) and COVID-19. The IR, defined as the ratio of the number of minority class samples (active molecules) to that of the majority class samples (inactive molecules), ranged from 1:82 to 1:104.

**Fig. 1:**
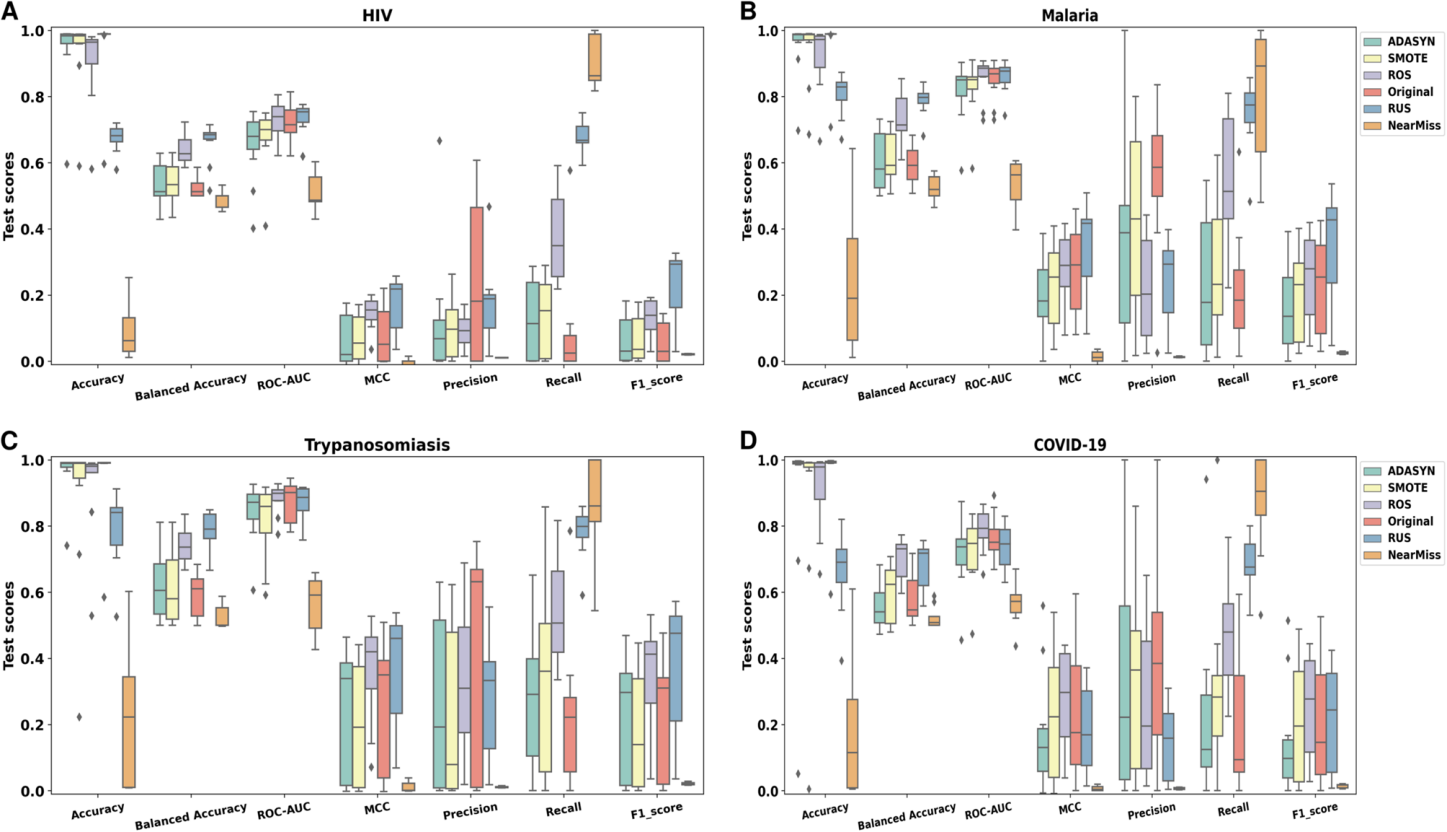
Algorithms’ performance distribution obtained with the different ML and DL models when trained on 6 data states for each dataset. (**A**) A boxplot displaying the performance of all models based on the considered version of the HIV dataset. (**B**) A boxplot displaying the performance of all models based on the considered version of the Malaria dataset. (**C**) A boxplot displaying the performance of all models based on the considered version of the Trypanosomiasis dataset. (**D**) A boxplot displaying the performance of all models based on the considered version of the COVID-19 dataset.

Due to the significant imbalance favoring the inactive class, we evaluated the effect of classical data balancing techniques by increasing the number of minority instances or reducing the number of majority instances to improve model performance. We used ADASYN, SMOTE, ROS, NearMiss and RUS techniques to achieve a balanced ratio (1:1) between the active and inactive classes in each dataset. Then, we assessed the models’ performances across the different settings (Fig. 1).

The classification performance on the original HIV dataset (IR 1:90) was very poor, with MCC values lower than 0 ($-0.04$) for all models (Fig. 1A). Random oversampling (ROS) significantly boosted recall, slightly increased the ROC-AUC, balanced accuracy and F1-scores but significantly decreased precision. Random undersampling (RUS) outperformed other techniques, enhancing the ROC-AUC, balanced accuracy, MCC, Recall and F1-score. Across all the simulations, the maximum distinction power between the inactive and active class was observed with models trained on the undersampled dataset. Overall, synthetic resampling methods showed limited improvements with the NearMiss technique achieving the highest recall. The Malaria dataset presented the lowest IR of 1:82. Models’ performances on the imbalanced dataset were better than those observed with the HIV dataset across all metrics (Fig. 1B). ROS and RUS enhanced the balanced accuracy and recall scores, with a significant decrease in precision. RUS yielded the best MCC values and F1-score. However, the models trained on datasets oversampled with synthetic techniques (ADASYN & SMOTE) performed similarly to the models trained on the original data in terms of balanced accuracy, MCC, precision, recall and F1-score. The ADASYN technique showed the highest Precision. Conversely, the NearMiss method achieved the highest recall but low performances otherwise. Overall, all data states showed consistent performances in terms of the ROC-AUC. For the Trypanosomiasis dataset, the models trained on the imbalanced dataset (original) presented the worst performances in terms of balanced accuracy, MCC, Recall and F1-score compared to ROS and RUS techniques (Fig. 1C). Nonetheless, the best scores were reached with the RUS data state. The COVID-19 dataset was the most imbalanced dataset (IR 1:104). All resampling methods failed to enhance the performance of the models trained on this dataset across all metrics except for balanced accuracy and recall (Fig. 1D). We observed a significant improvement in recall with ROS, RUS and NearMiss datasets with an advantage for undersampling methods. When comparing resampling techniques, SMOTE led to the highest MCC and F1-score. However, ADASYN demonstrated the highest Precision and ROS exhibited the highest balanced accuracy scores. This dataset presented different findings compared to the previous ones (HIV, Malaria and Trypanosomiasis) probably due to its extreme imbalance state (only 1093 active compounds versus 114,778 inactive ones).

Across all four datasets, accuracy values appeared consistently high for oversampling and original data states.

In contrast, NearMiss and RUS induced low accuracy values, with NearMiss often yielding the lowest scores. Nonetheless, the high accuracy values can be misleading in the context of imbalanced datasets, as it primarily reflects correct predictions of the majority class. Our results showed that for highly imbalanced datasets (IR: 1:82–1:104), RUS outperformed ROS based on several metrics. This suggested that reducing the number of the majority class instances through undersampling, although underlying information loss, derived better performances. It is important to highlight that duplicating instances can contribute to overfitting on active compounds. Overall, the results did not reach the desired level of performance.

### Impact of imbalance ratio on models’ performances

#### From the models’ perspective

To better understand the effects of the IR on models’ performances, we first included two additional undersampling methods namely One-Side Selection (OSS) and Neighborhood Cleaning Rule (NCR). Unlike resampling methods designed to balance the dataset, OSS and NCR focus on refining data quality. Both methods focus on removing noisy, ambiguous or borderline examples from the majority class, thus reducing artifacts that could hinder model learning. Depending on the dataset, these methods may moderately affect the IR. Additionally, we investigated the impact of different ratio values. We applied a K-RUS strategy to the inactive class to obtain various class size ratios, namely 1:50, 1:25, and 1:10 alongside the previously investigated 1:1 ratio using ADASYN, SMOTE, ROS, RUS, and NearMiss. The K-RUS strategy relied on empirically evaluating multiple values of $K \in \{50, 25, 10\}$ to identify a consensual K value that yields optimal performance across benchmark datasets. The rationale is to preserve a significant subset of majority class diversity while preventing the learning algorithm from becoming biased toward negative samples. We built on standard RUS, which reduces majority samples as follows:

$$\begin{aligned} N_{\text {maj\_new}} = k \times N_{\text {min}} \end{aligned}$$

where $k = 1$, $N_{\text {min}}$ is the number of minority class samples, and $N_{\text {maj}}$ is the original number of majority class samples. The K-RUS strategy reduces the majority class according to the same formula, with $k$ corresponding to the desired target ratio.

**Fig. 2:**
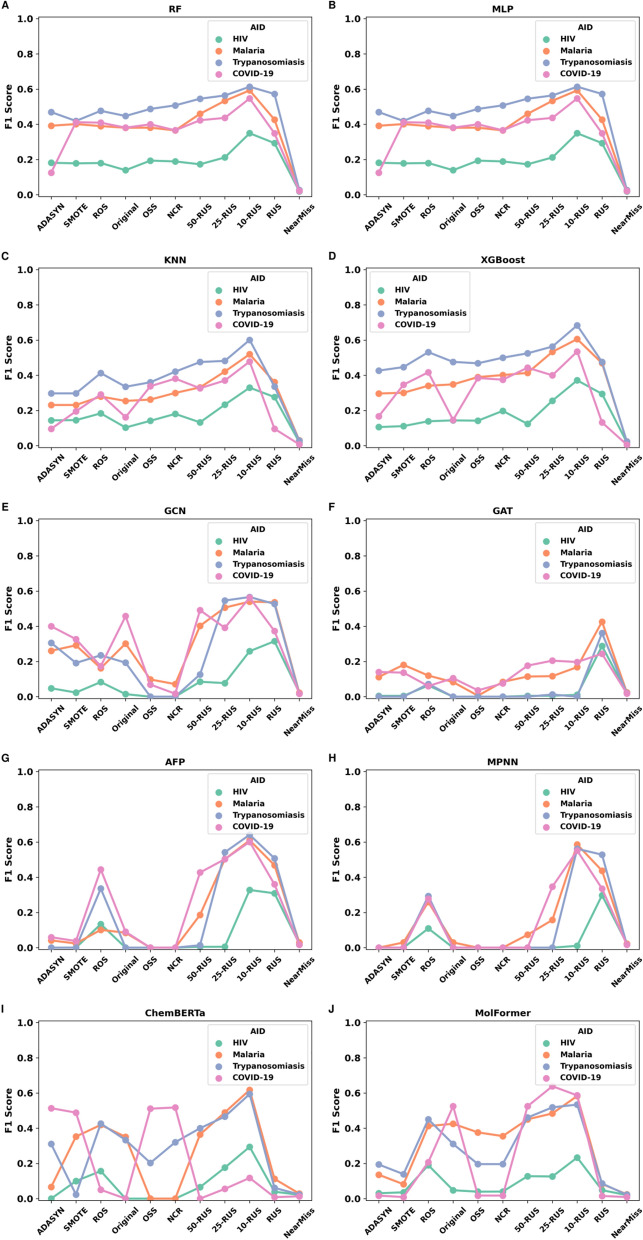
F1-score comparison of ten algorithms (RF, MLP, KNN, XGBoost, GCN, GAT, AFP, MPNN, ChemBERTa, and MolFormer) across the different data states when trained on the different datasets (HIV, Malaria, Trypanosomiasis, and COVID-19).

From the previous findings, we consistently observed a trade-off between precision and recall which resulted in significant variations in the F1-score. Thus, we opted for the F1-score as the leading metric to assess the performance of each model across the various dataset ratios. We analyzed the behavior of each model across the different sampling approaches (Fig. 2). The RF model showed a progressive performance until reaching the maximum F1-score with the 1:10 ratio and the RUS resampling technique (Fig. 2A). This growing curve was observed first with the OSS and NCR methods and continued through the K-RUS variants. For the HIV and Trypanosomiasis dataset, RUS slightly outperformed 1:10. However, the 1:10 ratio led to the best scores for Malaria and COVID-19. The MLP model demonstrated more stable enhancement as the IR decreased from Original to 1:10 (Fig. 2B). It achieved its best results with 1:10 ratio for all datasets. Both KNN and XGBoost models showed similar performance trends across the various data states and datasets, with optimal results observed under the 10-RUS settings (Fig. 2C,D). The RF, MLP, KNN, and XGBoost models exhibited low performances and displayed a distinct trend when trained on the COVID-19 dataset compared to other datasets under the ADASYN configuration. The NB model showed very low performances across all resampling methods, including OSS and NCR. It showed relative improvement at 25-RUS that reached its maximum at 10-RUS, which is below 0.4 (Supplementary Figure S1). GCN reached its F1-score peak at 1:10, except for HIV where it was achieved with RUS (1:1) (Fig. 2E). The 1:25 IR showed comparable performance to 1:10 for both Malaria and Trypanosomiasis datasets. The oversampling techniques (ADASYN, SMOTE and ROS) were less effective for GCN. With GAT, we noticed very low performances across resampling strategies except for RUS, where it showed a notable improvement for all datasets (Fig. 2F). The AFP and MPNN models started showing good performance at 1:50 and achieved the maximal F1-score at 1:10 IR (Fig. 2G,H). For instance, with AFP, the HIV dataset exhibited non-zero F1-scores only under the ROS, 1:10, and RUS resampling strategies. In contrast, with MPNN, this dataset demonstrated non-zero F1-scores only with ROS and RUS. The OSS and NCR strategies proved ineffective for the graph-based models, resulting in very low F1-scores (near zero), performing worse than the original dataset.

The performance of ChemBERTa was slightly improved across the variations of the IR, hitting a maximum at 1:10 ratio with moderate performance for Trypanosomiasis and Malaria (around 0.6), but performing poorly for COVID-19 and HIV. The COVID-19 led to the highest performance of this model with OSS and NCR (Fig. 2I). MolFormer achieved its best results between 1:25 and 1:10 ratios (Fig. 2J). It demonstrated more stable performances across different strategies compared to ChemBERTa, indicating greater robustness. Under the OSS and NCR methods, MolFormer’s performance remained stable or declined.

Classification performance for all models generally improved as the IR decreased from  1:100 (original) to 1:10. Oversampling techniques (ROS and SMOTE) were the least effective. NearMiss consistently produced the poorest results across all models and datasets. The 10-RUS emerged as the best configuration as it yielded to the highest F1-score for most models, except for GAT. F1-scores of all simulation results were listed in Supplementary Table S1.

#### From the datasets’ perspective

**Fig. 3:**
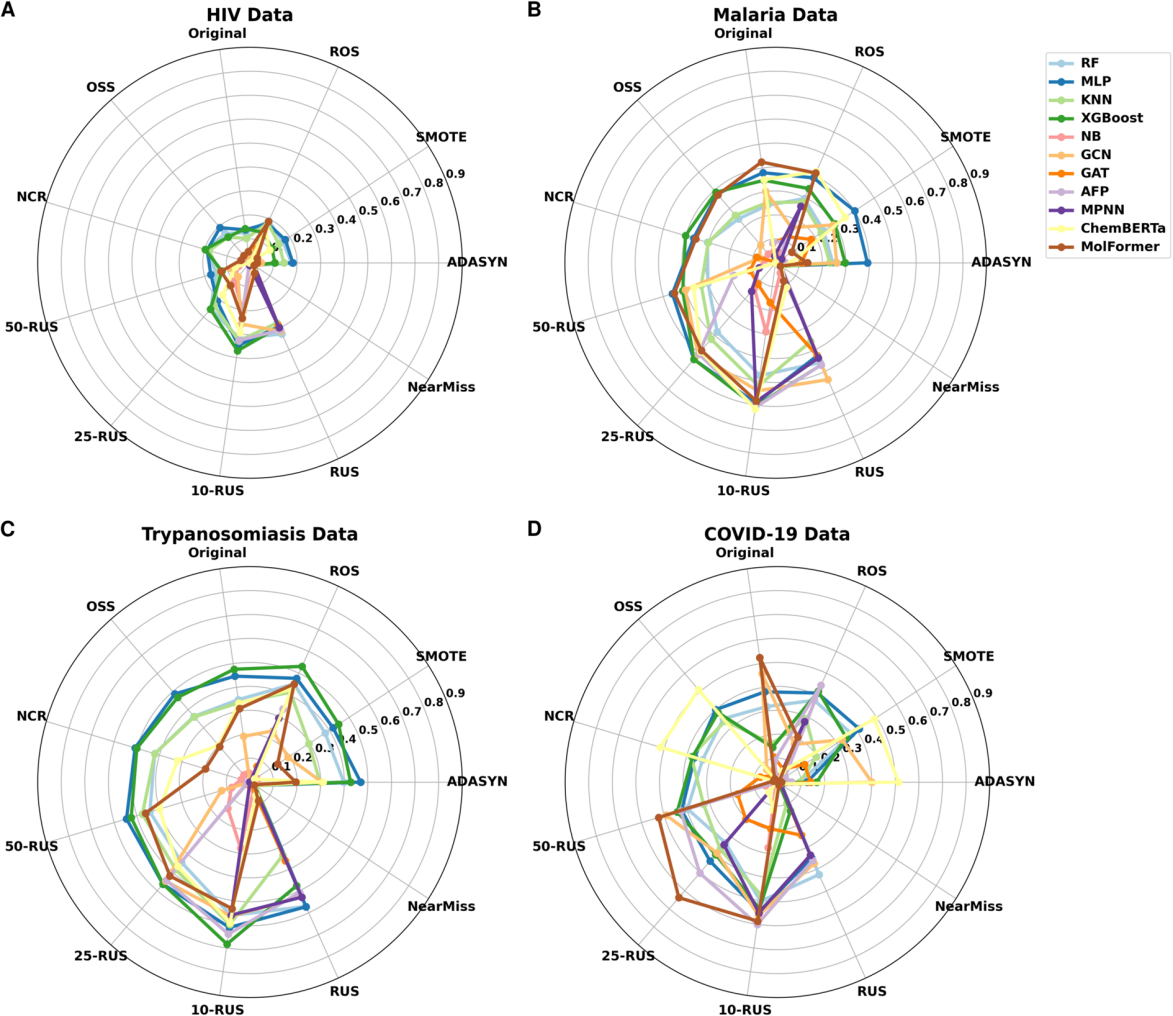
Radar plots: visualizing F1-scores for models trained on different datasets at different configurations. (**A**) Models’ performance on the HIV dataset. (**B**) Models’ performance on the Malaria dataset. (**C**) Models’ performance on the Trypanosomiasis dataset. (**D**) Models’ performance on the COVID-19 dataset.

Through radar plots, we analyzed the same results from the dataset perspective, providing a visual summary of models’ F1-scores for each dataset across the different states (Fig. 3). When a point is positioned farther from the middle, it indicates better performance.

For the HIV dataset, we noticed that the F1-scores were very low across all models. Nevertheless, MLP, RF, KNN and XGBoost consistently performed well across different dataset versions. At 50-RUS , only MolFormer showed moderate improvement but the other models remained close to zero. With the subsequent ratio, ChemBERTa emerged as one of the best predictors for this dataset. At 1:10, AFP followed by GCN and NB moved to a significant peak. Overall, The HIV dataset presented unsatisfactory classification performance across all eleven models and the eleven data versions. It had a maximum F1-score value of 0.37 (Fig. 3A). Under high IRs (between 1:90 and 1:50) of the Malaria dataset, MolFormer, MLP and XGBoost showed high F1-score peaks demonstrating an ability to navigate class imbalance. GCN and ChemBERTa performed well under Original and 50-RUS conditions, but their performance dropped significantly when trained on OSS and NCR sampling methods (Fig. 3B). The performance of all models was improved progressively as the IR decreased, reaching their peak performance at the 1:10 ratio. The AFP model showed minimal improvement at 1:50 that further increased at 1:25 and 1:10. For Trypanosomiasis dataset, MLP, XGBoost, RF, KNN, ChemBERTa and MolFormer demonstrated robust performances across all resampling methods, even under high IRs (Fig. 3C). GCN and AFP achieved optimal F1-scores at 25-RUS. Performances of all models progressively improved and reached their best when the IR hit 1:10. The MPNN model displayed scores close to the center at high IRs. Then, its performance became more noticeable at 1:10 ratio. For COVID-19, MolFormer and GCN showed the best performances for original and 1:50 IR versions, followed by ML models. As the IR decreased, AFP and MPNN appeared as top-performing models (Fig. 3D). However, ChemBERTa demonstrated the worst F1-score values for this dataset across the different data states except for OSS and NCR methods where it showed the highest values and outperformed all models.

When moving from the high to less severe IRs 1:25 and particularly 1:10 most models presented significant improvement, except NB and GAT which showed the lowest F1-score overall, underscoring their limitations in the context of molecule activity classification (Fig. 3).

Our results demonstrated that the 1:10 IR correlated with the optimal performances across all datasets and models. This suggested that a moderate class imbalance achieved through data reduction (K-RUS) is preferable to under or oversampling (1:1). Based on this ratio, we identified the top performing models for each data. For the HIV dataset, three ML models namely XGBoost, MLP and KNN achieved the best performances followed by AFP (Fig. 3A). For the Malaria dataset, ChemBERTa, AFP, XGBoost and MLP achieved the highest F1-scores (Fig. 3B). The Trypanosomiasis dataset showed similar performance across all models, with a slight advantage for XGBoost, followed by AFP, MLP and KNN (Fig. 3C). For COVID-19, AFP, MolFormer, GCN and MPNN emerged as the top-performing models (Fig. 3D).

These findings highlighted the need to tailor model selection and data balancing to the unique characteristics and specific challenges of each dataset. In fact, it is noteworthy to mention that the impact of class imbalance varied from one dataset to another, indicating that there is no one-size-fits-all solution to address this issue. The HIV dataset consistently presented lower F1-scores than the other datasets, demonstrating that the performance of the models are also influenced by the chemical content of the dataset.

#### Model optimization

The selected top-performing models for each dataset went through a grid search to select the optimal hyper-parameters specific to each dataset that maximize the F1-score and balanced accuracy. Additionally, we conducted a 5-fold cross-validation. Each dataset was considered separately during the hyper-parameters tuning process. This approach allowed us to extract for each model its parameters to the specific characteristics of the dataset. The detailed results of hyper-parameters tuning and cross-validation process are reported in the Supplementary Table S2.

The hyperparameter tuning led to notable shifts in model performance across different datasets through improvements in F1-scores for many models (Table 1). For instance, for the HIV dataset, the F1-score of the KNN, and AFP models were improved by $+0.03$. Similarly, on the COVID-19 dataset, the MolFormer model showed a slight improvement, with its F1-score rising from 0.58 to 0.60, and the GCN model improved from 0.56 to 0.57.

**Table 1:** Optimized model performances obtained through 5-fold cross-validation, reported as mean ± confidence interval (CI).

Dataset	Balanced accuracy	MCC	Precision	Recall	F1-score	Cohen’s Kappa
XGBoost_HIV	0.63 ± 8.5E−5	0.31 ± 1.2E−4	0.47 ± 2.2E−4	0.30 ± 6.6E−5	0.37 ± 1.0E−4	0.30 ± 1.4E−4
KNN_HIV	0.64 ± 7.5E−5	0.34 ± 8.53E−5	0.48 ± 1.4E−4	0.30 ± 1.0E−4	0.37 ± 1.0E−4	0.33 ± 7.5E−5
MLP_HIV	0.64 ± 1.4E−4	0.31 ± 1.7E−4	0.41 ± 2.3E−4	0.37 ± 3.6E−4	0.37 ± 2.1E−4	0.31 ± 2.3E−4
AFP_HIV	0.64 ± 1.0E−4	0.31 ± 9.4E−5	0.41 ± 1.8E−4	0.32 ± 2.7E−4	0.36 ± 1.2E−4	0.31 ± 1.0E−4
ChemBERTa_Malaria	0.86 ± 6.5E−4	0.53 ± 3.0E−4	0.68 ± 2.9E−4	0.47 ± 5.9E−4	0.55 ± 3.9E−4	0.5 ± 4.2E−4
AFP_Malaria	0.78 ± 7.9E−5	0.58 ± 9.9E−5	0.65 ± 1.0E−4	0.58 ± 3.0E−4	0.62 ± 9.9E−5	0.57 ± 2.1E−4
XGBoost_Malaria	0.72 ± 9.9E−5	0.57 ± 1.1E−4	0.8 ± 1.6E−4	0.47 ± 1.1E−4	0.59 ± 1.3E−4	0.53 ± 2.0E−4
MLP_Malaria	0.75 ± 9.9E−5	0.55 ± 9.9E−5	0.66 ± 3.0E−4	0.52 ± 3.0E−4	0.58 ± 9.9E−5	0.54 ± 2.1E−4
XGBoost_Trypano	0.75 ± 1.2E−4	0.62 ± 2.3E−4	0.83 ± 7.4E−4	0.50 ± 2.3E−4	0.63 ± 2.0E−4	0.59 ± 3.0E−4
AFP_Trypano	0.78 ± 1.2E−4	0.58 ± 1.5E−4	0.65 ± 1.5E−4	0.58 ± 4.6E−4	0.62 ± 1.5E−4	0.54 ± 4.6E−4
MLP_Trypano	0.76 ± 3.2E−4	0.56 ± 3.0E−4	0.66 ± 7.7E−4	0.53 ± 4.6E−4	0.59 ± 2.4E−4	0.55 ± 3.0E−4
KNN_Trypano	0.75 ± 1.0E−4	0.56 ± 2.0E−4	0.67 ± 6.3E−4	0.52 ± 2.4E−4	0.59 ± 1.7E−4	0.55 ± 2.0E−4
AFP_COVID	0.72 ± 2.3E−4	0.51 ± 5.8E−4	0.62 ± 7.6E−4	0.48 ± 3.5E−4	0.54 ± 4.1E−4	0.48 ± 7.1E−4
MolFormer_COVID	0.91 ± 1.7E−4	0.57 ± 5.0E−4	0.73 ± 5.0E−4	0.51 ± 8.0E−4	0.60 ± 5.0E−4	0.59 ± 7.0E−4
GCN_COVID	0.78 ± 8.0E−4	0.55 ± 1.0E−4	0.59 ± 3.5E−4	0.61 ± 2.5E−4	0.57 ± 8.0E−4	0.44 ± 1.4E−4
MPNN_COVID	0.66 ± 0.001	0.54 ± 0.001	0.79 ± 1.8E−3	0.4 ± 5.0E−4	0.52 ± 6.0E−4	0.40 ± 6.0E−4

However, for this dataset, there were instances where performance slightly declined. For example, ChemBERTa on the Malaria dataset, showed a decrease, from 0.61 to 0.55. Additionally, the AFP and MPNN models on the COVID-19 dataset declined from 0.60 to 0.54 and from 0.55 to 0.52, respectively. For the Trypanosomiasis dataset, all models declined by approximately 0.02. This decrease was likely due to variations in data characteristics across the different folds during cross-validation, which can lead to suboptimal performances in the different folds. This highlights the importance of dataset-specific tuning, as the optimal hyperparameters can vary significantly depending on the dataset’s characteristics.

**Fig. 4:**
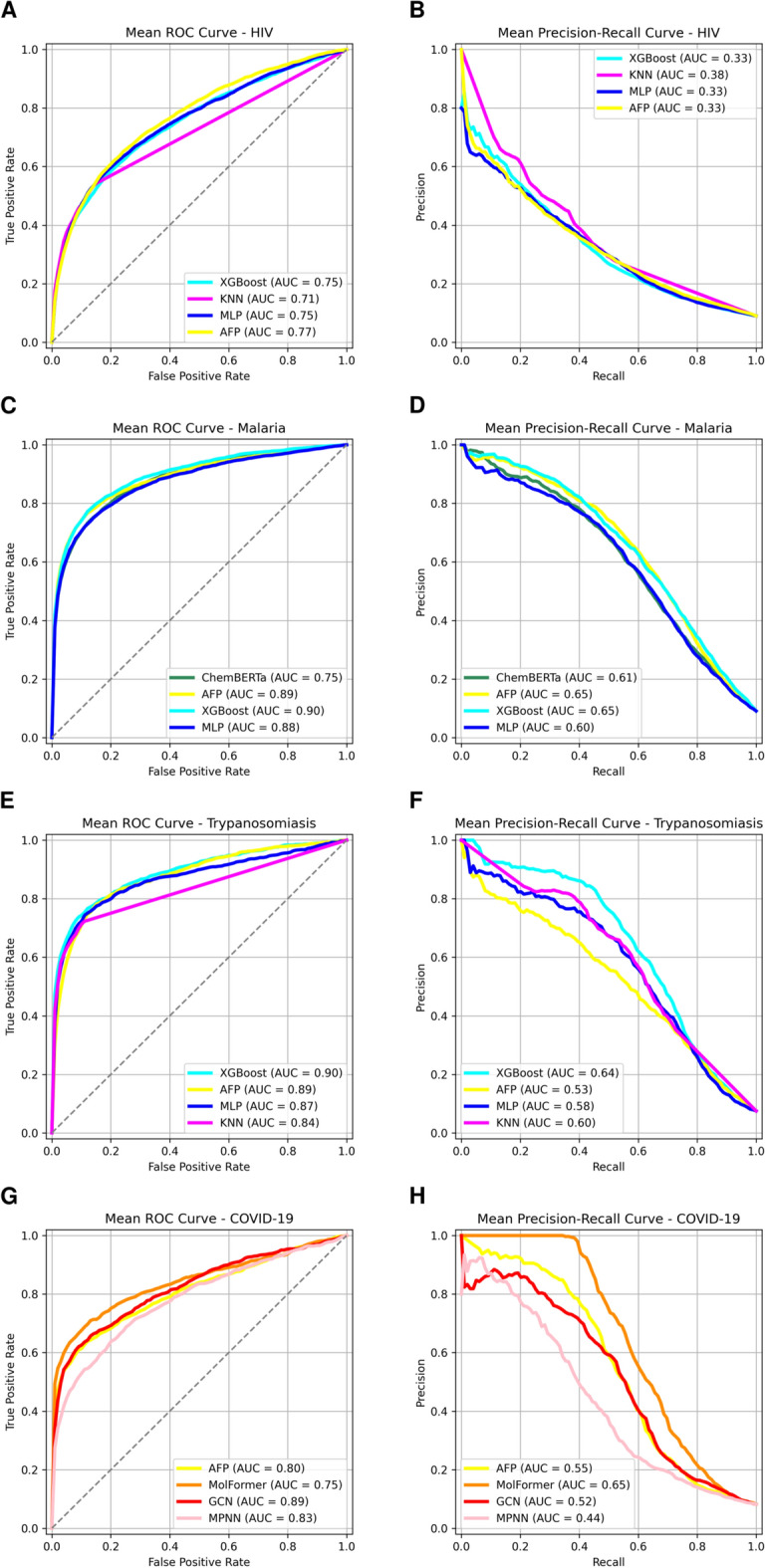
ROC and PR curves of the optimized models on the 10-RUS dataset. (**A**) ROC curves for models trained on the HIV dataset. (**B**) PR curves for models trained on the HIV dataset. (**C**) ROC curves for models trained on the Malaria dataset. (**D**) PR curves for models trained on the Malaria dataset. (**E**) ROC curves for models trained on the Trypanosomiasis dataset. (**F**) PR curves for models trained on the Trypanosomiasis dataset. (**G**) ROC curves for models trained on the COVID-19 dataset. (**H**) PR curves for models trained on the COVID-19 dataset. *AUC* area under the curve, *ROC* receiver operating characteristic.

The ROC and Precision-Recall (PR) curves illustrating the performance variability of the models across datasets were generated (Fig. 4). For the HIV dataset, the ROC and PR curves of all models showed progressive slope, suggesting moderate class separability. Noticeably, the shape of the PR curve revealed poor ability to predict active molecules while keeping the false positive rate low. In contrast, for Malaria and Trypanosomiasis datasets, all models showed comparable performances in terms of ROC and PR curves. Noticeably, the ROC curves demonstrated strong overall discriminative performance, with AUCs approaching or exceeding 0.88 for most models. The PR curves supported these trends with consistent high precision across a wide range of recall values. For the COVID-19 dataset, MolFormer demonstrated the best PR curve profile, with acceptable AUC values for PR and ROC curves across all models (Fig. 4). The best performing models herein optimized are accessible for direct use by the DD community through our GitHub repository https://github.com/Ons23/Optimized_Models.

### External validation

For each dataset, we performed an external validation to assess the ability of the optimized models to predict potential inhibitors against each disease in real-world applications. We used 4 confirmatory bioassays that are experimentally related to the original datasets used up to this point (Table 2). We removed duplicate molecules shared between the training dataset and the external validation set. We retrained the top-performing models using the optimized hyperparameters on the original, RUS and 10-RUS versions of each dataset. Then, we used these models to predict the activity class of each molecule in the external validation set. We evaluated the classification outcomes through the confusion matrix (TP, TN, FP and FN) for each model under two different scenarios (Supplementary Table S3). In the first scenario, we calculated the elements of the confusion matrix without considering the prediction probability. In the second scenario, we selected the molecules classified with a confidence level of 80% or higher by all models. This served to assess how effective our classifiers would be in identifying and top-listing promising drug candidates.

**Table 2:** Characteristics of confirmatory bioassays.

Confirmatory AID	Target	Active	Inactive
AID 687023	HIV	44	11
AID 588678	Malaria	23	40
AID 651971	Trypanosomiasis	137	112
AID 1890	COVID-19	44	57

Taking Malaria as a case study, for each combination of model-data-configuration, we looked at the TP counts out of the 23 active molecules. The AFP model performed the best with 22 out of 23 as TP when trained on the 10-RUS and RUS settings. Even when considering the 80% confidence threshold, this model maintained the same number of predicted active molecules. GCN identified 22 out of 23 as TP, while the XGBoost and RF models showed less satisfying results, with the lowest TP count (21 and 19 out of 23, respectively) and the highest number of FP (22 and 24 out of 40 inactive molecules, respectively) when trained on the RUS dataset. The models trained on the original dataset showed sub-optimal predictions with MolFormer performing the least correct predictions. It only correctly predicted 9 active out of 23 and 15 inactive out of 40. MLP, ChemBERTa and XGBoost correctly predicted 12, 16 and 12 active molecules, respectively. In contrast, they correctly predicted the inactive molecules with 38 out of 40 as TN. When trained on 1:10 ratio, these models showed an enhancement in their predictions. MLP was able to deliver 18 out of 23 as TP with 16 presenting a probability higher than 0.80. ChemBERTa identified 21 active molecules within the selection of the confidence threshold 80%. However, XGBoost was able to predict only 14 TP with 8 instances presenting probability above the confidence threshold. The results obtained for all datasets are reported in Supplementary Table S3.

**Fig. 5:**
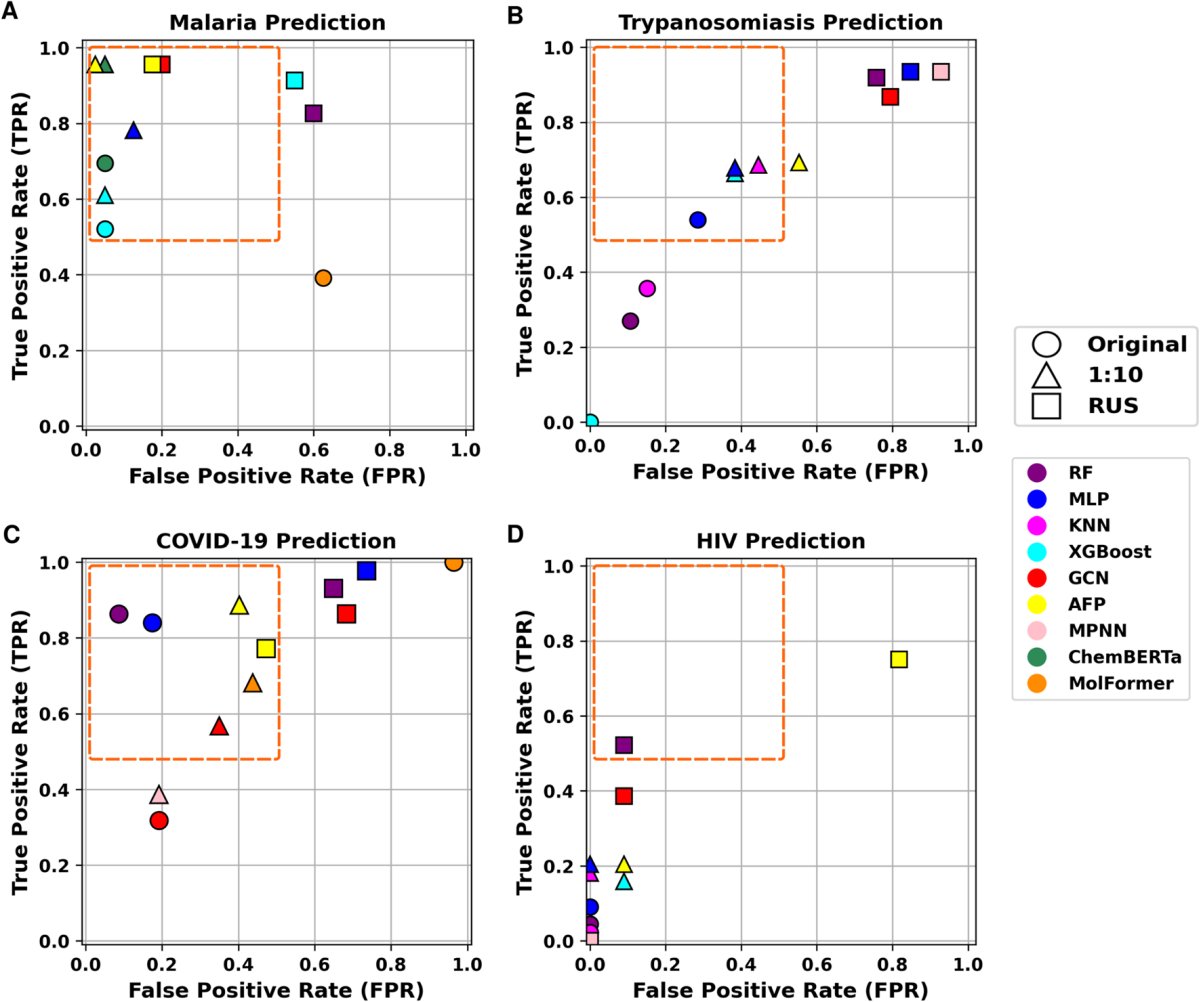
Trade-off between TPR and FPR through 3 data states. (**A**) Optimized models prediction performance for Malaria. (**B**) Optimized models prediction performance for Trypanosomiasis. (**C**) Optimized models prediction performance for COVID-19. (**D**) Optimized models prediction performance for HIV.

For all datasets, we plot the True Positive Rate (TPR) versus False Positive Rate (FPR) of each model in order to assess the predictive performance (Fig. 5). Ideally, a model should achieve a high TPR while maintaining a low FPR (targeted zone: red rectangle on Fig. 5). Across all datasets, the models trained on the 10-RUS configuration (triangle markers), consistently showed results within the targeted zone, indicating a good generalization power overall. These findings confirmed that 10-RUS is the most effective strategy to build classifiers that exhibit an optimal balance between TPR and FPR. Nevertheless, each model showed varying levels of success in balancing TPR and FPR across the different datasets, indicating that no single model has a distinct advantage and that model’s selection should be tailored to each specific dataset and the classification objectives.

For instance, the Malaria dataset induced the best performance among the four diseases, with all models under the different data states achieving a satisfactory balance between TPR and FPR. Conversely, The HIV dataset appeared to be highly challenging to all models, leading to poor performances overall. For the COVID-19 and Trypanosomiasis datasets, model performance was suboptimal, and most models failed to achieve a consistent balance between TPR and FPR mainly with the original imbalance and the RUS (1:1) datasets.

### Analysis of misclassifications using structural similarity

Through previous findings, the HIV dataset proved to be challenging compared to the other datasets. To better understand the underlying reasons for the models’ misclassifications, we established Chemical Space Networks (CSNs) as a visualization tool of the relationships (chemical similarity) between compounds in both the 10-RUS training dataset (primary bioassay) and the external validation dataset (confirmatory bioassay). To quantify these molecular similarities, we used the Tanimoto coefficient as a similarity metric, and set a threshold of 0.7 to define structurally similar molecules that shall be connected in the networks.

**Fig. 6:**
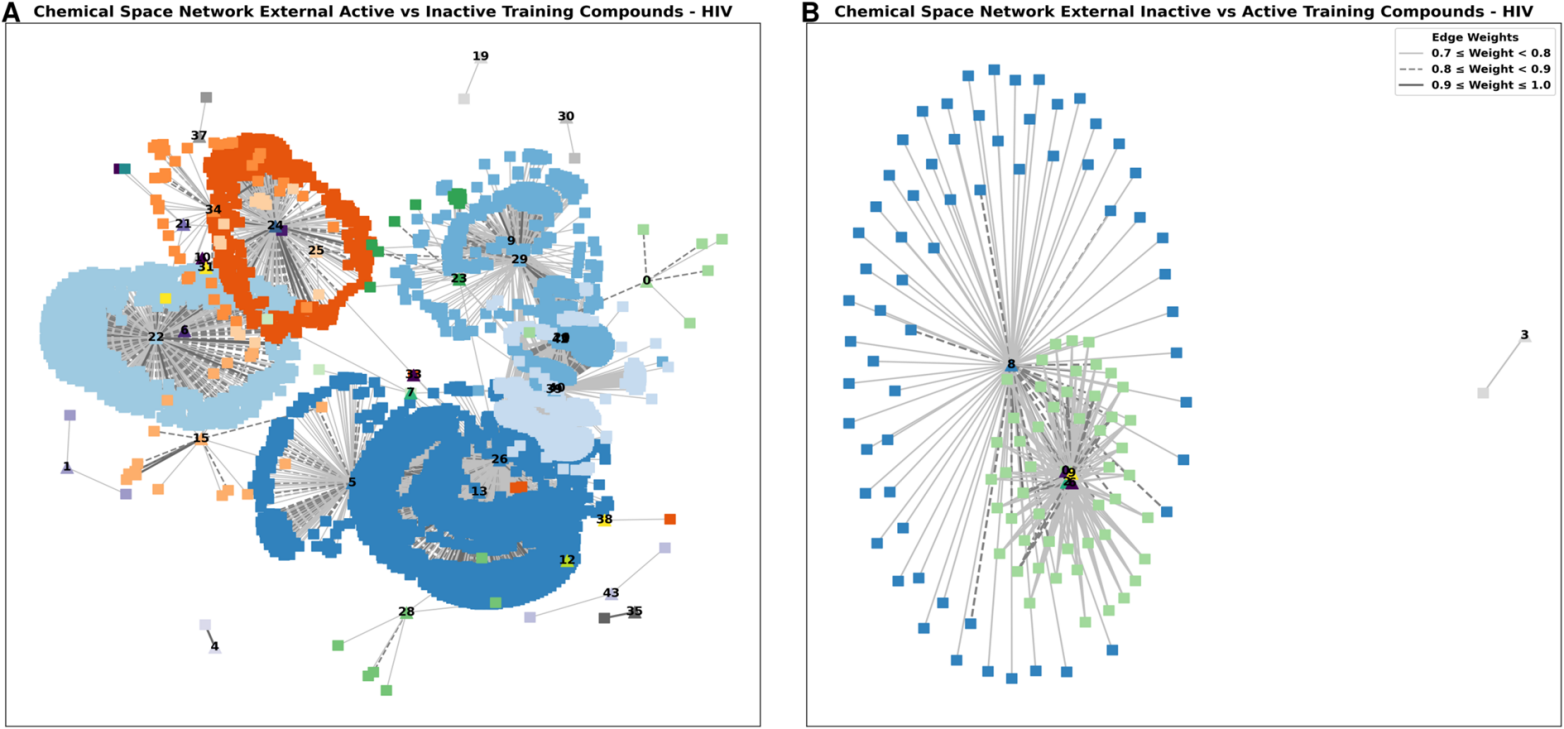
Chemical Space Network of the compounds in the external dataset and the training set of HIV. (**A**) Chemical Space Network of the active compounds in the external dataset (triangle) and the inactive compounds in the training set (square). (**B**) Chemical Space Network of the inactive compounds in the external dataset (triangle) and the active compounds in the training set (square).

The relationships between active compounds in the external dataset and the inactive compounds in the training set of the HIV revealed a densely interconnected CSN, showing distinct clusters of structurally similar molecules (Fig. 6A). This indicated significant structural similarities between active and inactive molecules contributing to the high rate of FNs obtained in the external validation experiment. In fact, this made it difficult for the models to differentiate between inactive and active compounds against HIV. Among the 41 compounds incorrectly predicted as inactive, 32 exhibited a Tanimoto similarity greater than 0.7 with inactive compounds in the training set. When the similarity threshold was raised to 0.8 and 0.9, we respectively identified 20 out of 22 and 15 out of 17 compounds incorrectly predicted by the models. On the other hand, the CSNs between inactive external compounds and active training compounds (Fig. 6B) revealed less similar entities which explained the prediction of only two molecules as FP with one of them presenting a chemical similarity above 0.7 with active molecules.

**Fig. 7:**
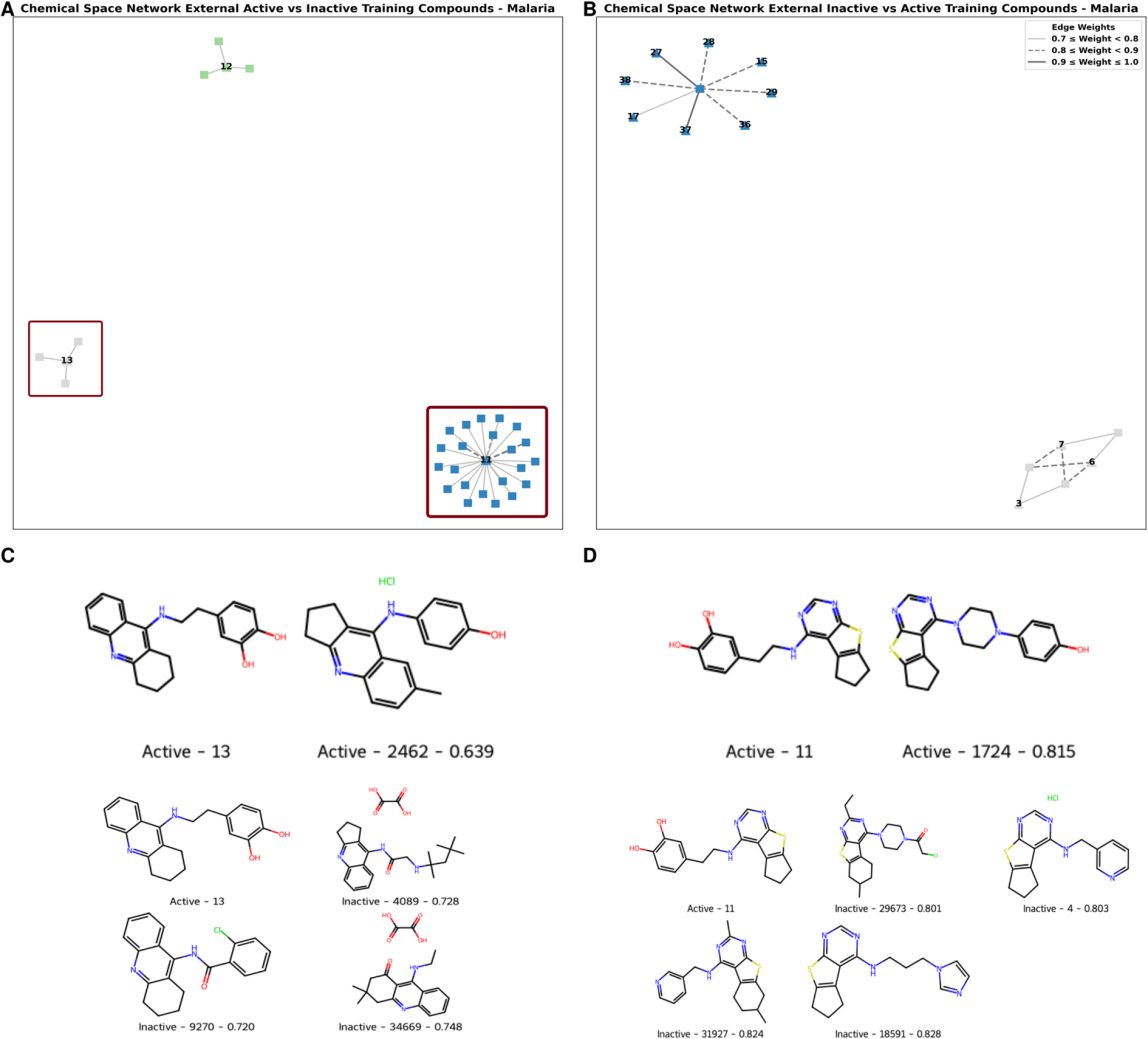
Chemical Space Network of the compounds in the external dataset and the training set of Malaria. (**A**) Chemical Space Network of the active compounds in the external dataset (triangle) and the inactive compounds in the training set (square). (**B**) Chemical Space Network of the inactive compounds in the external dataset (triangle) and the active compounds in the training set (square). (**C**) Visualization of misclassified compound N$^{\circ }$13 alongside its most similar compounds. (**D**) Visualization of misclassified compound N$^{\circ }$11 alongside its most similar compounds.

The CSNs representing the diversity between active and inactive compounds in the Malaria dataset revealed distinct patterns and a highly diverse chemical space (Fig. 7). Unlike in the HIV dataset, the Malaria compounds exhibited better structural separation between active and inactive classes, as reflected by the limited number of connections between nodes. This structural diversity contributed to more accurate predictions as observed in the external validation section. When exploring the chemical space between active compounds in the external validation set and inactive compounds in the training dataset, we observed three active nodes (represented as triangles) in the network (Fig. 7A). Only two of them (molecules N$^{\circ }$13 and N$^{\circ }$11) were identified as FN. A similar network aspect was observed with inactive external and active training molecules (Fig. 7B). Among the 11 molecules with a similarity score higher than 0.7, three were incorrectly classified. This indicated a separation in the chemical space suggesting that the structural characteristics of anti-Malaria compounds were more discriminant between the active and inactive classes, which contributed to more reliable predictive models than in the case of the HIV dataset. The CSNs representing the diversity between active and inactive compounds for the Trypanosomiasis and COVID-19 datasets are provided in the Supplementary Figures S2 and S3.

To further explore the misclassification patterns, we performed a structural and chemical similarity of misclassified compounds N$^{\circ }$ 13 and 11 (Fig. 7C,D). These molecules demonstrated a clear activity cliff. Although they shared structural similarity higher than 0.72 and 0.80, respectively, to several inactive compounds in the training set, they were biologically active. Such patterns highlighted the inevitable cases of outlier molecules for which activity profile challenges the overall distribution of the training set, and thus leading to misclassifications. This further supported the importance of considering chemical diversity of the target dataset when developing a ML model, as it impacts its ability to distinguish between active and inactive molecules.

## Discussion

AI has revolutionized the DD field, transforming it into a data-driven approach with high promise to advance research and healthcare. The success of AI-based approaches heavily relies on two main components: (1) the quality and quantity of the data, and (2) the combination of the encoding system used to transform molecular information into machine-readable format and the model which uses these representations to learn and make accurate predictions. A major challenge to AI approaches in the DD field is the data where it is either lacking or insufficient. Depending on the target disease and the experimental settings, most datasets would also present a prominent imbalance towards inactive molecules.

In fact, class imbalance is a significant challenge in developing robust predictive models. While oversampling has been widely explored and proven effective across various fields^32^, its application in theoretical chemistry mainly consisted of duplicating information^27^. Either, through ROS, SMILES enumeration or synthetic oversampling, no chemical information was added to the learning process. This was due to the fact that the encoding systems, while generating different numerical or binary vectors, will eventually code for the same chemical structure. Such approaches often failed to reflect the diversity of the chemical space, resulting in unreliable performance and poor generalization power^33^. In the specific context of chemical data resampling, several studies demonstrated that undersampling was more effective than oversampling to improve model performances^24,31,34^. Undersampling consisted in reducing instances from the majority class and consequently decreasing the overall dataset size. Although this induced information loss, it prevented overfitting by training models on limited features learned on duplicated instances of the minority class while the majority class continued to dominate the chemical feature space, as was the case with oversampling^13^. Additionally, with heavily imbalanced datasets, models failed to correctly predict the minority class^35^. In the present research, we further confirmed these hypotheses by comparing four undersampling and three oversampling techniques. Additionally, we demonstrated that moderately undersampling, which consists of reducing the majority class to a limited ratio 1:K (1:10 and 1:25), led to better results than classical undersampling (1:1). In fact, the K-ratio undersampling (K-RUS) method reduced training time and demonstrated promising results in mitigating class imbalance in AI-driven DD. Nevertheless, several limitations must be considered. First, K-RUS relied on an empirical selection of the K-ratio, which may not correspond to the optimal value across any datasets. Its effectiveness can vary depending on the data quality. Challenging quality datasets (like HIV) can hinder the benefits of the method. Additionally, the undersampling process inherently removes a portion of the majority class data, which may lead to a loss of potentially informative instances. Finally, although moderate imbalance appears beneficial in our experiments, the observed performance gain might be task-specific and not uniformly transposable to all machine learning architectures or domains.

Data imbalance and/or size are not the only characteristics that affect the predictive power of ML models. The encoding systems that translate the chemical information into numerical or binary vectors, and thus extract the subset of features that will be learned by ML models, also inevitably impact the prediction outcomes. Classically, ML models are trained on molecular FPs or descriptors, representing chemical structures as binary or numerical vectors. More recently, graph-based models were introduced in DD tasks^2,6,36–38^. They encode molecules as undirected graphs, where atoms are nodes, and bonds are edges^39^, enabling the model to capture the topological features of molecular structure. In several research studies, the application of GNNs demonstrated performances comparable to state of the art ML algorithms^40,41^. The evaluation of both models on MoleculeNet dataset^6^, revealed that graph-based methods generally outperformed descriptor-based methods across most datasets. The superiority of graph-based models was further demonstrated, suggesting that GNN models better capture the structural complexity of chemical compounds^42^. Nonetheless, some studies demonstrated that descriptor-based models outperformed the graph-based models^43^.

Recent research explored the ability of transformer-based architectures in capturing long-range dependencies and contextual information within chemical entities. ChemBERTa, a BERT-based model, was outperformed by baseline models like D-MPNN, RF, and SVM^44^, while its improved version, ChemBERTa2, demonstrated competitive performance against MoleculeNet benchmarks^45^. Other architectures namely, MolFormer, SMILES-BERT and K-BERT were reported to outperform graph-based and descriptor-based models^46–48^. Comparative studies have highlighted that while traditional ML models are still widely used, GNNs and transformers often demonstrated superior performance in handling the complexities of molecular data, with GNNs excelling in spatial representation and transformers offering exceptional performance in capturing contextual information. In the present work, we assessed the performances of multiple ML models, GNN models and transformer-based on four different datasets that differ from benchmark datasets. Our findings revealed that the encoding system and the corresponding trained model constituted a combination with a varying predictive power. Performances of each combination highly depended on the input dataset. Interestingly, the imbalance ratio was an influential parameter in defining the input data, leading to a different set of highly performing models for each disease-related dataset at different IRs. Furthermore, the specificities of the bioassay itself like assay types, target proteins, and the distribution of molecular structures contributed additional biases. Overall, the selection of a model for DD tasks highly depends on the size, the content and the structure of the dataset, highlighting that there is no one-size-fits-all^49^.

We further explored how the intrinsic characteristics of a dataset could influence the predictive process outcomes in the context of biological activity prediction. Through a Chemical Space Networks analysis, we explained the impact of high similarity between compounds from different classes on the rate of misclassified molecules. This highlighted the importance of checking the quality and diversity of the training data, along with the similarity of its class instances with those of the test data. In fact, models trained on compounds with shared structural similarities and holding distinct labels, may struggle to differentiate between the different features of each class and achieve poor performances. Such biases can be overcome through introducing heterogeneity into datasets. Our group previously addressed this challenge through incorporating literature-based compounds or from other sources (or databases) to relatively address the imbalance ratio and to enhance the diversity of the training set, leading to more accurate and robust predictive models^30,50^. The diversity induced by added compounds created a more representative dataset leading the models to learn additional and diverse features.

## Conclusion

Developing a robust AI-driven DD pipeline from chemical datasets presents significant challenges, particularly in achieving accurate predictive models of the biological activity. Our findings emphasize the pivotal role of optimizing both chemical data content and class imbalance to improve model performance. Notably, we revealed that undersampling at a moderate level of IR consistently enhanced models’ performance. Additionally, examining dataset content and chemical similarity between the active and inactive molecules emerges as a crucial step to ensure the reliability and accuracy of the predictive task.

## Methods

### Datasets

All HTS bioassays were retrieved from the PubChem database^51^. We used four primary screening bioassays, each one is associated with a specific Assay Identification Number (AID). These datasets are related to various diseases (Table 3). The HIV datasets is a cell-based assay targeting the HIV Env-mediated cell fusion (Supplementary Table S4). The AID1822 (Malaria) represents a biochemical QFRET-based assay that targets the *Plasmodium falciparum M18 Aspartyl Aminopeptidase (PFM18AAP)*. The third dataset is a luminescence-based biochemical assay targeting *Trypanosoma brucei methionyl-tRNA synthetase (MetRS)*. The COVID-19 bioassay is a biochemical QFRET-based assay targeting the SARS coronavirus *3CL protease (3CLPro)*. All primary screening datasets exhibited large size and high class imbalance (Table 3). Each primary bioassay was paired with its respective confirmatory bioassay, which consists of subsets of molecules that were validated through a second experimental assay. In contrast, datasets of confirmatory bioassays presented smaller sizes and were used for external validation to assess the generalization power and the robustness of the models.

**Table 3:** Summary information of the used bioassays.

Primary AID	Disease	Original IR	Adjusted IR	Size	Active	Inactive	Confirmatory AID	Size
AID651610	HIV	1:90	1:90	354,383	3871	350,512	AID687023	55
AID1822	Malaria	1:82	1:82	290,893	3502	287,391	AID588678	63
AID624268	Trypanosomiasis	1:249	1:100	146,526	1456	145,070	AID651971	249
AID1706	COVID-19	1:717	1:104	114,778	1093	113,685	AID1890	101

The informations presented in this table (number of active, number of inactive and imbalance ratio) are specific to the primary bioassay.

Invalid SMILES were eliminated for each bioassay to ensure that the dataset contained only valid molecular structures. Only molecules with “Active” or “Inactive” labels were included in the input datasets. Additionally, to address the issue of class imbalance, we removed a subset of inactive molecules from certain datasets to achieve an Imbalance Ratio (IR) of approximately 1:100. Table 3 presents both the original IR and the adjusted IR values for each dataset. The adjusted IR reflects the final ratios of the datasets used in training our models. This adjustment was crucial to align the datasets with a similar range of IR, ensuring consistency across the bioassays used in our study.

### Models & encoding systems

Molecules from all bioassays, stored in SMILES format, were converted into specific encoding formats, as required by each of the ML and DL models used here.

- For the ML models, namely Random Forest (RF), Multi-Layer Perceptron (MLP), K-Nearest Neighbors (KNN), eXtreme Gradient Boosting (XGBoost), Naive Bayes (NB), we used the RDKit library^52^ to generate 2048 bit RDKIT fingerprints^53^ as input features.
- For Graph Neural Networks (GNN) models we used graph convolution-based features to represent chemical structures as undirected graphs, where nodes are atoms and edges are bonds. We employed MolGraphConvFeaturizer within the deepchem library^54^ to generate inputs, adjusting its application based on the model type. Graph Convolution Network (GCN)^55^ and Graph Attention Network (GAT)^56^, we set *use_edges* option to false, focusing solely on atom-level information. In contrast, for Attentive FP (AFP)^38^ and Message Passing Neural Network (MPNN)^36^, the *use_edges* option was set to true, incorporating both atom and bond information in the graph-based features.
- For transformer-based models, we tokenized the SMILES strings using the SMILES tokenizer from the Hugging Face Transformer library^57^. This tokenizer converts the SMILES strings into discrete tokens that represent the chemical structure. The resulting tokenized sequences were processed for input into transformer-based models, with padding or truncation to ensure a consistent input length. The models used in this context were ChemBERTa^44^ and MolFormer^46^.

### Data resampling

Datasets were split following an 80/10/10 random split, where the dataset is divided into three subsets as follows: 80% for the training set, 10% for the test set and 10% for the validation set. First, we conducted a series of simulations to evaluate the performance of the aforementioned algorithms when trained on highly imbalanced datasets (Original). Then, we used seven resampling techniques to generate balanced training set: three oversampling methods namely Random OverSampling (ROS), Adaptive Synthetic Sampling Approach for Imbalanced Learning (ADASYN)^58^ and the Synthetic Minority Over-sampling Technique (SMOTE)^23^ and four undersampling methods namely, One-Side Selection (OSS), Neighborhood Cleaning Rule (NCR), Random Undersampling (RUS) and the NearMiss technique^59^. Lastly, we employed a K-Ratio Undersampling which constitutes in randomly reducing samples from the majority class to k times that of the minority class (1:k). In our study, for each bioassay we generated three K-RUS dataset as follows: 1:50, 1:25 and 1:10.

### Evaluation metrics

To evaluate the predictive performance of our models, we assessed various measures. These metrics are derived from the confusion matrix, a table that provides a detailed analysis of the predicted and actual classes.

**Table:** 

	Actual positive	Actual negative
Predicted positive	TP	FP
Predicted negative	FN	TN

The confusion matrix provides four key counts: True Positives (TP), False Positives (FP), True Negatives (TN), and False Negatives (FN), which serve to calculate all metrics.

**Accuracy** measures the proportion of correct predictions out of the total number of predictions.

$$\begin{aligned} Accuracy = \frac{TP + TN}{TP +TN+ FP+FN} \end{aligned}$$

**Balanced accuracy** is a proper metric to evaluate the performance of models dealing with highly imbalanced data^60^. Unlike traditional accuracy, which can be misleading in this scenario. Balanced Accuracy is calculated as the arithmetic mean of sensitivity and specificity.

$$\begin{aligned} Balanced \, accuracy = \frac{1}{2} \left(\frac{TP}{TP + FN} + \frac{TN}{TN + FP} \right) \end{aligned}$$

**Receiver operating characteristic area under the curve (ROC-AUC)** serves as a metric to quantify the ability of a model to distinguish between the positive and negative classes. It involves computing the area under the ROC curve which is obtained by plotting a graphical representation showing the true positive rate (TPR) against the false positive rate (FPR) at various thresholds.

**Matthew’s correlation coefficient (MCC)** measures the quality of binary classification when the dataset is imbalanced. It varies between -1 and +1.

$$\begin{aligned} MCC = \frac{{TP} \times {TN} - {FP} \times {FN}}{\sqrt{({TP} + {FP})({TP} + {FN})({TN} +{FP})({TN} + {FN})}} \end{aligned}$$

**Cohen’s Kappa** measures agreement between two classifiers in qualitative tasks, accounting for chance agreement. Values closer to 1 indicate stronger alignment with the ground truth.

1 $$\begin{aligned} \kappa = \frac{p_o - p_e}{1 - p_e} \end{aligned}$$

where:

- $p_o$ is the **observed agreement** between the two raters (i.e., the proportion of times both raters give the same label).
- $p_e$ is the **expected agreement** by chance, based on the marginal probabilities of each rater.

**Precision** is the proportion of TP predictions out all of positive predictions. A good model should have a high degree of precision to prevent FP predictions.

$$\begin{aligned} Precision = \frac{TP}{TP+FP} \end{aligned}$$

**Recall** is the proportion of TP predictions correctly identified by the model.

$$\begin{aligned} Recall = \frac{TP}{TP+FN} \end{aligned}$$

**F1-score** is the harmonic mean of recall and precision. It combines precision and recall into a single measure to easily determine if a high level of precision and recall are being achieved at the same time.

$$\begin{aligned} F1-score = \frac{2*Precision*Recall}{Precision+Recall} = \frac{2*TP}{2*TP+FP+FN} \end{aligned}$$

From the confusion matrix, we derive two essential metrics to evaluate the predictive performance of our classifiers at the stage of the external validation: **True Positive Rate (TPR)** (known as recall) reflects the model’s capacity to accurately identify positive instances.

$$\begin{aligned} TPR = \frac{TP}{TP+FN} \end{aligned}$$

**False Positive Rate (FPR)** reflects the model’s capacity to classify incorrectly negative instances as positive.

$$\begin{aligned} FPR = \frac{FP}{FP+TN} \end{aligned}$$

### Fine tuning and cross validation

We optimized the top three performing models for each dataset. Hyperparameters leading to optimal predictive performance of each model were identified, through evaluating all possible combinations of parameters within a predefined grid for a given training dataset. For ML models we optimized the hyperparameters specific to the respective model. For Graph-based models, we adjusted parameters such as batch size, number of epochs, dropout rate and the the number of layers. For transformer-based models, the number of epochs, batch size, learning rate, warm-up steps, and weight decay were optimized. Each combination was assessed based on balanced accuracy and F1-score to identify the optimal configuration of hyperparameters for each classifier that yield to the best performance on a specific training set. Additionally, we performed a 5-fold cross-validation to ensure the robustness and generalization power of the models. Datasets were partitioned into five equally sized subsets, where 4 folds were used for training the model, and the remaining fold was reserved for testing. This process was repeated 5 times, ensuring that each fold was used once as the test set. The average of the performance metric and its standard deviation (STD) value across the five simulations were reported to reflect the overall performance. The STD quantifies the variability across the folds, offering insights into the model’s consistency. Additionally, we computed the 95% confidence interval (CI) for the performance metric. The CI offers a statistical range within which the true mean performance is expected to lie with a specified level of confidence. The CI was calculated using the following formula:

$$\begin{aligned} & \text {CI} = \bar{x} \pm z_{\alpha /2} \cdot \frac{s}{\sqrt{n}} \\ & \bar{x} \text { is the average performance,} \\ & s \text { is the standard deviation,} \\ & n \text { is the number of samples} \\ & z_{\alpha /2} \text { is the critical value from the standard normal (Z) distribution for a 95\% confidence level.} \end{aligned}$$

As a last step, we conducted an external validation to assess the optimized models’ ability to predict the biological activity of chemical compounds against each disease using an unseen set of data (confirmatory bioassays). The used confirmatory bioassays are experimentally related to the primary bioassays. Thus, we removed all common molecules between both datasets. At this stage, the optimized models were trained on the entire dataset (primary bioassay) without splitting it into training, validation, or test sets, ensuring that all data contributed to the model learning.

### Chemical space network generation

To assess the structural similarity or dissimilarity between compounds in the training (primary bioassays) and external (confirmatory bioassays) datasets, we employed the Tanimoto similarity metric, a widely used metric for measuring molecular similarity^61^. The Tanimoto coefficient is defined as the fraction of conserved features divided by the total number of features in each molecule, as follows:

$$\begin{aligned} Tanimoto(A, B) = \frac{C}{A + B - C} \ \ ;\ where\ C\ denotes\ the\ common\ features\ between\ A\ and\ B. \end{aligned}$$

For each active and inactive compound in the training set (primary bioassay), we calculated the class similarity to every active and inactive compound in the external validation dataset (confirmatory bioassay). This comparison allowed us to examine structural relationships, specifically between compounds of different activity classes, that may contribute to the performance of predictive models, particularly in terms of misclassification.

To further analyze and visualize these relationships, we constructed Chemical Space Networks (CSNs)^62^ using the NetworkX library^63^. In these networks, each compound was represented as a node, with the shape of the node indicating its origin: square nodes denoting compounds from the training dataset and triangle nodes representing compounds from the external validation dataset. Edges between nodes were weighted based on the Tanimoto similarity values with a threshold applied to include only edges with a similarity score above 0.7. To enhance interpretability, these edges were differentiated using distinct line styles to represent varying ranges of similarity. The nodes and edges were added to the network iteratively. The CSN were designed to represent clusters of structurally similar compounds and to identify factors that may contribute to model misclassifications. Through these networks, we focused on two types of misclassification:

- False Positives: Compounds in the external validation dataset that were predicted as active but were actually inactive. We examined whether these compounds shared high structural similarity with active compounds in the training set.
- False Negatives: Compounds in the external validation dataset that were predicted as inactive but were actually active. We analyzed whether these compounds shared high structural similarity with inactive compounds in the training set.

### Hardware environment

To carry out this project, three main computational environments were used, as reported in Table 4.

**Table 4:** Configuration of hardware environments.

Characteristics	Personal machine	Kaggle notebooks	Tesla server
Processor	Intel Core i7-9750H CPU @ 2.60GHz	Intel Xeon 2.20 GHz (4 vCPU)	Intel Xeon E5-2699 v3 @ 2.30GHz
RAM	16 GB	29 GB	504 GB
GPU	NVIDIA GTX 1650 4 GB	Tesla P100 (16 GB), T4 (16 GB $\times$2)	No GPU

## Supplementary Information

Supplementary Information.

## Data Availability

All data generated and analyzed during this study are correctly cited in this published article through their AID numbers in PubChem and in its supplementary files.
