# Supplementary material for: Adjusted imbalance ratio leads to effective AI-based drug discovery against infectious disease
Source: Sci Rep. 2025 Aug 12;15:29563. doi: 10.1038/s41598-025-15265-5 (PMC12343955; doi:10.1038/s41598-025-15265-5)
Supplement: Supplementary file 1 — Supplementary Information. [file 41598_2025_15265_MOESM1_ESM.pdf]

Adjusted imbalance ratio leads to effective AI-based drug discovery against infectious disease

Authors: Ons Masmoudi[(1,2)], Afef Abdelkrim[(3)],Emna Harigua-Souiai*[(1)]

Supplementary File :

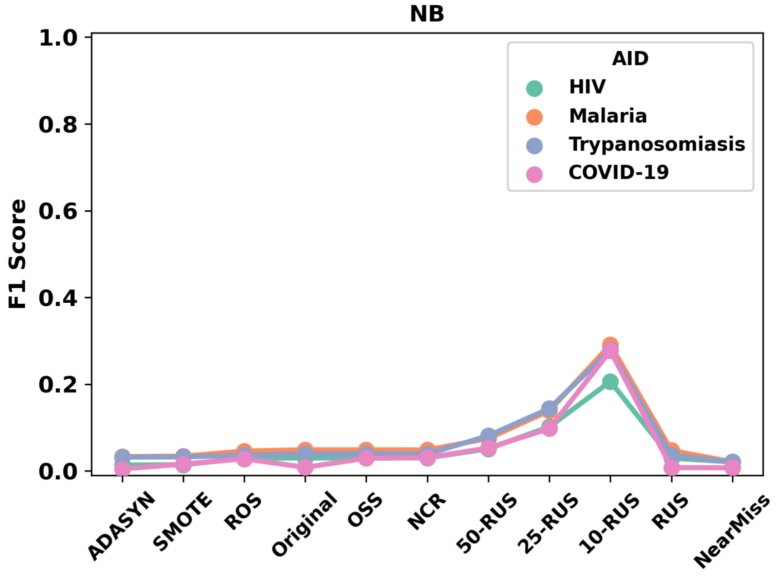

Figure S1 : F1-score comparison of eight algorithms (RF, MLP, GCN, GAT, AFP, MPNN, ChemBERTa, and MolFormer) across the different data states when trained on the different datasets (HIV, Malaria, Trypanosomiasis, and COVID-19

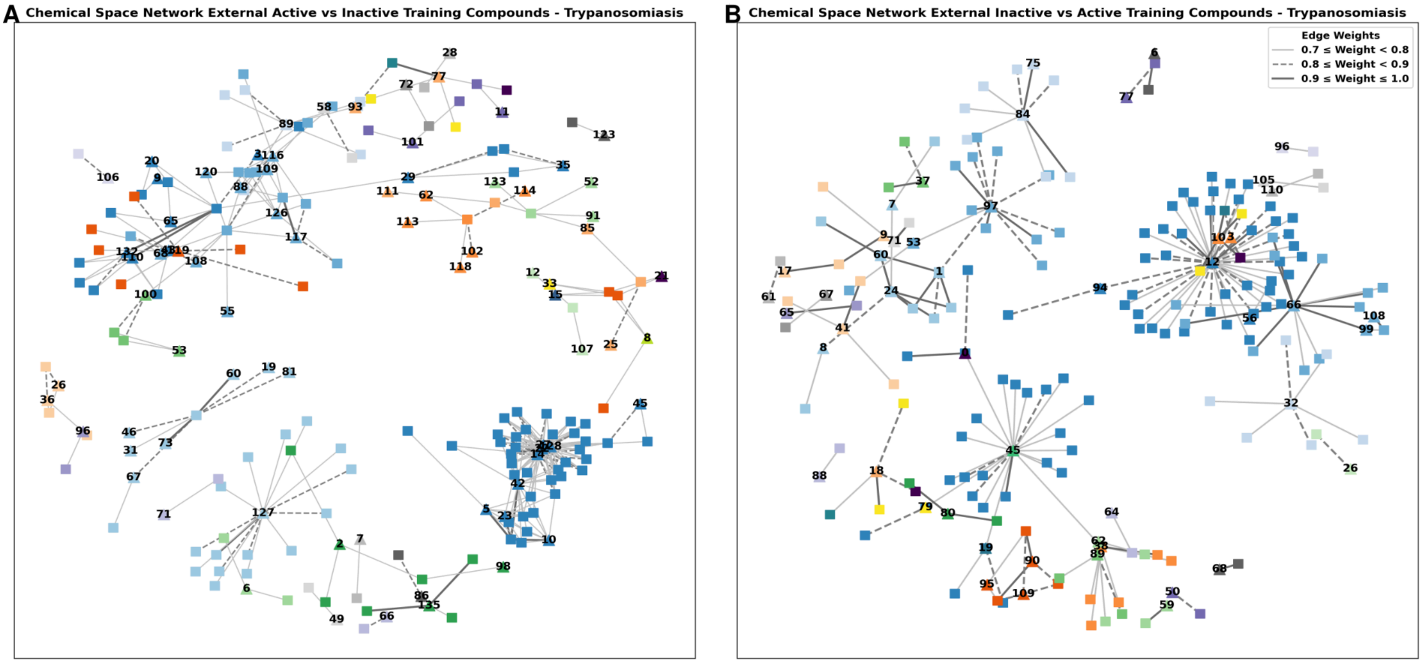

Figure S2 : Chemical Space Network of the compounds in the external dataset and the training set of Trypanosomiasis. (A) Chemical Space Network of the active compounds in the external dataset (triangle) and the inactive compounds in the training set (square). (B) Chemical Space Network of the inactive compounds in the external dataset (triangle) and the active compounds in the training set (square).

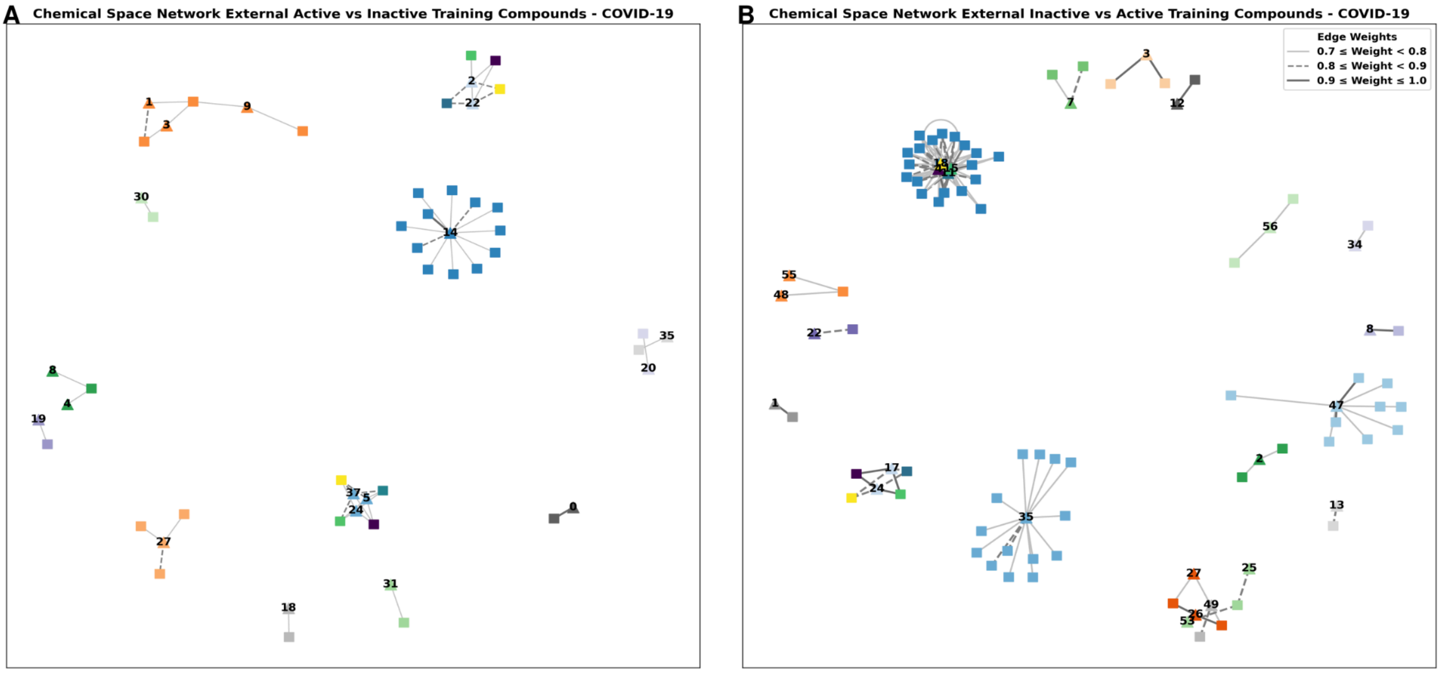

Figure S3 : Chemical Space Network of the compounds in the external dataset and the training set of COVID-19. (A) Chemical Space Network of the active compounds in the external dataset (triangle) and the inactive compounds in the training set (square). (B) Chemical Space Network of the inactive compounds in the external dataset (triangle) and the active compounds in the training set (square).

Supplementary Table S1 : F1-score Values of All Models Across All States

AID	Data state	RF	MLP	KNN	XGBoost	NB	GCN	GAT	AFP	MPNN	ChemBERTa	MolFormer
HIV	ADASYN	0.18	0.18	0.14	0.10	0.01	0.05	0.005	0	0	0	0.03
	SMOTE	0.16	0.18	0.15	0.11	0.01	0.02	0.005	0	0	0.10	0.03
	ROS	0.19	0.18	0.18	0.14	0.03	0.08	0.06	0.13	0.11	0.16	0.20
	Original	0.13	0.14	0.10	0.14	0.03	0.01	0	0	0	0	0.05
	OSS	0.16	0.19	0.14	0.14	0.03	0	0	0	0	0	0.04
	NCR	0.19	0.19	0.18	0.20	0.03	0	0	0	0	0	0.04
	50-RUS	0.15	0.17	0.13	0.12	0.05	0.08	0.005	0.005	0	0.06	0.13
	25-RUS	0.25	0.21	0.23	0.25	0.10	0.08	0.005	0.005	0	0.18	0.12
	10-RUS	0.32	0.35	0.33	0.37	0.20	0.26	0.01	0.33	0.01	0.29	0.23
	RUS	0.33	0.29	0.28	0.29	0.03	0.31	0.29	0.31	0.30	0.04	0.05
	NearMiss	0.02	0.02	0.02	0.02	0.02	0.02	0.02	0.02	0.02	0.02	0.02

AID	Data state	RF	MLP	KNN	XGBoost	NB	GCN	GAT	AFP	MPNN	ChemBERTa	MolFormer
Malaria	ADASYN	0.24	0.39	0.23	0.30	0.03	0.26	0.11	0.04	0	0.06	0.13
	SMOTE	0.24	0.40	0.23	0.30	0.03	0.29	0.18	0.02	0.03	0.35	0.08
	ROS	0.29	0.39	0.28	0.34	0.04	0.16	0.12	0.10	0.26	0.42	0.41
	Original	0.24	0.38	0.25	0.35	0.05	0.30	0.08	0.08	0.03	0.35	0.42
	OSS	0.24	0.38	0.26	0.39	0.05	0.09	0.005	0	0	0	0.38
	NCR	0.30	0.36	0.30	0.40	0.05	0.07	0.08	0	0	0	0.35
	50-RUS	0.30	0.46	0.33	0.41	0.07	0.40	0.11	0.18	0.07	0.36	0.45
	25-RUS	0.38	0.53	0.42	0.53	0.14	0.50	0.12	0.50	0.16	0.49	0.48
	10-RUS	0.47	0.59	0.52	0.60	0.29	0.54	0.17	0.61	0.59	0.62	0.58
	RUS	0.45	0.43	0.36	0.47	0.04	0.53	0.42	0.47	0.44	0.11	0.08
	NearMiss	0.02	0.03	0.03	0.02	0.02	0.02	0.02	0.03	0.02	0.03	0.02

AID	Data state	RF	MLP	KNN	XGBoost	NB	GCN	GAT	AFP	MPNN	ChemBERTa	MolFormer
Trypanosomiasis	ADASYN	0.40	0.47	0.30	0.43	0.03	0.30	0	0	0	0.31	0.19
	SMOTE	0.38	0.42	0.30	0.45	0.03	0.19	0	0	0	0.02	0.14
	ROS	0.45	0.48	0.41	0.53	0.04	0.23	0.07	0.33	0.29	0.43	0.45
	Original	0.35	0.45	0.33	0.48	0.04	0.19	0	0	0	0.33	0.31
	OSS	0.36	0.49	0.36	0.47	0.04	0	0	0	0	0.20	0.20
	NCR	0.42	0.50	0.42	0.50	0.04	0	0	0	0	0.32	0.20
	50-RUS	0.44	0.54	0.47	0.52	0.08	0.13	0	0.01	0	0.40	0.46
	25-RUS	0.44	0.56	0.48	0.56	0.14	0.55	0.01	0.54	0	0.47	0.52
	10-RUS	0.56	0.61	0.60	0.68	0.28	0.57	0	0.64	0.56	0.59	0.53
	RUS	0.57	0.57	0.33	0.47	0.04	0.53	0.36	0.50	0.52	0.06	0.09
	NearMiss	0.02	0.03	0.02	0.02	0.02	0.02	0.02	0.02	0.02	0.02	0.02

AID	Data state	RF	MLP	KNN	XGBoost	NB	GCN	GAT	AFP	MPNN	ChemBERTa	MolFormer
COVID-19	ADASYN	0.10	0.12	0.09	0.16	0.004	0.40	0.14	0.06	0	0.51	0.02
	SMOTE	0.37	0.41	0.19	0.35	0.01	0.32	0.14	0.04	0	0.49	0.01
	ROS	0.37	0.41	0.29	0.41	0.03	0.17	0.06	0.44	0.28	0.05	0.20
	Original	0.32	0.38	0.16	0.15	0.008	0.46	0.10	0.09	0	0	0.52
	OSS	0.35	0.40	0.34	0.38	0.03	0.07	0.04	0	0	0.51	0.02
	NCR	0.33	0.37	0.38	0.37	0.03	0.02	0.07	0	0	0.52	0.02
	50-RUS	0.40	0.42	0.33	0.44	0.05	0.49	0.18	0.43	0	0	0.52
	25-RUS	0.33	0.43	0.37	0.40	0.1	0.39	0.20	0.50	0.35	0.05	0.64
	10-RUS	0.50	0.55	0.48	0.53	0.27	0.56	0.20	0.60	0.55	0.12	0.59
	RUS	0.42	0.35	0.09	0.13	0.007	0.37	0.24	0.36	0.33	0.008	0.02
	NearMiss	0.02	0.02	0.007	0.005	0.007	0.02	0.02	0.02	0.02	0.013	0.01

Supplementary Table S2 : Hyperparameters Tuning and Cross-Validation Summary

XGBoost trained on HIV dataset

Learning_rate	n_estimators	subsample	max_depth	colsample_bytree	Balanced Accuracy	F1 -score
1	50	1	6	1	**0.63**	**0.37**
1	50	1	6	0.5	0.63	0.36
1	20	1	6	1	0.62	0.35
1	50	0.8	6	0.8	0.62	0.34
0.5	50	0.8	6	1	0.62	0.34
1	50	1	6	0.8	0.62	0.34
0.5	50	0.8	6	0.8	0.61	0.34
0.5	50	1	6	0.8	0.61	0.34
1	20	0.8	6	1	0.62	0.33
1	20	1	6	0.5	0.62	0.33
0.5	50	0.5	6	1	0.62	0.33
1	50	0.8	6	0.5	0.62	0.33
0.5	50	0.5	6	0.5	0.61	0.33
0.5	50	0.5	6	0.8	0.61	0.33
0.5	50	1	6	0.5	0.61	0.33
0.5	50	1	6	1	0.61	0.33
1	50	0.8	6	1	0.61	0.32
1	10	1	6	1	0.6	0.32
0.25	50	0.5	6	0.5	0.6	0.32
0.25	50	0.8	6	1	0.6	0.32
0.25	50	1	6	0.8	0.6	0.32
1	20	1	6	0.8	0.61	0.31
1	8	0.8	6	1	0.6	0.31
1	10	0.8	6	1	0.6	0.31
0.5	20	0.5	6	1	0.6	0.31
0.5	20	0.8	6	1	0.6	0.31
1	50	1	4	0.5	0.6	0.31
0.25	50	0.8	6	0.5	0.59	0.31
1	50	0.5	4	0.8	0.61	0.3
1	50	0.5	6	0.5	0.61	0.3
1	20	0.8	6	0.8	0.6	0.3
1	50	1	4	1	0.6	0.3
0.25	50	0.5	6	0.8	0.59	0.3
0.25	50	0.5	6	1	0.59	0.3
0.25	50	1	6	1	0.59	0.3
1	20	0.5	6	1	0.61	0.29
1	50	0.5	6	0.8	0.61	0.29
1	20	0.8	6	0.5	0.6	0.29
1	50	0.5	4	0.5	0.6	0.29
1	50	0.8	4	0.8	0.6	0.29
1	10	1	6	0.5	0.59	0.29
1	50	0.8	4	1	0.59	0.29
1	50	1	4	0.8	0.59	0.29
0.5	50	0.8	6	0.5	0.59	0.29
0.25	50	1	6	0.5	0.59	0.29
1	50	0.5	6	1	0.61	0.28
1	20	0.5	6	0.5	0.6	0.28
1	8	1	6	1	0.59	0.28
1	10	0.8	6	0.5	0.59	0.28
0.5	20	1	6	0.8	0.59	0.28
0.5	20	1	6	1	0.59	0.28
0.25	50	0.8	6	0.8	0.58	0.28
1	10	0.5	6	0.8	0.59	0.27
1	10	1	6	0.8	0.59	0.27
1	20	0.5	6	0.8	0.59	0.27
1	50	0.5	4	1	0.59	0.27
1	50	0.8	4	0.5	0.59	0.27
1	8	0.8	6	0.5	0.58	0.27
1	20	1	4	0.5	0.58	0.27
0.5	20	0.8	6	0.5	0.58	0.27
0.5	50	0.8	4	0.5	0.58	0.27
0.5	50	1	4	1	0.58	0.27
1	8	1	6	0.5	0.58	0.26
1	10	0.8	6	0.8	0.58	0.26
0.5	20	0.8	6	0.8	0.58	0.26
0.5	20	1	6	0.5	0.58	0.26
0.5	50	1	4	0.5	0.58	0.26
0.5	50	1	4	0.8	0.58	0.26
1	8	0.5	6	0.8	0.58	0.25
1	8	1	6	0.8	0.58	0.25
1	20	0.5	4	0.8	0.58	0.25
0.5	20	0.5	6	0.5	0.58	0.25
0.5	50	0.5	4	0.8	0.58	0.25
0.5	50	0.5	4	1	0.58	0.25
1	20	1	4	0.8	0.57	0.25
0.5	20	0.5	6	0.8	0.57	0.25
0.5	50	0.5	4	0.5	0.57	0.25
0.5	50	0.8	4	1	0.57	0.25
1	10	0.5	6	0.5	0.58	0.24
1	8	0.5	6	1	0.57	0.24
1	8	0.8	6	0.8	0.57	0.24
0.5	10	0.5	6	1	0.57	0.24
1	20	0.5	4	0.5	0.57	0.24
1	20	0.8	4	1	0.57	0.24
0.5	50	0.8	4	0.8	0.57	0.24
0.1	50	0.5	6	0.8	0.57	0.24
1	8	0.5	6	0.5	0.57	0.23
0.5	10	0.5	6	0.8	0.57	0.23
1	10	0.5	6	1	0.57	0.23
0.5	10	0.8	6	1	0.57	0.23
1	20	0.5	4	1	0.57	0.23
1	20	0.8	4	0.8	0.57	0.23
1	20	1	4	1	0.57	0.22
1	10	0.8	4	0.8	0.56	0.22
0.5	10	0.5	6	0.5	0.56	0.22
0.25	20	0.5	6	0.5	0.56	0.22
0.25	20	0.5	6	1	0.56	0.22
0.25	20	0.8	6	1	0.56	0.22
0.25	20	1	6	0.5	0.56	0.22
0.25	20	1	6	1	0.56	0.22
0.25	50	0.5	4	1	0.56	0.22
0.1	50	0.8	6	1	0.56	0.22
1	2	1	6	0.5	0.56	0.21
0.5	8	0.8	6	1	0.56	0.21
1	10	0.5	4	0.8	0.56	0.21
0.5	10	0.8	6	0.8	0.56	0.21
0.5	10	1	6	0.8	0.56	0.21
0.5	10	1	6	1	0.56	0.21
1	20	0.8	4	0.5	0.56	0.21
0.1	50	1	6	0.8	0.56	0.21
0.5	8	0.5	6	1	0.56	0.2
0.5	8	0.8	6	0.8	0.56	0.2
0.25	50	0.5	4	0.5	0.56	0.2
0.1	50	0.5	6	1	0.56	0.2
1	2	0.8	6	1	0.55	0.2
1	10	1	4	0.5	0.55	0.2
0.5	10	0.8	6	0.5	0.55	0.2
1	2	0.5	6	0.5	0.55	0.19
1	2	0.5	6	0.8	0.55	0.19
1	2	0.8	6	0.5	0.55	0.19
1	2	1	6	0.8	0.55	0.19
1	8	0.5	4	0.8	0.55	0.19
0.5	8	0.8	6	0.5	0.55	0.19
0.5	8	1	6	0.8	0.55	0.19
0.5	8	1	6	1	0.55	0.19
1	10	0.5	4	0.5	0.55	0.19
1	10	1	4	1	0.55	0.19
0.5	20	0.5	4	0.8	0.55	0.19

Cross validation :

XGBoost : learning_rate = 1, n_estimators = 50, subsample= 1, max_depth = 6, colsample_bytree = 1

K	Test Accuracy	Test ROC-AUC	Test Balanced_acc	Test MCC	Test Precision	Test Recall	Test F1 score	Test Cohen's Kappa
1	0.91	0.73	0.61	0.29	0.49	0.3	0.37	0.28
2	0.9	0.76	0.63	0.31	0.45	0.29	0.35	0.3
3	0.9	0.75	0.63	0.32	0.46	0.3	0.37	0.32
4	0.91	0.77	0.63	0.32	0.45	0.3	0.36	0.31
5	0.91	0.74	0.63	0.32	0.5	0.31	0.38	0.31
Mean	0.90	0.75	0.63	0.31	0.47	0.3	0.37	0.30
SD	0.005	0.016	0.009	0.013	0.023	0.007	0.011	0.015
IC	0.90 ± 4.74E-5	0.75 ±1.5E-4	0.63 ±8.532E-5	0.31 ±1.2E−4	0.47 ±2.2E−4	0.3 ±6.6360E-5	0.37 ±1.0E-4	0.30 ±1.4E-4

0.5	20	0.8	4	1	0.55	0.19
0.25	20	0.8	6	0.8	0.55	0.19
0.25	20	1	6	0.8	0.55	0.19
1	50	0.8	2	1	0.55	0.19
1	50	1	2	0.5	0.55	0.19
0.25	50	0.8	4	0.8	0.55	0.19
0.25	50	1	4	0.5	0.55	0.19
0.25	50	1	4	0.8	0.55	0.19
0.1	50	0.5	6	0.5	0.55	0.19
0.1	50	0.8	6	0.8	0.55	0.19
0.1	50	1	6	0.5	0.55	0.19
0.1	50	1	6	1	0.55	0.19
1	2	0.5	6	1	0.55	0.18
0.5	8	0.5	6	0.8	0.55	0.18
1	10	0.8	4	1	0.55	0.18
1	10	1	4	0.8	0.55	0.18
0.5	10	1	6	0.5	0.55	0.18
0.5	20	0.8	4	0.8	0.55	0.18
1	50	0.5	2	0.8	0.55	0.18
1	50	0.5	2	1	0.55	0.18
1	50	1	2	1	0.55	0.18
0.25	50	0.8	4	1	0.55	0.18
0.25	50	1	4	1	0.55	0.18
0.1	50	0.8	6	0.5	0.55	0.18
1	2	0.8	6	0.8	0.55	0.17
1	8	0.5	4	1	0.55	0.17
1	8	0.8	4	0.8	0.55	0.17
1	8	1	4	1	0.55	0.17
0.5	8	1	6	0.5	0.55	0.17
0.25	20	0.5	6	0.8	0.55	0.17
0.25	20	0.8	6	0.5	0.55	0.17
0.25	50	0.5	4	0.8	0.55	0.17
0.25	50	0.8	4	0.5	0.55	0.17
1	8	1	4	0.8	0.54	0.17
0.5	20	0.5	4	0.5	0.54	0.17
0.5	20	0.5	4	1	0.54	0.17
1	50	0.5	2	0.5	0.54	0.17
1	8	0.5	4	0.5	0.54	0.16
1	8	0.8	4	0.5	0.54	0.16
1	8	1	4	0.5	0.54	0.16
0.5	8	0.5	6	0.5	0.54	0.16
1	10	0.5	4	1	0.54	0.16
1	10	0.8	4	0.5	0.54	0.16
0.5	20	1	4	0.5	0.54	0.16
0.5	20	1	4	1	0.54	0.16
1	50	0.8	2	0.8	0.54	0.16
1	50	1	2	0.8	0.54	0.16
1	2	1	6	1	0.54	0.15
0.5	20	0.8	4	0.5	0.54	0.15
0.5	20	1	4	0.8	0.54	0.15
1	50	0.8	2	0.5	0.54	0.15
0.5	10	0.5	4	0.8	0.54	0.14
0.5	50	0.5	2	1	0.54	0.14
1	8	0.8	4	1	0.53	0.13
0.5	10	0.8	4	0.8	0.53	0.13
0.25	10	0.5	6	1	0.53	0.13
0.25	10	1	6	1	0.53	0.13
1	20	0.5	2	1	0.53	0.13
1	20	0.8	2	1	0.53	0.13
0.5	10	0.5	4	0.5	0.53	0.12
0.5	10	0.8	4	1	0.53	0.12
0.25	10	0.5	6	0.5	0.53	0.12
0.25	10	0.8	6	1	0.53	0.12
0.25	10	0.8	6	0.8	0.53	0.11
0.25	10	1	6	0.5	0.53	0.11
0.5	50	0.5	2	0.8	0.53	0.11
0.5	50	0.8	2	0.5	0.53	0.11
0.5	2	0.5	6	0.8	0.53	0.1
0.5	8	0.5	4	0.5	0.53	0.1
0.25	8	0.5	6	0.5	0.53	0.1
0.25	8	0.5	6	1	0.53	0.1
0.25	8	0.8	6	0.8	0.53	0.1
0.25	8	0.8	6	1	0.53	0.1
0.25	10	0.5	6	0.8	0.53	0.1
0.25	10	1	6	0.8	0.53	0.1
0.25	20	1	4	1	0.53	0.1
0.5	50	1	2	1	0.53	0.1
0.5	2	0.5	6	0.5	0.52	0.1
0.5	10	0.8	4	0.5	0.52	0.1
1	20	0.5	2	0.8	0.52	0.1
1	20	1	2	0.5	0.52	0.1
1	20	1	2	0.8	0.52	0.1
1	20	1	2	1	0.52	0.1
0.5	50	0.8	2	1	0.52	0.1
0.5	2	1	6	1	0.52	0.09
0.5	8	0.5	4	0.8	0.52	0.09
0.5	8	0.8	4	0.5	0.52	0.09
0.25	8	1	6	0.8	0.52	0.09
0.25	8	1	6	1	0.52	0.09
0.5	10	0.5	4	1	0.52	0.09
0.5	10	1	4	0.5	0.52	0.09
0.5	10	1	4	0.8	0.52	0.09
0.5	10	1	4	1	0.52	0.09
1	20	0.8	2	0.5	0.52	0.09
1	20	0.8	2	0.8	0.52	0.09
0.25	20	0.5	4	0.5	0.52	0.09
0.25	20	0.5	4	1	0.52	0.09
0.25	20	0.8	4	0.8	0.52	0.09
0.1	20	0.8	6	0.8	0.52	0.09
0.5	50	0.8	2	0.8	0.52	0.09
0.5	50	1	2	0.5	0.52	0.09
0.5	8	1	4	0.5	0.52	0.08
0.25	8	0.5	6	0.8	0.52	0.08
0.25	10	0.8	6	0.5	0.52	0.08
0.25	20	0.8	4	1	0.52	0.08
0.5	50	0.5	2	0.5	0.52	0.08
0.1	50	0.5	4	0.8	0.52	0.08
0.1	50	0.8	4	1	0.52	0.08
0.1	50	1	4	1	0.52	0.08
1	2	1	4	0.5	0.52	0.07
0.5	8	0.5	4	1	0.52	0.07
0.5	8	0.8	4	0.8	0.52	0.07
0.5	8	0.8	4	1	0.52	0.07
0.5	8	1	4	0.8	0.52	0.07
0.25	8	1	6	0.5	0.52	0.07
1	10	0.5	2	1	0.52	0.07
0.25	20	0.8	4	0.5	0.52	0.07
0.1	20	0.5	6	0.8	0.52	0.07
0.1	20	0.5	6	1	0.52	0.07
0.1	20	0.8	6	0.5	0.52	0.07
0.1	20	0.8	6	1	0.52	0.07
0.1	20	1	6	0.8	0.52	0.07
0.5	50	1	2	0.8	0.52	0.07
0.1	50	0.5	4	1	0.52	0.07
0.1	50	1	4	0.8	0.52	0.07
1	2	0.5	4	1	0.52	0.06
0.5	2	1	6	0.5	0.52	0.06
0.25	20	0.5	4	0.8	0.52	0.06
0.25	20	1	4	0.5	0.52	0.06
0.1	50	0.8	4	0.5	0.52	0.06
0.1	50	0.8	4	0.8	0.52	0.06
0.5	2	0.8	6	0.5	0.51	0.06
0.5	2	0.8	6	1	0.51	0.06
1	8	1	2	0.5	0.51	0.06
1	10	0.8	2	1	0.51	0.06

1	20	0.5	2	0.5	0.51	0.06
0.25	20	1	4	0.8	0.51	0.06
0.1	20	0.5	6	0.5	0.51	0.06
0.1	20	1	6	0.5	0.51	0.06
0.1	20	1	6	1	0.51	0.06
0.1	50	0.5	4	0.5	0.51	0.06
0.1	50	1	4	0.5	0.51	0.06
1	2	0.5	4	0.8	0.51	0.05
0.5	2	0.8	6	0.8	0.51	0.05
0.5	8	1	4	1	0.51	0.05
0.25	8	0.8	6	0.5	0.51	0.05
1	10	0.8	2	0.8	0.51	0.05
1	10	1	2	0.5	0.51	0.05
1	2	0.8	4	0.8	0.51	0.04
1	10	0.5	2	0.5	0.51	0.04
1	10	1	2	1	0.51	0.04
0.5	20	0.5	2	0.5	0.51	0.04
0.5	20	0.5	2	1	0.51	0.04
0.5	20	0.8	2	0.5	0.51	0.04
0.25	50	0.5	2	1	0.51	0.04
0.25	50	0.8	2	0.5	0.51	0.04
1	2	1	4	0.8	0.51	0.03
0.5	2	0.5	6	1	0.51	0.03
1	8	0.5	2	0.8	0.51	0.03
1	8	0.8	2	0.8	0.51	0.03
1	8	0.8	2	1	0.51	0.03
1	8	1	2	1	0.51	0.03
1	10	0.8	2	0.5	0.51	0.03
1	10	1	2	0.8	0.51	0.03
0.5	20	1	2	1	0.51	0.03
0.25	50	0.5	2	0.5	0.51	0.03
0.25	50	0.5	2	0.8	0.51	0.03
0.25	50	0.8	2	0.8	0.51	0.03
0.25	50	1	2	1	0.51	0.03
0.25	10	1	4	0.8	0.51	0.02
0.25	50	0.8	2	1	0.51	0.02
0.25	50	1	2	0.8	0.51	0.02
1	8	0.5	2	1	0.5	0.02
0.25	10	1	4	1	0.5	0.02
0.5	20	1	2	0.5	0.5	0.02
1	2	0.8	4	0.5	0.5	0.01
1	2	1	4	1	0.5	0.01
1	8	0.5	2	0.5	0.5	0.01
1	8	1	2	0.8	0.5	0.01
0.25	8	0.5	4	0.5	0.5	0.01
1	10	0.5	2	0.8	0.5	0.01
0.25	10	0.5	4	0.5	0.5	0.01
0.25	10	0.5	4	0.8	0.5	0.01
0.25	10	1	4	0.5	0.5	0.01
0.1	10	0.5	6	0.8	0.5	0.01
0.5	20	0.5	2	0.8	0.5	0.01
0.5	20	0.8	2	0.8	0.5	0.01
0.5	20	1	2	0.8	0.5	0.01
0.25	50	1	2	0.5	0.5	0.01
0.01	2	0.5	2	0.5	0.5	0
0.1	2	0.5	2	0.5	0.5	0
0.25	2	0.5	2	0.5	0.5	0
0.5	2	0.5	2	0.5	0.5	0
1	2	0.5	2	0.5	0.5	0
0.01	2	0.5	2	0.8	0.5	0
0.1	2	0.5	2	0.8	0.5	0
0.25	2	0.5	2	0.8	0.5	0
0.5	2	0.5	2	0.8	0.5	0
1	2	0.5	2	0.8	0.5	0
0.01	2	0.5	2	1	0.5	0
0.1	2	0.5	2	1	0.5	0
0.25	2	0.5	2	1	0.5	0
0.5	2	0.5	2	1	0.5	0
1	2	0.5	2	1	0.5	0
0.01	2	0.8	2	0.5	0.5	0
0.1	2	0.8	2	0.5	0.5	0
0.25	2	0.8	2	0.5	0.5	0
0.5	2	0.8	2	0.5	0.5	0
1	2	0.8	2	0.5	0.5	0
0.01	2	0.8	2	0.8	0.5	0
0.1	2	0.8	2	0.8	0.5	0
0.25	2	0.8	2	0.8	0.5	0
0.5	2	0.8	2	0.8	0.5	0
1	2	0.8	2	0.8	0.5	0
0.01	2	0.8	2	1	0.5	0
0.1	2	0.8	2	1	0.5	0
0.25	2	0.8	2	1	0.5	0
0.5	2	0.8	2	1	0.5	0
1	2	0.8	2	1	0.5	0
0.01	2	1	2	0.5	0.5	0
0.1	2	1	2	0.5	0.5	0
0.25	2	1	2	0.5	0.5	0
0.5	2	1	2	0.5	0.5	0
1	2	1	2	0.5	0.5	0
0.01	2	1	2	0.8	0.5	0
0.1	2	1	2	0.8	0.5	0
0.25	2	1	2	0.8	0.5	0
0.5	2	1	2	0.8	0.5	0
1	2	1	2	0.8	0.5	0
0.01	2	1	2	1	0.5	0
0.1	2	1	2	1	0.5	0
0.25	2	1	2	1	0.5	0
0.5	2	1	2	1	0.5	0
1	2	1	2	1	0.5	0
0.01	2	0.5	4	0.5	0.5	0
0.1	2	0.5	4	0.5	0.5	0
0.25	2	0.5	4	0.5	0.5	0
0.5	2	0.5	4	0.5	0.5	0
1	2	0.5	4	0.5	0.5	0
0.01	2	0.5	4	0.8	0.5	0
0.1	2	0.5	4	0.8	0.5	0
0.25	2	0.5	4	0.8	0.5	0
0.5	2	0.5	4	0.8	0.5	0
0.01	2	0.5	4	1	0.5	0
0.1	2	0.5	4	1	0.5	0
0.25	2	0.5	4	1	0.5	0
0.5	2	0.5	4	1	0.5	0
0.01	2	0.8	4	0.5	0.5	0
0.1	2	0.8	4	0.5	0.5	0
0.25	2	0.8	4	0.5	0.5	0
0.5	2	0.8	4	0.5	0.5	0
0.01	2	0.8	4	0.8	0.5	0
0.1	2	0.8	4	0.8	0.5	0
0.25	2	0.8	4	0.8	0.5	0
0.5	2	0.8	4	0.8	0.5	0
0.01	2	0.8	4	1	0.5	0
0.1	2	0.8	4	1	0.5	0
0.25	2	0.8	4	1	0.5	0
0.5	2	0.8	4	1	0.5	0
1	2	0.8	4	1	0.5	0
0.01	2	1	4	0.5	0.5	0
0.1	2	1	4	0.5	0.5	0
0.25	2	1	4	0.5	0.5	0
0.5	2	1	4	0.5	0.5	0
0.01	2	1	4	0.8	0.5	0
0.1	2	1	4	0.8	0.5	0
0.25	2	1	4	0.8	0.5	0
0.5	2	1	4	0.8	0.5	0
0.01	2	1	4	1	0.5	0
0.1	2	1	4	1	0.5	0

0.25	2	1	4	1	0.5	0
0.5	2	1	4	1	0.5	0
0.01	2	0.5	6	0.5	0.5	0
0.1	2	0.5	6	0.5	0.5	0
0.25	2	0.5	6	0.5	0.5	0
0.01	2	0.5	6	0.8	0.5	0
0.1	2	0.5	6	0.8	0.5	0
0.25	2	0.5	6	0.8	0.5	0
0.01	2	0.5	6	1	0.5	0
0.1	2	0.5	6	1	0.5	0
0.25	2	0.5	6	1	0.5	0
0.01	2	0.8	6	0.5	0.5	0
0.1	2	0.8	6	0.5	0.5	0
0.25	2	0.8	6	0.5	0.5	0
0.01	2	0.8	6	0.8	0.5	0
0.1	2	0.8	6	0.8	0.5	0
0.25	2	0.8	6	0.8	0.5	0
0.01	2	0.8	6	1	0.5	0
0.1	2	0.8	6	1	0.5	0
0.25	2	0.8	6	1	0.5	0
0.01	2	1	6	0.5	0.5	0
0.1	2	1	6	0.5	0.5	0
0.25	2	1	6	0.5	0.5	0
0.01	2	1	6	0.8	0.5	0
0.1	2	1	6	0.8	0.5	0
0.25	2	1	6	0.8	0.5	0
0.5	2	1	6	0.8	0.5	0
0.01	2	1	6	1	0.5	0
0.1	2	1	6	1	0.5	0
0.25	2	1	6	1	0.5	0
0.01	8	0.5	2	0.5	0.5	0
0.1	8	0.5	2	0.5	0.5	0
0.25	8	0.5	2	0.5	0.5	0
0.5	8	0.5	2	0.5	0.5	0
0.01	8	0.5	2	0.8	0.5	0
0.1	8	0.5	2	0.8	0.5	0
0.25	8	0.5	2	0.8	0.5	0
0.5	8	0.5	2	0.8	0.5	0
0.01	8	0.5	2	1	0.5	0
0.1	8	0.5	2	1	0.5	0
0.25	8	0.5	2	1	0.5	0
0.5	8	0.5	2	1	0.5	0
0.01	8	0.8	2	0.5	0.5	0
0.1	8	0.8	2	0.5	0.5	0
0.25	8	0.8	2	0.5	0.5	0
0.5	8	0.8	2	0.5	0.5	0
1	8	0.8	2	0.5	0.5	0
0.01	8	0.8	2	0.8	0.5	0
0.1	8	0.8	2	0.8	0.5	0
0.25	8	0.8	2	0.8	0.5	0
0.5	8	0.8	2	0.8	0.5	0
0.01	8	0.8	2	1	0.5	0
0.1	8	0.8	2	1	0.5	0
0.25	8	0.8	2	1	0.5	0
0.5	8	0.8	2	1	0.5	0
0.01	8	1	2	0.5	0.5	0
0.1	8	1	2	0.5	0.5	0
0.25	8	1	2	0.5	0.5	0
0.5	8	1	2	0.5	0.5	0
0.01	8	1	2	0.8	0.5	0
0.1	8	1	2	0.8	0.5	0
0.25	8	1	2	0.8	0.5	0
0.5	8	1	2	0.8	0.5	0
0.01	8	1	2	1	0.5	0
0.1	8	1	2	1	0.5	0
0.25	8	1	2	1	0.5	0
0.5	8	1	2	1	0.5	0
0.01	8	0.5	4	0.5	0.5	0
0.1	8	0.5	4	0.5	0.5	0
0.01	8	0.5	4	0.8	0.5	0
0.1	8	0.5	4	0.8	0.5	0
0.25	8	0.5	4	0.8	0.5	0
0.01	8	0.5	4	1	0.5	0
0.1	8	0.5	4	1	0.5	0
0.25	8	0.5	4	1	0.5	0
0.01	8	0.8	4	0.5	0.5	0
0.1	8	0.8	4	0.5	0.5	0
0.25	8	0.8	4	0.5	0.5	0
0.01	8	0.8	4	0.8	0.5	0
0.1	8	0.8	4	0.8	0.5	0
0.25	8	0.8	4	0.8	0.5	0
0.01	8	0.8	4	1	0.5	0
0.1	8	0.8	4	1	0.5	0
0.25	8	0.8	4	1	0.5	0
0.01	8	1	4	0.5	0.5	0
0.1	8	1	4	0.5	0.5	0
0.25	8	1	4	0.5	0.5	0
0.01	8	1	4	0.8	0.5	0
0.1	8	1	4	0.8	0.5	0
0.25	8	1	4	0.8	0.5	0
0.01	8	1	4	1	0.5	0
0.1	8	1	4	1	0.5	0
0.25	8	1	4	1	0.5	0
0.01	8	0.5	6	0.5	0.5	0
0.1	8	0.5	6	0.5	0.5	0
0.01	8	0.5	6	0.8	0.5	0
0.1	8	0.5	6	0.8	0.5	0
0.01	8	0.5	6	1	0.5	0
0.1	8	0.5	6	1	0.5	0
0.01	8	0.8	6	0.5	0.5	0
0.1	8	0.8	6	0.5	0.5	0
0.01	8	0.8	6	0.8	0.5	0
0.1	8	0.8	6	0.8	0.5	0
0.01	8	0.8	6	1	0.5	0
0.1	8	0.8	6	1	0.5	0
0.01	8	1	6	0.5	0.5	0
0.1	8	1	6	0.5	0.5	0
0.01	8	1	6	0.8	0.5	0
0.1	8	1	6	0.8	0.5	0
0.01	8	1	6	1	0.5	0
0.1	8	1	6	1	0.5	0
0.01	10	0.5	2	0.5	0.5	0
0.1	10	0.5	2	0.5	0.5	0
0.25	10	0.5	2	0.5	0.5	0
0.5	10	0.5	2	0.5	0.5	0
0.01	10	0.5	2	0.8	0.5	0
0.1	10	0.5	2	0.8	0.5	0
0.25	10	0.5	2	0.8	0.5	0
0.5	10	0.5	2	0.8	0.5	0
0.01	10	0.5	2	1	0.5	0
0.1	10	0.5	2	1	0.5	0
0.25	10	0.5	2	1	0.5	0
0.5	10	0.5	2	1	0.5	0
0.01	10	0.8	2	0.5	0.5	0
0.1	10	0.8	2	0.5	0.5	0
0.25	10	0.8	2	0.5	0.5	0
0.5	10	0.8	2	0.5	0.5	0
0.01	10	0.8	2	0.8	0.5	0
0.1	10	0.8	2	0.8	0.5	0
0.25	10	0.8	2	0.8	0.5	0
0.5	10	0.8	2	0.8	0.5	0
0.01	10	0.8	2	1	0.5	0
0.1	10	0.8	2	1	0.5	0
0.25	10	0.8	2	1	0.5	0
0.5	10	0.8	2	1	0.5	0

0.01	10	1	2	0.5	0.5	0
0.1	10	1	2	0.5	0.5	0
0.25	10	1	2	0.5	0.5	0
0.5	10	1	2	0.5	0.5	0
0.01	10	1	2	0.8	0.5	0
0.1	10	1	2	0.8	0.5	0
0.25	10	1	2	0.8	0.5	0
0.5	10	1	2	0.8	0.5	0
0.01	10	1	2	1	0.5	0
0.1	10	1	2	1	0.5	0
0.25	10	1	2	1	0.5	0
0.5	10	1	2	1	0.5	0
0.01	10	0.5	4	0.5	0.5	0
0.1	10	0.5	4	0.5	0.5	0
0.01	10	0.5	4	0.8	0.5	0
0.1	10	0.5	4	0.8	0.5	0
0.01	10	0.5	4	1	0.5	0
0.1	10	0.5	4	1	0.5	0
0.25	10	0.5	4	1	0.5	0
0.01	10	0.8	4	0.5	0.5	0
0.1	10	0.8	4	0.5	0.5	0
0.25	10	0.8	4	0.5	0.5	0
0.01	10	0.8	4	0.8	0.5	0
0.1	10	0.8	4	0.8	0.5	0
0.25	10	0.8	4	0.8	0.5	0
0.01	10	0.8	4	1	0.5	0
0.1	10	0.8	4	1	0.5	0
0.25	10	0.8	4	1	0.5	0
0.01	10	1	4	0.5	0.5	0
0.1	10	1	4	0.5	0.5	0
0.01	10	1	4	0.8	0.5	0
0.1	10	1	4	0.8	0.5	0
0.01	10	1	4	1	0.5	0
0.1	10	1	4	1	0.5	0
0.01	10	0.5	6	0.5	0.5	0
0.1	10	0.5	6	0.5	0.5	0
0.01	10	0.5	6	0.8	0.5	0
0.01	10	0.5	6	1	0.5	0
0.1	10	0.5	6	1	0.5	0
0.01	10	0.8	6	0.5	0.5	0
0.1	10	0.8	6	0.5	0.5	0
0.01	10	0.8	6	0.8	0.5	0
0.1	10	0.8	6	0.8	0.5	0
0.01	10	0.8	6	1	0.5	0
0.1	10	0.8	6	1	0.5	0
0.01	10	1	6	0.5	0.5	0
0.1	10	1	6	0.5	0.5	0
0.01	10	1	6	0.8	0.5	0
0.1	10	1	6	0.8	0.5	0
0.01	10	1	6	1	0.5	0
0.1	10	1	6	1	0.5	0
0.01	20	0.5	2	0.5	0.5	0
0.1	20	0.5	2	0.5	0.5	0
0.25	20	0.5	2	0.5	0.5	0
0.01	20	0.5	2	0.8	0.5	0
0.1	20	0.5	2	0.8	0.5	0
0.25	20	0.5	2	0.8	0.5	0
0.01	20	0.5	2	1	0.5	0
0.1	20	0.5	2	1	0.5	0
0.25	20	0.5	2	1	0.5	0
0.01	20	0.8	2	0.5	0.5	0
0.1	20	0.8	2	0.5	0.5	0
0.25	20	0.8	2	0.5	0.5	0
0.01	20	0.8	2	0.8	0.5	0
0.1	20	0.8	2	0.8	0.5	0
0.25	20	0.8	2	0.8	0.5	0
0.01	20	0.8	2	1	0.5	0
0.1	20	0.8	2	1	0.5	0
0.25	20	0.8	2	1	0.5	0
0.5	20	0.8	2	1	0.5	0
0.01	20	1	2	0.5	0.5	0
0.1	20	1	2	0.5	0.5	0
0.25	20	1	2	0.5	0.5	0
0.01	20	1	2	0.8	0.5	0
0.1	20	1	2	0.8	0.5	0
0.25	20	1	2	0.8	0.5	0
0.01	20	1	2	1	0.5	0
0.1	20	1	2	1	0.5	0
0.25	20	1	2	1	0.5	0
0.01	20	0.5	4	0.5	0.5	0
0.1	20	0.5	4	0.5	0.5	0
0.01	20	0.5	4	0.8	0.5	0
0.1	20	0.5	4	0.8	0.5	0
0.01	20	0.5	4	1	0.5	0
0.1	20	0.5	4	1	0.5	0
0.01	20	0.8	4	0.5	0.5	0
0.1	20	0.8	4	0.5	0.5	0
0.01	20	0.8	4	0.8	0.5	0
0.1	20	0.8	4	0.8	0.5	0
0.01	20	0.8	4	1	0.5	0
0.1	20	0.8	4	1	0.5	0
0.01	20	1	4	0.5	0.5	0
0.1	20	1	4	0.5	0.5	0
0.01	20	1	4	0.8	0.5	0
0.1	20	1	4	0.8	0.5	0
0.01	20	1	4	1	0.5	0
0.1	20	1	4	1	0.5	0
0.01	20	0.5	6	0.5	0.5	0
0.01	20	0.5	6	0.8	0.5	0
0.01	20	0.5	6	1	0.5	0
0.01	20	0.8	6	0.5	0.5	0
0.01	20	0.8	6	0.8	0.5	0
0.01	20	0.8	6	1	0.5	0
0.01	20	1	6	0.5	0.5	0
0.01	20	1	6	0.8	0.5	0
0.01	20	1	6	1	0.5	0
0.01	50	0.5	2	0.5	0.5	0
0.1	50	0.5	2	0.5	0.5	0
0.01	50	0.5	2	0.8	0.5	0
0.1	50	0.5	2	0.8	0.5	0
0.01	50	0.5	2	1	0.5	0
0.1	50	0.5	2	1	0.5	0
0.01	50	0.8	2	0.5	0.5	0
0.1	50	0.8	2	0.5	0.5	0
0.01	50	0.8	2	0.8	0.5	0
0.1	50	0.8	2	0.8	0.5	0
0.01	50	0.8	2	1	0.5	0
0.1	50	0.8	2	1	0.5	0
0.01	50	1	2	0.5	0.5	0
0.1	50	1	2	0.5	0.5	0
0.01	50	1	2	0.8	0.5	0
0.1	50	1	2	0.8	0.5	0
0.01	50	1	2	1	0.5	0
0.1	50	1	2	1	0.5	0
0.01	50	0.5	4	0.5	0.5	0
0.01	50	0.5	4	0.8	0.5	0
0.01	50	0.5	4	1	0.5	0
0.01	50	0.8	4	0.5	0.5	0
0.01	50	0.8	4	0.8	0.5	0
0.01	50	0.8	4	1	0.5	0
0.01	50	1	4	0.5	0.5	0
0.01	50	1	4	0.8	0.5	0
0.01	50	1	4	1	0.5	0
0.01	50	0.5	6	0.5	0.5	0
0.01	50	0.5	6	0.8	0.5	0

0.01	50	0.5	6	1	0.5	0
0.01	50	0.8	6	0.5	0.5	0
0.01	50	0.8	6	0.8	0.5	0
0.01	50	0.8	6	1	0.5	0
0.01	50	1	6	0.5	0.5	0
0.01	50	1	6	0.8	0.5	0
0.01	50	1	6	1	0.5	0

KNN trained on HIV dataset

n_neighbors	weights	leaf_size	Balanced Accuracy	F1 -score
3	distance	30	**0.63**	**0.37**
3	distance	40	0.63	0.37
3	distance	50	0.63	0.37
3	distance	60	0.63	0.37
5	distance	30	0.62	0.35
5	distance	40	0.62	0.35
5	distance	50	0.62	0.35
5	distance	60	0.62	0.35
3	uniform	30	0.62	0.34
3	uniform	40	0.62	0.34
3	uniform	50	0.62	0.34
3	uniform	60	0.62	0.34
7	distance	30	0.61	0.34
7	distance	40	0.61	0.34
7	distance	50	0.61	0.34
7	distance	60	0.61	0.34
5	uniform	30	0.61	0.33
5	uniform	40	0.61	0.33
5	uniform	50	0.61	0.33
5	uniform	60	0.61	0.33
10	distance	30	0.6	0.31
10	distance	40	0.6	0.31
10	distance	50	0.6	0.31
10	distance	60	0.6	0.31
7	uniform	30	0.59	0.29
7	uniform	40	0.59	0.29
7	uniform	50	0.59	0.29
7	uniform	60	0.59	0.29
10	uniform	30	0.56	0.22
10	uniform	40	0.56	0.22
10	uniform	50	0.56	0.22
10	uniform	60	0.56	0.22

Cross validation :

KNN : n_neighbors = 3, weights= distance leaf_size=30

K	Test Accuracy	Test ROC-AUC	Test Balanced_acc	Test MCC	Test Precision	Test Recall	Test F1 score	Test Cohen's Kappa
1	0.91	0.7	0.63	0.33	0.49	0.29	0.36	0.32
2	0.91	0.72	0.64	0.34	0.5	0.3	0.38	0.33
3	0.91	0.72	0.65	0.35	0.49	0.32	0.39	0.34
4	0.91	0.72	0.63	0.33	0.48	0.3	0.37	0.32
5	0.91	0.71	0.64	0.33	0.46	0.31	0.37	0.33
Mean	0.91	0.71	0.64	0.34	0.48	0.3	0.37	0.33
SD	0	0.009	0.008	0.009	0.015	0.011	0.011	0.008
IC	0.90 ± 0	0,71 ± 8.5E-5	0,64 ± 7.5E-5	0,34 ± 8.53E-5	0,48 ± 1.4E-4	0,30 ± 1.0E-4	0,37 ± 1.0E-4	0.33 ± 7.5E-5

MLP trained on HIV dataset

n_hidden_layer	Max_iteration	Activation	solver	alpha	Learning rate	Balanced Accuracy	F1 -score
(120, 80, 40)	120	tanh	sgd	0.01	adaptive	**0.66**	**0.37**
(150, 75, 25)	120	tanh	adam	0.001	constant	0.66	0.37
(120, 80, 40)	50	relu	adam	0.01	constant	0.65	0.37
(120, 80, 40)	120	relu	adam	0.01	adaptive	0.64	0.37
(100, 50, 25)	100	tanh	sgd	0.01	constant	0.66	0.36
(150, 75, 25)	50	relu	adam	0.001	constant	0.66	0.36
(100, 50, 25)	100	relu	adam	0.001	adaptive	0.65	0.36
(120, 80, 40)	50	relu	adam	0.01	adaptive	0.65	0.36
(120, 80, 40)	100	relu	sgd	0.0001	constant	0.65	0.36
(120, 80, 40)	120	relu	adam	0.001	constant	0.65	0.36
(150, 75, 25)	100	relu	sgd	0.0001	constant	0.65	0.36
(100, 50, 25)	50	tanh	adam	0.001	constant	0.64	0.36
(100, 50, 25)	50	relu	adam	0.0001	constant	0.64	0.36
(100, 50, 25)	120	tanh	adam	0.01	constant	0.64	0.36
(120, 80, 40)	50	relu	adam	0.0001	constant	0.64	0.36
(120, 80, 40)	100	tanh	adam	0.001	constant	0.64	0.36
(120, 80, 40)	120	tanh	adam	0.0001	adaptive	0.64	0.36
(120, 80, 40)	120	relu	sgd	0.0001	adaptive	0.64	0.36
(120, 80, 40)	120	relu	adam	0.001	adaptive	0.64	0.36
(120, 80, 40)	120	relu	adam	0.01	constant	0.64	0.36
(150, 75, 25)	100	relu	adam	0.0001	constant	0.64	0.36
(150, 75, 25)	50	relu	adam	0.001	adaptive	0.63	0.36
(100, 50, 25)	50	tanh	adam	0.0001	adaptive	0.67	0.35
(120, 80, 40)	100	tanh	sgd	0.001	constant	0.66	0.35
(150, 75, 25)	120	tanh	adam	0.001	adaptive	0.66	0.35
(100, 50, 25)	50	relu	adam	0.001	constant	0.65	0.35
(100, 50, 25)	120	tanh	adam	0.01	adaptive	0.65	0.35
(100, 50, 25)	120	relu	sgd	0.0001	adaptive	0.65	0.35
(120, 80, 40)	100	relu	adam	0.0001	adaptive	0.65	0.35
(150, 75, 25)	120	tanh	sgd	0.01	constant	0.65	0.35
(150, 75, 25)	120	relu	adam	0.0001	adaptive	0.65	0.35
(100, 50, 25)	100	tanh	adam	0.0001	constant	0.64	0.35
(100, 50, 25)	100	relu	sgd	0.001	adaptive	0.64	0.35
(100, 50, 25)	120	relu	adam	0.0001	adaptive	0.64	0.35
(120, 80, 40)	50	tanh	adam	0.001	constant	0.64	0.35
(120, 80, 40)	50	tanh	adam	0.001	adaptive	0.64	0.35
(120, 80, 40)	100	tanh	adam	0.0001	constant	0.64	0.35
(120, 80, 40)	100	relu	adam	0.01	adaptive	0.64	0.35
(150, 75, 25)	50	tanh	adam	0.01	constant	0.64	0.35
(150, 75, 25)	50	tanh	adam	0.01	adaptive	0.64	0.35
(150, 75, 25)	50	relu	adam	0.01	constant	0.64	0.35
(150, 75, 25)	100	tanh	sgd	0.001	constant	0.64	0.35
(150, 75, 25)	100	tanh	adam	0.001	constant	0.64	0.35
(150, 75, 25)	100	relu	sgd	0.001	adaptive	0.64	0.35
(150, 75, 25)	120	tanh	sgd	0.001	constant	0.64	0.35
(150, 75, 25)	120	tanh	adam	0.01	constant	0.64	0.35
(100, 50, 25)	100	tanh	sgd	0.001	constant	0.63	0.35
(100, 50, 25)	100	relu	sgd	0.0001	constant	0.63	0.35
(100, 50, 25)	120	tanh	adam	0.001	adaptive	0.63	0.35
(100, 50, 25)	120	relu	adam	0.01	constant	0.63	0.35
(150, 75, 25)	50	relu	adam	0.0001	constant	0.63	0.35
(150, 75, 25)	50	relu	adam	0.01	adaptive	0.63	0.35
(150, 75, 25)	100	tanh	sgd	0.0001	adaptive	0.63	0.35
(150, 75, 25)	120	relu	adam	0.01	adaptive	0.63	0.35
(120, 80, 40)	120	tanh	adam	0.0001	constant	0.65	0.34
(100, 50, 25)	50	relu	adam	0.01	constant	0.64	0.34
(100, 50, 25)	100	tanh	sgd	0.0001	adaptive	0.64	0.34
(100, 50, 25)	100	tanh	sgd	0.001	adaptive	0.64	0.34
(120, 80, 40)	120	tanh	sgd	0.001	constant	0.64	0.34
(120, 80, 40)	120	tanh	sgd	0.01	constant	0.64	0.34
(150, 75, 25)	50	tanh	adam	0.0001	adaptive	0.64	0.34
(100, 50, 25)	50	tanh	adam	0.01	constant	0.63	0.34
(100, 50, 25)	100	relu	sgd	0.0001	adaptive	0.63	0.34
(100, 50, 25)	100	relu	sgd	0.01	constant	0.63	0.34
(100, 50, 25)	100	relu	adam	0.001	constant	0.63	0.34
(100, 50, 25)	120	tanh	sgd	0.001	adaptive	0.63	0.34
(100, 50, 25)	120	tanh	sgd	0.01	constant	0.63	0.34
(120, 80, 40)	50	tanh	adam	0.01	constant	0.63	0.34
(120, 80, 40)	100	tanh	sgd	0.001	adaptive	0.63	0.34
(120, 80, 40)	100	tanh	adam	0.001	adaptive	0.63	0.34
(120, 80, 40)	100	tanh	adam	0.01	constant	0.63	0.34
(120, 80, 40)	100	relu	sgd	0.001	constant	0.63	0.34
(120, 80, 40)	100	relu	sgd	0.001	adaptive	0.63	0.34
(120, 80, 40)	100	relu	sgd	0.01	adaptive	0.63	0.34
(120, 80, 40)	100	relu	adam	0.001	adaptive	0.63	0.34
(120, 80, 40)	100	relu	adam	0.01	constant	0.63	0.34
(120, 80, 40)	120	tanh	sgd	0.0001	constant	0.63	0.34
(150, 75, 25)	50	relu	adam	0.0001	adaptive	0.63	0.34
(150, 75, 25)	100	tanh	sgd	0.01	constant	0.63	0.34
(150, 75, 25)	100	tanh	adam	0.0001	adaptive	0.63	0.34
(150, 75, 25)	100	relu	sgd	0.01	adaptive	0.63	0.34
(150, 75, 25)	120	relu	sgd	0.0001	adaptive	0.63	0.34
(100, 50, 25)	50	tanh	adam	0.0001	constant	0.62	0.34
(100, 50, 25)	100	relu	adam	0.0001	constant	0.62	0.34
(100, 50, 25)	100	relu	adam	0.01	adaptive	0.62	0.34
(100, 50, 25)	120	tanh	adam	0.001	constant	0.62	0.34
(100, 50, 25)	120	relu	adam	0.0001	constant	0.62	0.34
(120, 80, 40)	50	relu	adam	0.0001	adaptive	0.62	0.34
(120, 80, 40)	120	relu	sgd	0.01	constant	0.62	0.34
(150, 75, 25)	100	tanh	adam	0.0001	constant	0.62	0.34
(100, 50, 25)	120	tanh	sgd	0.0001	constant	0.67	0.33
(100, 50, 25)	120	tanh	sgd	0.01	adaptive	0.64	0.33
(100, 50, 25)	50	relu	adam	0.01	adaptive	0.63	0.33
(100, 50, 25)	100	tanh	sgd	0.01	adaptive	0.63	0.33
(100, 50, 25)	100	tanh	adam	0.001	adaptive	0.63	0.33
(100, 50, 25)	100	relu	adam	0.0001	adaptive	0.63	0.33
(100, 50, 25)	100	relu	adam	0.01	constant	0.63	0.33
(100, 50, 25)	120	relu	adam	0.001	constant	0.63	0.33
(150, 75, 25)	50	tanh	adam	0.001	constant	0.63	0.33
(150, 75, 25)	100	tanh	sgd	0.0001	constant	0.63	0.33
(100, 50, 25)	100	tanh	adam	0.0001	adaptive	0.62	0.33
(100, 50, 25)	120	relu	sgd	0.0001	constant	0.62	0.33
(100, 50, 25)	120	relu	adam	0.01	adaptive	0.62	0.33
(120, 80, 40)	50	tanh	adam	0.0001	constant	0.62	0.33
(120, 80, 40)	50	tanh	adam	0.0001	adaptive	0.62	0.33
(120, 80, 40)	50	relu	sgd	0.0001	constant	0.62	0.33
(120, 80, 40)	100	tanh	sgd	0.01	constant	0.62	0.33
(120, 80, 40)	120	tanh	adam	0.001	constant	0.62	0.33
(120, 80, 40)	120	tanh	adam	0.01	adaptive	0.62	0.33
(150, 75, 25)	50	tanh	adam	0.0001	constant	0.62	0.33
(150, 75, 25)	100	tanh	adam	0.001	adaptive	0.62	0.33
(150, 75, 25)	100	tanh	adam	0.01	constant	0.62	0.33
(150, 75, 25)	100	relu	adam	0.0001	adaptive	0.62	0.33
(150, 75, 25)	100	relu	adam	0.001	adaptive	0.62	0.33
(150, 75, 25)	100	relu	adam	0.01	constant	0.62	0.33
(150, 75, 25)	120	tanh	sgd	0.0001	adaptive	0.62	0.33
(150, 75, 25)	120	relu	sgd	0.001	adaptive	0.62	0.33
(150, 75, 25)	120	relu	adam	0.0001	constant	0.62	0.33
(150, 75, 25)	120	relu	adam	0.001	constant	0.62	0.33
(150, 75, 25)	120	relu	adam	0.01	constant	0.62	0.33
(100, 50, 25)	50	tanh	adam	0.001	adaptive	0.62	0.32
(100, 50, 25)	50	relu	adam	0.0001	adaptive	0.62	0.32
(100, 50, 25)	100	tanh	adam	0.01	adaptive	0.62	0.32
(100, 50, 25)	120	relu	sgd	0.001	adaptive	0.62	0.32
(150, 75, 25)	100	relu	sgd	0.0001	adaptive	0.62	0.32
(150, 75, 25)	120	relu	sgd	0.001	constant	0.62	0.32
(100, 50, 25)	50	relu	adam	0.001	adaptive	0.61	0.32
(100, 50, 25)	120	tanh	adam	0.0001	constant	0.61	0.32
(100, 50, 25)	120	tanh	adam	0.0001	adaptive	0.61	0.32
(120, 80, 40)	50	relu	adam	0.001	constant	0.61	0.32
(120, 80, 40)	100	relu	sgd	0.01	constant	0.61	0.32
(120, 80, 40)	120	tanh	sgd	0.001	adaptive	0.61	0.32
(120, 80, 40)	120	tanh	adam	0.001	adaptive	0.61	0.32
(120, 80, 40)	120	relu	adam	0.0001	adaptive	0.61	0.32
(150, 75, 25)	120	tanh	sgd	0.0001	constant	0.61	0.32
(150, 75, 25)	120	tanh	sgd	0.01	adaptive	0.61	0.32
(150, 75, 25)	120	relu	sgd	0.01	constant	0.61	0.32

Cross validation :

MLP : hidden_layer_sizes =(120, 80, 40) , max_iterations= 120, activation = tanh, solver = sgd, alpha = 0.01, learning_rate = adaptive

K	Test Accuracy	Test ROC-AUC	est Balanced_ac	Test MCC	Test Precision	Test Recall	Test F1 score	est Cohen's Kappa
1	0.9	0.75	0.62	0.29	0.41	0.28	0.33	0.27
2	0.9	0.75	0.64	0.32	0.44	0.32	0.37	0.32
3	0.89	0.75	0.66	0.33	0.41	0.38	0.39	0.33
4	0.89	0.77	0.65	0.3	0.37	0.36	0.36	0.32
5	0.9	0.75	0.65	0.33	0.42	0.34	0.38	0.33
Mean	0.90	0.75	0.64	0.31	0.41	0.37	0.37	0.31
SD	0.005	0.0089	0.015	0.018	0.025	0.038	0.023	0.025
IC	0.90 ± 4.7E-05	0.75 ± 8.4E-05	0.64 ± 1.4E-4	0.31 ± 1.7E-4	0.41 ± 2.3E-4	0.37 ± 3.6E-4	0.37 ± 2.1E-4	0.31 ± 2.3E-4

(150, 75, 25)	120	relu	adam	0.001	adaptive	0.61	0.32
(100, 50, 25)	120	tanh	sgd	0.0001	adaptive	0.62	0.31
(150, 75, 25)	100	relu	adam	0.01	adaptive	0.62	0.31
(100, 50, 25)	100	tanh	sgd	0.0001	constant	0.61	0.31
(100, 50, 25)	100	relu	sgd	0.01	adaptive	0.61	0.31
(100, 50, 25)	120	relu	sgd	0.01	constant	0.61	0.31
(100, 50, 25)	120	relu	sgd	0.01	adaptive	0.61	0.31
(120, 80, 40)	100	relu	adam	0.0001	constant	0.61	0.31
(120, 80, 40)	100	relu	adam	0.001	constant	0.61	0.31
(120, 80, 40)	120	tanh	adam	0.01	constant	0.61	0.31
(150, 75, 25)	100	tanh	adam	0.01	adaptive	0.61	0.31
(150, 75, 25)	100	relu	sgd	0.001	constant	0.61	0.31
(150, 75, 25)	100	relu	adam	0.001	constant	0.61	0.31
(150, 75, 25)	120	tanh	sgd	0.001	adaptive	0.61	0.31
(150, 75, 25)	120	tanh	adam	0.01	adaptive	0.61	0.31
(150, 75, 25)	120	relu	sgd	0.01	adaptive	0.61	0.31
(120, 80, 40)	50	tanh	sgd	0.0001	constant	0.6	0.31
(120, 80, 40)	50	relu	sgd	0.001	constant	0.6	0.31
(100, 50, 25)	100	relu	sgd	0.001	constant	0.6	0.3
(120, 80, 40)	50	tanh	sgd	0.001	constant	0.6	0.3
(120, 80, 40)	50	relu	sgd	0.001	adaptive	0.6	0.3
(120, 80, 40)	100	tanh	adam	0.0001	adaptive	0.6	0.3
(120, 80, 40)	100	tanh	adam	0.01	adaptive	0.6	0.3
(120, 80, 40)	120	relu	sgd	0.001	adaptive	0.6	0.3
(150, 75, 25)	50	tanh	adam	0.001	adaptive	0.6	0.3
(100, 50, 25)	100	tanh	adam	0.001	constant	0.6	0.29
(120, 80, 40)	50	tanh	sgd	0.0001	adaptive	0.6	0.29
(120, 80, 40)	50	tanh	adam	0.01	adaptive	0.6	0.29
(120, 80, 40)	100	tanh	sgd	0.01	adaptive	0.6	0.29
(120, 80, 40)	120	relu	adam	0.0001	constant	0.6	0.29
(150, 75, 25)	100	tanh	sgd	0.001	adaptive	0.6	0.29
(150, 75, 25)	100	relu	sgd	0.01	constant	0.6	0.29
(100, 50, 25)	50	relu	sgd	0.001	adaptive	0.59	0.29
(100, 50, 25)	100	tanh	adam	0.01	constant	0.59	0.29
(120, 80, 40)	120	tanh	sgd	0.0001	adaptive	0.59	0.29
(150, 75, 25)	120	relu	sgd	0.0001	constant	0.59	0.29
(100, 50, 25)	50	relu	sgd	0.001	constant	0.59	0.28
(100, 50, 25)	50	relu	sgd	0.01	constant	0.59	0.28
(100, 50, 25)	120	relu	adam	0.001	adaptive	0.59	0.28
(120, 80, 40)	50	relu	sgd	0.0001	adaptive	0.59	0.28
(120, 80, 40)	100	relu	sgd	0.0001	adaptive	0.59	0.28
(150, 75, 25)	50	relu	sgd	0.01	adaptive	0.59	0.28
(150, 75, 25)	120	tanh	adam	0.0001	adaptive	0.59	0.28
(120, 80, 40)	120	relu	sgd	0.001	constant	0.59	0.27
(150, 75, 25)	50	relu	sgd	0.001	constant	0.59	0.27
(120, 80, 40)	50	tanh	sgd	0.01	adaptive	0.58	0.27
(150, 75, 25)	50	tanh	sgd	0.01	adaptive	0.58	0.27
(150, 75, 25)	50	relu	sgd	0.001	adaptive	0.58	0.27
(100, 50, 25)	50	tanh	sgd	0.0001	constant	0.58	0.26
(100, 50, 25)	50	tanh	adam	0.01	adaptive	0.58	0.26
(100, 50, 25)	50	relu	sgd	0.01	adaptive	0.58	0.26
(100, 50, 25)	120	tanh	sgd	0.001	constant	0.58	0.26
(120, 80, 40)	50	relu	sgd	0.01	adaptive	0.58	0.26
(120, 80, 40)	100	tanh	sgd	0.0001	constant	0.58	0.26
(150, 75, 25)	50	relu	sgd	0.0001	constant	0.58	0.26
(150, 75, 25)	50	relu	sgd	0.0001	adaptive	0.58	0.26
(150, 75, 25)	120	tanh	adam	0.0001	constant	0.58	0.26
(100, 50, 25)	50	relu	sgd	0.0001	adaptive	0.58	0.25
(100, 50, 25)	120	relu	sgd	0.001	constant	0.58	0.25
(150, 75, 25)	50	tanh	sgd	0.001	constant	0.58	0.25
(150, 75, 25)	50	tanh	sgd	0.01	constant	0.58	0.25
(120, 80, 40)	50	tanh	sgd	0.01	constant	0.57	0.24
(150, 75, 25)	50	tanh	sgd	0.001	adaptive	0.57	0.24
(150, 75, 25)	50	relu	sgd	0.01	constant	0.57	0.24
(150, 75, 25)	100	tanh	sgd	0.01	adaptive	0.57	0.24
(100, 50, 25)	50	relu	sgd	0.0001	constant	0.57	0.23
(120, 80, 40)	50	relu	sgd	0.01	constant	0.57	0.23
(150, 75, 25)	50	tanh	sgd	0.0001	adaptive	0.57	0.23
(100, 50, 25)	50	tanh	sgd	0.001	constant	0.56	0.21
(120, 80, 40)	120	relu	sgd	0.0001	constant	0.56	0.21
(120, 80, 40)	120	relu	sgd	0.01	adaptive	0.56	0.2
(100, 50, 25)	50	tanh	sgd	0.01	adaptive	0.55	0.2
(120, 80, 40)	50	tanh	sgd	0.001	adaptive	0.55	0.2
(100, 50, 25)	50	tanh	sgd	0.0001	adaptive	0.55	0.19
(100, 50, 25)	50	tanh	sgd	0.001	adaptive	0.55	0.19
(120, 80, 40)	100	tanh	sgd	0.0001	adaptive	0.55	0.19
(150, 75, 25)	50	tanh	sgd	0.0001	constant	0.55	0.19
(100, 50, 25)	50	tanh	sgd	0.01	constant	0.55	0.18
(120, 80, 40)	50	relu	adam	0.001	adaptive	0.53	0.13

AFP trained on HIV dataset

Epochs	Dropout	Learning_rate	num_layers	num_timesteps	Batch_size	Balanced Accuracy	F1 -score
60	0.1	0.001	2	2	32	**0.61**	**0.32**
60	0.15	0.001	2	3	16	0.61	0.3
60	0.15	0.001	2	4	16	0.61	0.29
80	0.15	0.001	3	3	16	0.6	0.3
60	0.1	0.001	2	4	32	0.6	0.29
80	0.15	0.001	3	3	32	0.6	0.29
80	0.1	0.001	3	4	32	0.6	0.29
80	0.1	0.001	4	4	16	0.6	0.28
60	0.1	0.001	3	4	32	0.6	0.27
80	0.15	0.001	2	4	16	0.6	0.27
80	0.15	0.001	3	4	32	0.6	0.27
60	0.15	0.001	3	2	32	0.59	0.27
60	0.15	0.001	4	3	32	0.59	0.27
80	0.15	0.001	4	4	16	0.59	0.27
80	0.1	0.001	2	3	16	0.59	0.27
60	0.1	0.001	2	2	16	0.59	0.26
60	0.15	0.001	3	3	32	0.59	0.26
60	0.15	0.001	3	4	32	0.59	0.26
60	0.1	0.001	2	3	32	0.59	0.26
60	0.1	0.001	4	4	32	0.59	0.26
80	0.15	0.001	2	2	32	0.59	0.26
80	0.15	0.001	3	2	32	0.59	0.26
80	0.15	0.001	4	4	32	0.59	0.26
80	0.1	0.001	3	3	32	0.59	0.26
80	0.1	0.001	4	2	32	0.59	0.26
80	0.1	0.001	4	3	32	0.59	0.26
80	0.15	0.001	2	2	16	0.59	0.25
60	0.15	0.001	3	4	16	0.58	0.25
60	0.1	0.001	2	3	16	0.58	0.25
60	0.15	0.001	2	4	32	0.58	0.25
60	0.15	0.001	4	2	32	0.58	0.25
60	0.1	0.001	4	3	32	0.58	0.25
80	0.15	0.001	3	2	16	0.58	0.25
80	0.1	0.001	3	4	16	0.58	0.25
80	0.15	0.001	2	3	32	0.58	0.25
80	0.1	0.001	2	4	32	0.58	0.25
80	0.1	0.001	4	4	32	0.58	0.25
60	0.1	0.001	2	4	16	0.58	0.24
60	0.1	0.001	3	2	16	0.58	0.24
60	0.15	0.001	2	2	32	0.58	0.24
60	0.15	0.001	2	3	32	0.58	0.24
80	0.1	0.001	2	4	16	0.58	0.24
80	0.1	0.001	3	3	16	0.58	0.24
80	0.15	0.001	4	3	32	0.58	0.24
80	0.1	0.001	2	2	32	0.58	0.24
80	0.1	0.001	2	3	32	0.58	0.24
80	0.15	0.001	3	4	16	0.57	0.24
80	0.1	0.001	3	2	32	0.57	0.24
60	0.15	0.001	3	2	16	0.57	0.23
60	0.1	0.001	3	3	16	0.57	0.23
60	0.1	0.001	3	3	32	0.57	0.23
80	0.15	0.001	4	2	16	0.57	0.23
80	0.15	0.001	4	3	16	0.57	0.23
80	0.15	0.001	2	4	32	0.57	0.23
60	0.1	0.001	3	4	16	0.57	0.22
80	0.1	0.001	4	2	16	0.57	0.22
60	0.15	0.001	2	2	16	0.56	0.21
60	0.15	0.001	4	4	32	0.56	0.21
60	0.1	0.001	4	2	32	0.56	0.21
60	0.1	0.001	3	2	32	0.56	0.2
80	0.1	0.001	3	2	16	0.56	0.2
80	0.1	0.001	4	3	16	0.56	0.2
80	0.15	0.001	2	3	16	0.56	0.19
80	0.15	0.001	4	2	32	0.56	0.19
60	0.1	0.001	4	3	16	0.55	0.19
80	0.1	0.001	2	2	16	0.55	0.19
60	0.15	0.001	3	3	16	0.55	0.17
60	0.15	0.001	4	2	16	0.55	0.17
60	0.15	0.001	4	3	16	0.53	0.12
60	0.15	0.001	4	4	16	0.53	0.11
60	0.1	0.001	4	2	16	0.53	0.11
60	0.1	0.001	4	4	16	0.53	0.11

Cross validation :

AFP : Epoch = 60, Dropout = 0.1, Learning rate = 0.001, Batch size = 32, num_layers=2, num_timesteps= 2

K	Test Accuracy	Test ROC-AUC	Test Balanced_acc	Test MCC	Test Precision	Test Recall	Test F1 score	Test Cohen's Kappa
1	0.9	0.77	0.64	0.31	0.4	0.32	0.36	0.3
2	0.89	0.75	0.64	0.32	0.42	0.34	0.37	0.32
3	0.9	0.79	0.63	0.3	0.42	0.3	0.35	0.3
4	0.89	0.77	0.65	0.32	0.39	0.36	0.38	0.32
5	0.9	0.76	0.62	0.3	0.44	0.29	0.35	0.3
Mean	0.90	0.77	0.64	0.31	0.41	0.32	0.36	0.31
SD	0.005	0.015	0.01	0.01	0.02	0.03	0.013	0.011
IC	0.90 ±4.74E-05	0.77 ±0.00014	0.64 ±1.0E-4	0.31 ±9.48E-5	0.41 ±1.8E-4	0.32 ±2.7E-4	0.36 ±1.2E-4	0.31 ±1.0E-4

Chemebrta trained on Malaria dataset

Epochs	Batch_size	warmup_steps	Learning_rate	weight_decay	Balanced Accuracy	F1 -score
5	16	500	0.00005	0.01	**0.8632**	**0.550**
5	16	500	0.00005	0.001	0.8528	0.5032
5	16	500	0.0005	0.01	0.8513	0.5031
5	16	500	0.0005	0.001	0.8624	0.5011
5	16	1000	0.00005	0.01	0.8490	0.5009
5	16	1000	0.00005	0.001	0.8610	0.5007
5	16	1000	0.0005	0.01	0.8522	0.5
5	16	1000	0.0005	0.001	0.862	0.5
5	32	500	0.00005	0.01	0.862	0.5
5	32	500	0.00005	0.001	0.862	0.5
5	32	500	0.0005	0.01	0.862	0.5
5	32	500	0.0005	0.001	0.862	0.5
5	32	1000	0.00005	0.01	0.862	0.5
5	32	1000	0.00005	0.001	0.862	0.5
5	32	1000	0.0005	0.01	0.862	0.5
5	32	1000	0.0005	0.001	0.862	0.5
7	16	500	0.00005	0.01	0.862	0.5
7	16	500	0.00005	0.001	0.862	0.5
7	16	500	0.0005	0.01	0.862	0.5
7	16	500	0.0005	0.001	0.862	0.5
7	16	1000	0.00005	0.01	0.862	0.5
7	16	1000	0.00005	0.001	0.862	0.5
7	16	1000	0.0005	0.01	0.862	0.5
7	16	1000	0.0005	0.001	0.862	0.5
7	32	500	0.00005	0.01	0.862	0.5
7	32	500	0.00005	0.001	0.862	0.5
7	32	500	0.0005	0.01	0.862	0.5
7	32	500	0.0005	0.001	0.862	0.5
7	32	1000	0.00005	0.01	0.862	0.5
7	32	1000	0.00005	0.001	0.862	0.5
7	32	1000	0.0005	0.01	0.862	0.5
7	32	1000	0.0005	0.001	0.862	0.5
10	16	500	0.00005	0.01	0.852	0.5
10	16	500	0.00005	0.001	0.850	0.5
10	16	500	0.0005	0.01	0.86	0.5
10	16	500	0.0005	0.001	0.86	0.5
10	16	1000	0.00005	0.01	0.86	0.5
10	16	1000	0.00005	0.001	0.86	0.5
10	16	1000	0.0005	0.01	0.86	0.5
10	16	1000	0.0005	0.001	0.86	0.49
10	32	500	0.00005	0.01	0.850	0.49
10	32	500	0.00005	0.001	0.850	0.49
10	32	500	0.0005	0.01	0.850	0.49
10	32	500	0.0005	0.001	0.850	0.49
10	32	1000	0.00005	0.01	0.850	0.49
10	32	1000	0.00005	0.001	0.850	0.49
10	32	1000	0.0005	0.01	0.850	0.49
10	32	1000	0.0005	0.001	0.850	0.49

Cross validation :

Chemberta : Epoch = 5, Batch size = 16, warmup_steps = 500, Learning rate = 0.00005, weight_decay = 0.01

K	Test Accuracy	Test ROC-AUC	est Balanced_ac	Test MCC	Test Precision	Test Recall	Test F1 score	est Cohen's Kappa
1	0.93	0.71	0.88	0.50	0.68	0.43	0.53	0.49
2	0.93	0.76	0.89	0.58	0.68	0.55	0.61	0.57
3	0,93	0.73	0.88	0.52	0.64	0.48	0.55	0.51
4	0.93	0.70	0.88	0.50	0.72	0.40	0.51	0.49
5	0.93	0.86	0.74	0.55	0.69	0.5	0.56	0.45
Mean	0.93	0.75	0.86	0.53	0.68	0.47	0.55	0.5
SD	0.003	0.067	0.065	0.031	0.03	0.06	0.04	0.04
IC	0.93 ±2.99E-5	0.75 ±6.6E-4	0.86 ±6.5E-4	0.53 ±3.0E-4	0.68 ±2.9E-4	0.47 ±5.9E-4	0.55 ±3.9E-4	0.5 ±4.2E-4

AFP trained on Malaria dataset

Epochs	Dropout	Learning_rate	num_layers	num_timesteps	Batch_size	Balanced Accuracy	F1 -score
80	0.1	0.001	3	2	32	**0.74**	**0.58**
60	0.15	0.001	3	2	32	0.75	0.57
60	0.1	0.001	3	3	32	0.73	0.56
80	0.15	0.001	4	3	32	0.75	0.55
60	0.1	0.001	2	3	32	0.73	0.55
60	0.1	0.001	2	4	32	0.73	0.55
80	0.15	0.001	2	2	32	0.73	0.55
60	0.15	0.001	3	4	32	0.72	0.55
80	0.15	0.001	2	4	32	0.72	0.55
80	0.1	0.001	2	4	16	0.74	0.54
80	0.1	0.001	3	2	16	0.73	0.54
60	0.15	0.001	3	2	16	0.73	0.54
60	0.1	0.001	4	2	32	0.73	0.54
80	0.15	0.001	3	4	16	0.72	0.54
60	0.15	0.001	2	2	16	0.72	0.54
60	0.1	0.001	2	2	16	0.72	0.54
60	0.15	0.001	2	3	32	0.72	0.54
60	0.15	0.001	4	3	32	0.72	0.54
80	0.15	0.001	4	2	32	0.72	0.54
80	0.1	0.001	2	2	32	0.72	0.54
80	0.1	0.001	4	2	32	0.72	0.54
80	0.1	0.001	4	3	32	0.72	0.54
80	0.15	0.001	3	2	16	0.74	0.53
60	0.1	0.001	2	4	16	0.73	0.53
60	0.15	0.001	2	2	32	0.73	0.53
80	0.15	0.001	2	3	16	0.72	0.53
80	0.1	0.001	2	2	16	0.72	0.53
60	0.15	0.001	3	3	16	0.72	0.53
60	0.1	0.001	4	4	16	0.72	0.53
80	0.15	0.001	2	3	32	0.72	0.53
80	0.1	0.001	4	4	32	0.72	0.53
60	0.1	0.001	3	4	16	0.71	0.53
60	0.1	0.001	3	4	32	0.71	0.53
80	0.15	0.001	3	2	32	0.71	0.53
80	0.15	0.001	4	4	32	0.71	0.53
80	0.15	0.001	2	2	16	0.7	0.53
60	0.15	0.001	2	3	16	0.72	0.52
60	0.1	0.001	4	4	32	0.72	0.52
80	0.1	0.001	2	3	32	0.72	0.52
80	0.15	0.001	3	3	16	0.71	0.52
80	0.1	0.001	3	3	16	0.71	0.52
80	0.1	0.001	4	3	16	0.71	0.52
80	0.1	0.001	4	4	16	0.71	0.52
60	0.15	0.001	2	4	32	0.71	0.52
60	0.15	0.001	3	3	32	0.71	0.52
60	0.1	0.001	2	2	32	0.71	0.52
80	0.15	0.001	3	4	32	0.71	0.52
80	0.1	0.001	3	4	32	0.71	0.52
60	0.15	0.001	4	4	32	0.7	0.52
60	0.1	0.001	2	3	16	0.73	0.51
80	0.1	0.001	2	3	16	0.71	0.51
60	0.1	0.001	3	3	16	0.71	0.51
60	0.1	0.001	4	2	16	0.71	0.51
80	0.15	0.001	4	4	16	0.7	0.51
80	0.15	0.001	3	3	32	0.7	0.51
80	0.1	0.001	2	4	32	0.7	0.51
80	0.1	0.001	3	3	32	0.7	0.51
80	0.15	0.001	4	2	16	0.71	0.5
60	0.15	0.001	2	4	16	0.71	0.5
60	0.15	0.001	4	2	32	0.71	0.5
80	0.1	0.001	4	2	16	0.7	0.5
60	0.1	0.001	4	3	32	0.7	0.5
60	0.1	0.001	4	3	16	0.7	0.49
80	0.15	0.001	2	4	16	0.69	0.49
60	0.15	0.001	4	4	16	0.69	0.49
60	0.1	0.001	3	2	32	0.7	0.48
60	0.15	0.001	3	4	16	0.69	0.48
60	0.1	0.001	3	2	16	0.69	0.48
60	0.15	0.001	4	3	16	0.68	0.48
60	0.15	0.001	4	2	16	0.67	0.47
80	0.1	0.001	3	4	16	0.66	0.44
80	0.15	0.001	4	3	16	0.63	0.38

Cross validation :

AFP : Epoch = 80, Dropout = 0.1 Learning rate = 0.001, Batch size = 32, num_layers=3, num_timesteps= 2

K	Test Accuracy	Test ROC-AUC	Test Balanced_acc	Test MCC	Test Precision	Test Recall	Test F1 score	Test Cohen's Kappa
1	0.93	0.88	0.77	0.57	0.66	0.56	0.61	0.59
2	0.94	0.89	0.78	0.59	0.67	0.58	0.62	0.58
3	0.94	0.88	0.77	0.57	0.65	0.56	0.6	0.54
4	0.93	0.9	0.79	0.59	0.64	0.62	0.63	0.55
5	0.93	0.88	0.78	0.58	0.65	0.6	0.62	0.57
Mean	0.93	0.89	0.78	0.58	0.65	0.58	0.62	0.57
SD	0.005	0.009	0.008	0.01	0.011	0.03	0.01	0.021
IC	0.93 ±4.99E-05	),89 ±8.985E-05	0.78 ±7.9E-5	0.58 ±9.9E-5	0.65 ±1.0E-4	0.58 ±3.0E-4	0.62 ±9.9E-5	0.57 ±2.1E-4

XGBoost trained on Malaria dataset

Learning_rate	n_estimators	subsample	max_depth	colsample_bytree	Balanced Accuracy	F1 -score
0.25	50	1	6	1	**0.73**	**0.6**
0.5	50	0.5	6	1	0.74	0.59
0.5	50	1	6	0.5	0.73	0.59
0.5	50	1	6	0.8	0.73	0.59
0.5	50	1	6	1	0.73	0.59
1	50	1	6	0.5	0.75	0.58
0.5	50	0.5	6	0.5	0.74	0.58
0.5	50	0.8	6	0.5	0.73	0.58
0.25	50	0.8	6	0.8	0.72	0.58
0.25	50	1	6	0.8	0.72	0.58
1	50	1	6	1	0.73	0.57
0.5	50	1	4	0.8	0.72	0.57
0.25	50	0.8	6	1	0.72	0.57
0.25	50	1	6	0.5	0.72	0.57
0.25	50	0.8	6	0.5	0.71	0.57
1	50	1	4	1	0.74	0.56
1	50	1	4	0.5	0.73	0.56
1	50	0.8	6	1	0.73	0.56
0.5	50	0.8	4	1	0.72	0.56
0.5	50	0.5	6	0.8	0.72	0.56
1	50	0.8	6	0.5	0.73	0.55
0.5	50	0.8	6	0.8	0.72	0.55
1	50	1	6	0.8	0.72	0.55
0.25	50	0.5	6	0.8	0.71	0.55
0.5	50	0.8	6	1	0.71	0.55
0.5	20	1	6	0.5	0.7	0.55
1	50	0.8	4	1	0.73	0.54
1	50	0.8	6	0.8	0.73	0.54
0.5	20	0.8	6	0.8	0.7	0.54
0.5	20	1	6	0.8	0.7	0.54
0.5	50	0.5	4	0.8	0.7	0.54
0.5	50	1	4	1	0.7	0.54
0.25	50	0.5	6	0.5	0.7	0.54
1	20	1	6	0.5	0.73	0.53
1	20	0.8	6	1	0.72	0.53
1	20	1	6	0.8	0.72	0.53
1	20	1	6	1	0.72	0.53
1	50	0.8	4	0.8	0.72	0.53
1	50	1	4	0.8	0.71	0.53
0.5	20	0.5	6	0.8	0.7	0.53
0.5	20	0.5	6	1	0.7	0.53
0.5	20	0.8	6	0.5	0.7	0.53
0.5	20	1	6	1	0.7	0.53
0.5	50	0.5	4	0.5	0.7	0.53
0.5	50	0.5	4	1	0.7	0.53
0.25	50	0.5	6	1	0.7	0.53
0.5	50	0.8	4	0.5	0.69	0.53
0.5	50	1	4	0.5	0.69	0.53
1	20	0.8	6	0.8	0.72	0.52
1	10	0.8	6	1	0.71	0.52
1	50	0.8	4	0.5	0.71	0.52
1	50	0.5	6	0.5	0.73	0.51
1	20	0.8	6	0.5	0.71	0.51
0.5	20	0.5	6	0.5	0.7	0.51
0.5	20	0.8	6	1	0.69	0.51
0.5	50	0.8	4	0.8	0.69	0.51
1	20	0.8	4	1	0.7	0.5
1	50	0.5	4	1	0.71	0.49
1	50	0.5	6	1	0.71	0.49
1	8	1	6	0.5	0.69	0.49
1	10	0.8	6	0.5	0.69	0.49
1	10	1	6	0.5	0.69	0.49
1	20	1	4	1	0.69	0.49
0.25	50	0.8	4	0.5	0.67	0.49
1	10	0.8	6	0.8	0.7	0.48
1	10	1	6	1	0.69	0.48
1	20	0.8	4	0.5	0.68	0.48
0.5	8	1	6	0.8	0.67	0.48
1	50	0.5	4	0.5	0.7	0.47
1	50	0.5	6	0.8	0.7	0.47
1	10	1	6	0.8	0.69	0.47
1	8	0.8	6	0.5	0.68	0.47
1	8	0.8	6	0.8	0.68	0.47
1	8	1	6	1	0.68	0.47
1	20	0.8	4	0.8	0.68	0.47
1	20	1	4	0.5	0.67	0.47
1	50	0.8	2	0.5	0.67	0.47
0.5	10	0.5	6	0.8	0.66	0.47
0.5	10	1	6	0.8	0.66	0.47
0.25	20	1	6	1	0.66	0.47
0.25	50	0.5	4	0.5	0.66	0.47
0.25	50	0.8	4	1	0.66	0.47
0.25	50	1	4	0.5	0.66	0.47
0.25	50	1	4	1	0.66	0.47
0.1	50	0.8	6	0.8	0.66	0.47
1	20	0.5	6	0.5	0.69	0.46
1	50	0.5	4	0.8	0.69	0.46
1	8	1	6	0.8	0.68	0.46
1	20	0.5	4	1	0.68	0.46
1	8	0.8	6	1	0.67	0.46
1	10	0.8	4	1	0.67	0.46
1	20	1	4	0.8	0.67	0.46
0.5	10	0.8	6	0.8	0.66	0.46
0.5	10	0.8	6	1	0.66	0.46
0.25	50	0.5	4	1	0.66	0.46
0.25	50	0.8	4	0.8	0.66	0.46
0.1	50	0.8	6	0.5	0.66	0.46
0.25	20	0.8	6	0.5	0.65	0.46
0.25	20	0.8	6	0.8	0.65	0.46
0.25	20	0.8	6	1	0.65	0.46
0.25	50	0.5	4	0.8	0.65	0.46
0.1	50	0.8	6	1	0.65	0.46
0.1	50	1	6	0.5	0.65	0.46
1	20	0.5	6	1	0.7	0.45
0.5	10	0.5	6	1	0.66	0.45
0.5	20	0.5	4	1	0.66	0.45
1	50	0.5	2	0.5	0.66	0.45
1	50	0.8	2	0.8	0.66	0.45
1	50	0.8	2	1	0.66	0.45
1	50	1	2	0.5	0.66	0.45
0.5	8	0.8	6	1	0.65	0.45
0.5	10	1	6	1	0.65	0.45
0.25	20	0.5	6	0.5	0.65	0.45
0.25	20	0.5	6	1	0.65	0.45
0.25	20	1	6	0.5	0.65	0.45
0.25	20	1	6	0.8	0.65	0.45
0.25	50	1	4	0.8	0.65	0.45
0.1	50	0.5	6	0.5	0.65	0.45
0.1	50	0.5	6	1	0.65	0.45
0.1	50	1	6	0.8	0.65	0.45
0.1	50	1	6	1	0.65	0.45
1	20	0.5	6	0.8	0.69	0.44
1	8	0.5	6	0.5	0.68	0.44

Cross validation :

XGBoost : learning_rate = 0,25, n_estimators = 50, subsample= 1, max_depth = 6, colsample_bytree = 1

K	Test Accuracy	Test ROC-AUC	Test Balanced_acc	Test MCC	Test Precision	Test Recall	Test F1 score	Test Cohen's Kappa
1	0.93	0.9	0.73	0.59	0.79	0.48	0.6	0.51
2	0.94	0.9	0.73	0.58	0.81	0.47	0.59	0.56
3	0.94	0.91	0.71	0.56	0.8	0.45	0.57	0.53
4	0.94	0.89	0.71	0.57	0.82	0.47	0.6	0.53
5	0.94	0.89	0.72	0.57	0.78	0.46	0.58	0.51
Mean	0.94	0.9	0.72	0.57	0.8	0.47	0.59	0.53
SD	0.004	0.008	0.01	0.011	0.02	0.011	0.013	0.02
IC	0.94 ±3.99E-05	0.90 ±7.98E-05	0.72 ±9.9E-5	0.57 ±1.1E-4	0.80 ±1.6E-4	0.47 ±1.1E-4	0.59 ±1.3E-4	0.53 ±2.0E-4

1	20	0.5	4	0.5	0.67	0.44
0.5	10	0.5	6	0.5	0.66	0.44
1	50	1	2	1	0.66	0.44
0.5	20	1	4	0.5	0.65	0.44
0.5	20	1	4	0.8	0.65	0.44
0.25	20	0.5	6	0.8	0.65	0.44
0.1	50	0.5	6	0.8	0.65	0.44
1	20	0.5	4	0.8	0.67	0.43
0.5	8	0.5	6	0.5	0.65	0.43
0.5	8	0.5	6	0.8	0.65	0.43
0.5	10	0.8	6	0.5	0.65	0.43
1	50	0.5	2	0.8	0.65	0.43
0.5	8	0.5	6	1	0.64	0.43
0.5	8	1	6	0.5	0.64	0.43
0.5	10	1	6	0.5	0.64	0.43
1	8	0.5	6	1	0.67	0.42
1	10	0.5	6	0.5	0.67	0.42
1	50	1	2	0.8	0.65	0.42
0.5	20	0.5	4	0.8	0.64	0.42
0.5	20	0.8	4	1	0.64	0.42
1	10	0.5	6	0.8	0.67	0.41
1	8	0.5	6	0.8	0.66	0.41
1	10	0.5	6	1	0.66	0.41
0.5	8	0.8	6	0.8	0.64	0.41
0.5	8	1	6	1	0.64	0.41
1	10	0.5	4	0.8	0.64	0.41
0.5	20	1	4	1	0.63	0.41
1	50	0.5	2	1	0.65	0.4
1	8	0.8	4	1	0.64	0.4
1	10	0.8	4	0.5	0.64	0.4
1	10	1	4	0.5	0.64	0.4
0.5	20	0.8	4	0.5	0.63	0.4
1	8	0.5	4	0.5	0.64	0.39
1	8	0.5	4	0.8	0.64	0.39
1	10	0.5	4	1	0.64	0.39
1	8	1	4	1	0.63	0.39
1	10	0.8	4	0.8	0.63	0.39
1	10	1	4	1	0.63	0.39
0.5	20	0.5	4	0.5	0.63	0.39
1	8	0.8	4	0.5	0.63	0.38
1	8	1	4	0.5	0.63	0.38
1	10	0.5	4	0.5	0.63	0.38
1	2	0.8	6	1	0.62	0.38
0.5	8	0.8	6	0.5	0.62	0.38
0.5	20	0.8	4	0.8	0.62	0.38
1	8	0.5	4	1	0.63	0.37
1	10	1	4	0.8	0.63	0.37
1	8	0.8	4	0.8	0.62	0.37
0.5	50	0.5	2	1	0.62	0.36
1	2	0.5	6	1	0.62	0.35
1	2	1	6	0.5	0.62	0.35
1	8	1	4	0.8	0.62	0.35
1	20	0.8	2	1	0.61	0.35
0.5	50	0.5	2	0.8	0.61	0.35
1	2	1	6	1	0.61	0.34
1	20	0.5	2	0.8	0.61	0.34
0.5	50	1	2	0.8	0.61	0.34
0.5	50	1	2	0.5	0.6	0.34
1	2	0.5	6	0.5	0.62	0.33
1	2	0.5	6	0.8	0.61	0.33
1	2	0.8	6	0.5	0.61	0.33
1	2	0.8	6	0.8	0.61	0.33
0.25	10	0.5	6	0.8	0.6	0.33
0.5	50	0.8	2	0.5	0.6	0.33
0.25	10	0.8	6	0.8	0.6	0.32
1	20	0.5	2	0.5	0.6	0.32
0.5	50	0.8	2	1	0.6	0.32
0.5	50	1	2	1	0.6	0.32
1	2	1	6	0.8	0.6	0.31
0.5	10	0.5	4	1	0.6	0.31
1	20	0.8	2	0.8	0.6	0.31
1	20	1	2	0.5	0.6	0.31
1	20	1	2	1	0.6	0.31
0.5	50	0.8	2	0.8	0.6	0.31
0.5	10	0.5	4	0.5	0.59	0.31
0.25	10	1	6	0.8	0.59	0.31
1	20	1	2	0.8	0.59	0.31
1	20	0.5	2	1	0.6	0.3
0.5	10	0.8	4	0.5	0.59	0.3
0.25	10	0.5	6	0.5	0.59	0.3
0.25	10	0.5	6	1	0.59	0.3
0.25	10	0.8	6	0.5	0.59	0.3
1	20	0.8	2	0.5	0.59	0.3
0.5	50	0.5	2	0.5	0.59	0.3
0.5	10	0.8	4	1	0.59	0.29
0.25	10	1	6	1	0.59	0.29
0.25	20	0.8	4	1	0.59	0.29
0.5	10	0.5	4	0.8	0.58	0.28
0.5	10	1	4	1	0.58	0.28
0.25	10	0.8	6	1	0.58	0.28
0.25	10	1	6	0.5	0.58	0.28
0.25	8	0.5	6	0.8	0.58	0.27
0.25	8	0.8	6	0.8	0.58	0.27
0.5	10	0.8	4	0.8	0.58	0.27
0.5	10	1	4	0.8	0.58	0.27
0.25	20	0.5	4	0.8	0.58	0.27
0.25	20	0.8	4	0.8	0.58	0.27
0.25	20	1	4	1	0.58	0.27
0.25	8	0.5	6	0.5	0.58	0.26
0.25	8	1	6	1	0.58	0.26
0.5	10	1	4	0.5	0.58	0.26
0.25	8	0.5	6	1	0.57	0.26
0.25	20	0.5	4	0.5	0.57	0.26
0.25	20	0.8	4	0.5	0.57	0.26
0.5	8	0.5	4	0.8	0.57	0.25
0.25	8	0.8	6	1	0.57	0.25
0.25	20	0.5	4	1	0.57	0.25
0.5	8	1	4	0.8	0.57	0.24
0.25	8	1	6	0.5	0.57	0.24
0.25	8	1	6	0.8	0.57	0.24
0.1	50	0.5	4	0.5	0.57	0.24
0.1	50	0.5	4	0.8	0.57	0.24
0.1	50	0.5	4	1	0.57	0.24
0.1	50	1	4	0.8	0.57	0.24
1	2	0.5	4	0.5	0.57	0.23
1	2	0.5	4	1	0.57	0.23
0.5	8	0.8	4	0.5	0.57	0.23
0.25	8	0.8	6	0.5	0.57	0.23
1	10	0.5	2	0.5	0.57	0.23
1	10	0.8	2	0.5	0.57	0.23
0.1	20	1	6	1	0.57	0.23
0.5	8	0.5	4	0.5	0.56	0.23
0.5	8	1	4	0.5	0.56	0.23
0.25	20	1	4	0.8	0.56	0.23
0.1	20	0.5	6	0.8	0.56	0.23
0.1	50	0.8	4	0.8	0.56	0.23
1	2	0.5	4	0.8	0.56	0.22

1	2	0.8	4	0.5	0.56	0.22
0.5	8	0.8	4	0.8	0.56	0.22
0.5	8	0.8	4	1	0.56	0.22
0.5	8	1	4	1	0.56	0.22
1	10	0.5	2	1	0.56	0.22
1	10	0.8	2	1	0.56	0.22
0.1	20	0.5	6	1	0.56	0.22
0.1	20	1	6	0.8	0.56	0.22
0.1	50	1	4	0.5	0.56	0.22
1	2	1	4	0.5	0.56	0.21
0.5	2	0.5	6	1	0.56	0.21
0.5	2	1	6	0.8	0.56	0.21
1	8	0.8	2	1	0.56	0.21
0.5	8	0.5	4	1	0.56	0.21
1	10	0.5	2	0.8	0.56	0.21
1	10	1	2	0.8	0.56	0.21
0.1	20	0.5	6	0.5	0.56	0.21
0.1	20	0.8	6	0.8	0.56	0.21
0.1	20	0.8	6	1	0.56	0.21
0.1	50	0.8	4	0.5	0.56	0.21
0.1	50	0.8	4	1	0.56	0.21
1	2	0.8	4	0.8	0.56	0.2
0.5	2	0.8	6	0.8	0.56	0.2
1	8	0.5	2	0.5	0.56	0.2
1	8	1	2	0.8	0.56	0.2
0.5	20	0.5	2	0.8	0.56	0.2
0.25	20	1	4	0.5	0.56	0.2
0.1	50	1	4	1	0.56	0.2
1	2	0.8	4	1	0.55	0.2
1	2	1	4	1	0.55	0.19
1	8	0.5	2	0.8	0.55	0.19
1	8	0.5	2	1	0.55	0.19
1	10	0.8	2	0.8	0.55	0.19
1	10	1	2	0.5	0.55	0.19
0.1	20	0.8	6	0.5	0.55	0.19
0.1	20	1	6	0.5	0.55	0.19
0.5	2	0.8	6	1	0.55	0.18
0.5	2	1	6	1	0.55	0.18
1	8	1	2	0.5	0.55	0.18
1	10	1	2	1	0.55	0.18
0.25	50	0.8	2	0.5	0.55	0.18
0.5	2	0.5	6	0.8	0.55	0.17
0.5	20	0.5	2	1	0.55	0.17
0.25	50	0.5	2	0.8	0.55	0.17
0.5	2	0.5	6	0.5	0.54	0.16
1	8	1	2	1	0.54	0.16
0.25	10	0.5	4	0.8	0.54	0.16
0.5	20	1	2	0.8	0.54	0.16
0.25	50	0.5	2	0.5	0.54	0.16
0.25	50	0.5	2	1	0.54	0.16
0.25	50	1	2	1	0.54	0.16
1	2	1	4	0.8	0.54	0.15
1	8	0.8	2	0.5	0.54	0.15
0.5	20	0.5	2	0.5	0.54	0.15
0.5	20	0.8	2	0.5	0.54	0.15
0.5	20	0.8	2	0.8	0.54	0.15
0.5	20	1	2	0.5	0.54	0.15
0.5	20	1	2	1	0.54	0.15
0.25	50	0.8	2	0.8	0.54	0.15
0.25	50	0.8	2	1	0.54	0.15
0.25	50	1	2	0.5	0.54	0.15
0.5	2	0.8	6	0.5	0.54	0.14
1	8	0.8	2	0.8	0.54	0.14
0.5	20	0.8	2	1	0.54	0.14
0.25	50	1	2	0.8	0.54	0.14
0.25	10	0.5	4	1	0.54	0.13
0.5	2	1	6	0.5	0.53	0.13
0.25	10	0.8	4	1	0.53	0.13
0.25	10	1	4	1	0.53	0.12
1	2	0.5	2	0.5	0.53	0.11
1	2	0.5	2	0.8	0.53	0.11
1	2	0.5	2	1	0.53	0.11
1	2	0.8	2	1	0.53	0.11
0.25	8	0.5	4	0.5	0.53	0.11
0.25	10	0.5	4	0.5	0.53	0.11
0.25	10	0.8	4	0.5	0.53	0.11
0.25	8	0.5	4	0.8	0.53	0.1
0.25	8	0.5	4	1	0.53	0.1
0.25	10	1	4	0.8	0.53	0.1
1	2	1	2	1	0.52	0.09
0.25	8	0.8	4	1	0.52	0.09
0.25	8	1	4	0.8	0.52	0.09
0.25	10	0.8	4	0.8	0.52	0.09
0.25	10	1	4	0.5	0.52	0.09
1	2	1	2	0.8	0.52	0.08
0.25	8	1	4	1	0.52	0.08
1	2	0.8	2	0.8	0.52	0.07
0.5	2	0.5	4	0.5	0.52	0.07
0.25	8	0.8	4	0.5	0.52	0.07
0.25	8	0.8	4	0.8	0.52	0.07
0.25	8	1	4	0.5	0.52	0.07
0.5	10	0.5	2	1	0.52	0.07
0.5	2	0.5	4	0.8	0.52	0.06
0.1	10	0.5	6	0.8	0.52	0.06
0.1	20	0.5	4	0.5	0.52	0.06
0.1	20	0.5	4	0.8	0.52	0.06
0.1	20	1	4	0.5	0.52	0.06
0.1	20	1	4	1	0.52	0.06
0.5	2	0.5	4	1	0.51	0.06
0.5	2	0.8	4	0.8	0.51	0.06
0.5	8	0.5	2	1	0.51	0.06
0.1	10	0.5	6	0.5	0.51	0.06
0.1	10	0.8	6	0.8	0.51	0.06
0.1	10	0.8	6	1	0.51	0.06
0.1	10	1	6	1	0.51	0.06
0.1	20	0.5	4	1	0.51	0.06
0.1	20	1	4	0.8	0.51	0.06
0.5	2	0.8	4	1	0.51	0.05
0.5	10	0.5	2	0.8	0.51	0.05
0.1	10	0.5	6	1	0.51	0.05
0.1	10	0.8	6	0.5	0.51	0.05
0.1	10	1	6	0.8	0.51	0.05
0.1	20	0.8	4	0.5	0.51	0.05
0.1	20	0.8	4	0.8	0.51	0.05
0.1	20	0.8	4	1	0.51	0.05
0.5	2	1	4	0.8	0.51	0.04
0.5	2	1	4	1	0.51	0.04
0.1	8	0.5	6	0.5	0.51	0.04
0.1	8	1	6	0.5	0.51	0.04
0.1	8	1	6	0.8	0.51	0.04
0.5	10	0.5	2	0.5	0.51	0.04
0.1	10	1	6	0.5	0.51	0.04
0.25	20	0.5	2	1	0.51	0.04
0.25	20	1	2	1	0.51	0.04
0.5	2	0.8	4	0.5	0.51	0.03
0.25	2	0.8	6	0.8	0.51	0.03
0.5	8	0.5	2	0.5	0.51	0.03

0.5	8	0.5	2	0.8	0.51	0.03
0.1	8	0.5	6	0.8	0.51	0.03
0.1	8	0.5	6	1	0.51	0.03
0.1	8	0.8	6	0.5	0.51	0.03
0.1	8	0.8	6	0.8	0.51	0.03
0.1	8	0.8	6	1	0.51	0.03
0.5	10	0.8	2	0.8	0.51	0.03
0.5	10	0.8	2	1	0.51	0.03
0.5	10	1	2	0.8	0.51	0.03
0.5	10	1	2	1	0.51	0.03
0.25	20	0.5	2	0.5	0.51	0.03
0.25	2	0.5	6	0.5	0.51	0.02
0.25	2	1	6	0.8	0.51	0.02
0.25	20	0.5	2	0.8	0.51	0.02
0.25	20	0.8	2	0.5	0.51	0.02
0.25	20	1	2	0.8	0.51	0.02
0.1	50	0.5	2	0.5	0.51	0.02
0.1	50	0.5	2	0.8	0.51	0.02
0.1	50	0.5	2	1	0.51	0.02
0.25	2	0.8	6	0.5	0.5	0.02
0.5	8	0.8	2	0.8	0.5	0.02
0.5	8	1	2	1	0.5	0.02
0.1	8	1	6	1	0.5	0.02
0.5	10	0.8	2	0.5	0.5	0.02
0.5	10	1	2	0.5	0.5	0.02
0.25	20	0.8	2	1	0.5	0.02
0.25	20	1	2	0.5	0.5	0.02
0.1	50	0.8	2	0.5	0.5	0.02
0.1	50	0.8	2	0.8	0.5	0.02
0.1	50	1	2	1	0.5	0.02
0.5	2	1	4	0.5	0.5	0.01
0.25	2	0.5	6	1	0.5	0.01
0.25	2	1	6	0.5	0.5	0.01
0.5	8	0.8	2	0.5	0.5	0.01
0.5	8	0.8	2	1	0.5	0.01
0.5	8	1	2	0.5	0.5	0.01
0.5	8	1	2	0.8	0.5	0.01
0.1	10	0.5	4	0.5	0.5	0.01
0.1	10	1	4	0.8	0.5	0.01
0.25	20	0.8	2	0.8	0.5	0.01
0.1	50	0.8	2	1	0.5	0.01
0.1	50	1	2	0.5	0.5	0.01
0.1	50	1	2	0.8	0.5	0.01
0.01	2	0.5	2	0.5	0.5	0
0.1	2	0.5	2	0.5	0.5	0
0.25	2	0.5	2	0.5	0.5	0
0.5	2	0.5	2	0.5	0.5	0
0.01	2	0.5	2	0.8	0.5	0
0.1	2	0.5	2	0.8	0.5	0
0.25	2	0.5	2	0.8	0.5	0
0.5	2	0.5	2	0.8	0.5	0
0.01	2	0.5	2	1	0.5	0
0.1	2	0.5	2	1	0.5	0
0.25	2	0.5	2	1	0.5	0
0.5	2	0.5	2	1	0.5	0
0.01	2	0.8	2	0.5	0.5	0
0.1	2	0.8	2	0.5	0.5	0
0.25	2	0.8	2	0.5	0.5	0
0.5	2	0.8	2	0.5	0.5	0
1	2	0.8	2	0.5	0.5	0
0.01	2	0.8	2	0.8	0.5	0
0.1	2	0.8	2	0.8	0.5	0
0.25	2	0.8	2	0.8	0.5	0
0.5	2	0.8	2	0.8	0.5	0
0.01	2	0.8	2	1	0.5	0
0.1	2	0.8	2	1	0.5	0
0.25	2	0.8	2	1	0.5	0
0.5	2	0.8	2	1	0.5	0
0.01	2	1	2	0.5	0.5	0
0.1	2	1	2	0.5	0.5	0
0.25	2	1	2	0.5	0.5	0
0.5	2	1	2	0.5	0.5	0
1	2	1	2	0.5	0.5	0
0.01	2	1	2	0.8	0.5	0
0.1	2	1	2	0.8	0.5	0
0.25	2	1	2	0.8	0.5	0
0.5	2	1	2	0.8	0.5	0
0.01	2	1	2	1	0.5	0
0.1	2	1	2	1	0.5	0
0.25	2	1	2	1	0.5	0
0.5	2	1	2	1	0.5	0
0.01	2	0.5	4	0.5	0.5	0
0.1	2	0.5	4	0.5	0.5	0
0.25	2	0.5	4	0.5	0.5	0
0.01	2	0.5	4	0.8	0.5	0
0.1	2	0.5	4	0.8	0.5	0
0.25	2	0.5	4	0.8	0.5	0
0.01	2	0.5	4	1	0.5	0
0.1	2	0.5	4	1	0.5	0
0.25	2	0.5	4	1	0.5	0
0.01	2	0.8	4	0.5	0.5	0
0.1	2	0.8	4	0.5	0.5	0
0.25	2	0.8	4	0.5	0.5	0
0.01	2	0.8	4	0.8	0.5	0
0.1	2	0.8	4	0.8	0.5	0
0.25	2	0.8	4	0.8	0.5	0
0.01	2	0.8	4	1	0.5	0
0.1	2	0.8	4	1	0.5	0
0.25	2	0.8	4	1	0.5	0
0.01	2	1	4	0.5	0.5	0
0.1	2	1	4	0.5	0.5	0
0.25	2	1	4	0.5	0.5	0
0.01	2	1	4	0.8	0.5	0
0.1	2	1	4	0.8	0.5	0
0.25	2	1	4	0.8	0.5	0
0.01	2	1	4	1	0.5	0
0.1	2	1	4	1	0.5	0
0.25	2	1	4	1	0.5	0
0.01	2	0.5	6	0.5	0.5	0
0.1	2	0.5	6	0.5	0.5	0
0.01	2	0.5	6	0.8	0.5	0
0.1	2	0.5	6	0.8	0.5	0
0.25	2	0.5	6	0.8	0.5	0
0.01	2	0.5	6	1	0.5	0
0.1	2	0.5	6	1	0.5	0
0.01	2	0.8	6	0.5	0.5	0
0.1	2	0.8	6	0.5	0.5	0
0.01	2	0.8	6	0.8	0.5	0
0.1	2	0.8	6	0.8	0.5	0
0.01	2	0.8	6	1	0.5	0
0.1	2	0.8	6	1	0.5	0
0.25	2	0.8	6	1	0.5	0
0.01	2	1	6	0.5	0.5	0
0.1	2	1	6	0.5	0.5	0
0.01	2	1	6	0.8	0.5	0
0.1	2	1	6	0.8	0.5	0
0.01	2	1	6	1	0.5	0

0.1	2	1	6	1	0.5	0
0.25	2	1	6	1	0.5	0
0.01	8	0.5	2	0.5	0.5	0
0.1	8	0.5	2	0.5	0.5	0
0.25	8	0.5	2	0.5	0.5	0
0.01	8	0.5	2	0.8	0.5	0
0.1	8	0.5	2	0.8	0.5	0
0.25	8	0.5	2	0.8	0.5	0
0.01	8	0.5	2	1	0.5	0
0.1	8	0.5	2	1	0.5	0
0.25	8	0.5	2	1	0.5	0
0.01	8	0.8	2	0.5	0.5	0
0.1	8	0.8	2	0.5	0.5	0
0.25	8	0.8	2	0.5	0.5	0
0.01	8	0.8	2	0.8	0.5	0
0.1	8	0.8	2	0.8	0.5	0
0.25	8	0.8	2	0.8	0.5	0
0.01	8	0.8	2	1	0.5	0
0.1	8	0.8	2	1	0.5	0
0.25	8	0.8	2	1	0.5	0
0.01	8	1	2	0.5	0.5	0
0.1	8	1	2	0.5	0.5	0
0.25	8	1	2	0.5	0.5	0
0.01	8	1	2	0.8	0.5	0
0.1	8	1	2	0.8	0.5	0
0.25	8	1	2	0.8	0.5	0
0.01	8	1	2	1	0.5	0
0.1	8	1	2	1	0.5	0
0.25	8	1	2	1	0.5	0
0.01	8	0.5	4	0.5	0.5	0
0.1	8	0.5	4	0.5	0.5	0
0.01	8	0.5	4	0.8	0.5	0
0.1	8	0.5	4	0.8	0.5	0
0.01	8	0.5	4	1	0.5	0
0.1	8	0.5	4	1	0.5	0
0.01	8	0.8	4	0.5	0.5	0
0.1	8	0.8	4	0.5	0.5	0
0.01	8	0.8	4	0.8	0.5	0
0.1	8	0.8	4	0.8	0.5	0
0.01	8	0.8	4	1	0.5	0
0.1	8	0.8	4	1	0.5	0
0.01	8	1	4	0.5	0.5	0
0.1	8	1	4	0.5	0.5	0
0.01	8	1	4	0.8	0.5	0
0.1	8	1	4	0.8	0.5	0
0.01	8	1	4	1	0.5	0
0.1	8	1	4	1	0.5	0
0.01	8	0.5	6	0.5	0.5	0
0.01	8	0.5	6	0.8	0.5	0
0.01	8	0.5	6	1	0.5	0
0.01	8	0.8	6	0.5	0.5	0
0.01	8	0.8	6	0.8	0.5	0
0.01	8	0.8	6	1	0.5	0
0.01	8	1	6	0.5	0.5	0
0.01	8	1	6	0.8	0.5	0
0.01	8	1	6	1	0.5	0
0.01	10	0.5	2	0.5	0.5	0
0.1	10	0.5	2	0.5	0.5	0
0.25	10	0.5	2	0.5	0.5	0
0.01	10	0.5	2	0.8	0.5	0
0.1	10	0.5	2	0.8	0.5	0
0.25	10	0.5	2	0.8	0.5	0
0.01	10	0.5	2	1	0.5	0
0.1	10	0.5	2	1	0.5	0
0.25	10	0.5	2	1	0.5	0
0.01	10	0.8	2	0.5	0.5	0
0.1	10	0.8	2	0.5	0.5	0
0.25	10	0.8	2	0.5	0.5	0
0.01	10	0.8	2	0.8	0.5	0
0.1	10	0.8	2	0.8	0.5	0
0.25	10	0.8	2	0.8	0.5	0
0.01	10	0.8	2	1	0.5	0
0.1	10	0.8	2	1	0.5	0
0.25	10	0.8	2	1	0.5	0
0.01	10	1	2	0.5	0.5	0
0.1	10	1	2	0.5	0.5	0
0.25	10	1	2	0.5	0.5	0
0.01	10	1	2	0.8	0.5	0
0.1	10	1	2	0.8	0.5	0
0.25	10	1	2	0.8	0.5	0
0.01	10	1	2	1	0.5	0
0.1	10	1	2	1	0.5	0
0.25	10	1	2	1	0.5	0
0.01	10	0.5	4	0.5	0.5	0
0.01	10	0.5	4	0.8	0.5	0
0.1	10	0.5	4	0.8	0.5	0
0.01	10	0.5	4	1	0.5	0
0.1	10	0.5	4	1	0.5	0
0.01	10	0.8	4	0.5	0.5	0
0.1	10	0.8	4	0.5	0.5	0
0.01	10	0.8	4	0.8	0.5	0
0.1	10	0.8	4	0.8	0.5	0
0.01	10	0.8	4	1	0.5	0
0.1	10	0.8	4	1	0.5	0
0.01	10	1	4	0.5	0.5	0
0.1	10	1	4	0.5	0.5	0
0.01	10	1	4	0.8	0.5	0
0.01	10	1	4	1	0.5	0
0.1	10	1	4	1	0.5	0
0.01	10	0.5	6	0.5	0.5	0
0.01	10	0.5	6	0.8	0.5	0
0.01	10	0.5	6	1	0.5	0
0.01	10	0.8	6	0.5	0.5	0
0.01	10	0.8	6	0.8	0.5	0
0.01	10	0.8	6	1	0.5	0
0.01	10	1	6	0.5	0.5	0
0.01	10	1	6	0.8	0.5	0
0.01	10	1	6	1	0.5	0
0.01	20	0.5	2	0.5	0.5	0
0.1	20	0.5	2	0.5	0.5	0
0.01	20	0.5	2	0.8	0.5	0
0.1	20	0.5	2	0.8	0.5	0
0.01	20	0.5	2	1	0.5	0
0.1	20	0.5	2	1	0.5	0
0.01	20	0.8	2	0.5	0.5	0
0.1	20	0.8	2	0.5	0.5	0
0.01	20	0.8	2	0.8	0.5	0
0.1	20	0.8	2	0.8	0.5	0
0.01	20	0.8	2	1	0.5	0
0.1	20	0.8	2	1	0.5	0
0.01	20	1	2	0.5	0.5	0
0.1	20	1	2	0.5	0.5	0
0.01	20	1	2	0.8	0.5	0
0.1	20	1	2	0.8	0.5	0
0.01	20	1	2	1	0.5	0
0.1	20	1	2	1	0.5	0
0.01	20	0.5	4	0.5	0.5	0

0.01	20	0.5	4	0.8	0.5	0
0.01	20	0.5	4	1	0.5	0
0.01	20	0.8	4	0.5	0.5	0
0.01	20	0.8	4	0.8	0.5	0
0.01	20	0.8	4	1	0.5	0
0.01	20	1	4	0.5	0.5	0
0.01	20	1	4	0.8	0.5	0
0.01	20	1	4	1	0.5	0
0.01	20	0.5	6	0.5	0.5	0
0.01	20	0.5	6	0.8	0.5	0
0.01	20	0.5	6	1	0.5	0
0.01	20	0.8	6	0.5	0.5	0
0.01	20	0.8	6	0.8	0.5	0
0.01	20	0.8	6	1	0.5	0
0.01	20	1	6	0.5	0.5	0
0.01	20	1	6	0.8	0.5	0
0.01	20	1	6	1	0.5	0
0.01	50	0.5	2	0.5	0.5	0
0.01	50	0.5	2	0.8	0.5	0
0.01	50	0.5	2	1	0.5	0
0.01	50	0.8	2	0.5	0.5	0
0.01	50	0.8	2	0.8	0.5	0
0.01	50	0.8	2	1	0.5	0
0.01	50	1	2	0.5	0.5	0
0.01	50	1	2	0.8	0.5	0
0.01	50	1	2	1	0.5	0
0.01	50	0.5	4	0.5	0.5	0
0.01	50	0.5	4	0.8	0.5	0
0.01	50	0.5	4	1	0.5	0
0.01	50	0.8	4	0.5	0.5	0
0.01	50	0.8	4	0.8	0.5	0
0.01	50	0.8	4	1	0.5	0
0.01	50	1	4	0.5	0.5	0
0.01	50	1	4	0.8	0.5	0
0.01	50	1	4	1	0.5	0
0.01	50	0.5	6	0.5	0.5	0
0.01	50	0.5	6	0.8	0.5	0
0.01	50	0.5	6	1	0.5	0
0.01	50	0.8	6	0.5	0.5	0
0.01	50	0.8	6	0.8	0.5	0
0.01	50	0.8	6	1	0.5	0
0.01	50	1	6	0.5	0.5	0
0.01	50	1	6	0.8	0.5	0
0.01	50	1	6	1	0.5	0

MLP trained on Malaria dataset

n_hidden_layer	Max_iteration	Activation	solver	alpha	Learning rate	Balanced Accuracy	F1 -score
(100, 50, 25)	50	tanh	adam	0.001	constant	**0.78**	**0.62**
(100, 50, 25)	50	tanh	adam	0.001	adaptive	0.78	0.61
(100, 50, 25)	50	relu	sgd	0.001	constant	0.77	0.61
(100, 50, 25)	50	relu	sgd	0.001	adaptive	0.77	0.61
(100, 50, 25)	50	relu	sgd	0.01	constant	0.77	0.61
(100, 50, 25)	50	relu	sgd	0.01	adaptive	0.77	0.61
(100, 50, 25)	50	relu	adam	0.0001	constant	0.77	0.61
(100, 50, 25)	50	tanh	sgd	0.001	constant	0.79	0.6
(100, 50, 25)	50	tanh	adam	0.01	constant	0.78	0.6
(100, 50, 25)	50	relu	adam	0.0001	adaptive	0.77	0.6
(100, 50, 25)	50	relu	adam	0.001	constant	0.77	0.6
(100, 50, 25)	50	relu	adam	0.001	adaptive	0.77	0.6
(100, 50, 25)	50	relu	adam	0.01	constant	0.77	0.6
(100, 50, 25)	50	relu	adam	0.01	adaptive	0.77	0.6
(100, 50, 25)	100	tanh	sgd	0.0001	constant	0.77	0.6
(100, 50, 25)	100	tanh	sgd	0.0001	adaptive	0.77	0.6
(100, 50, 25)	100	tanh	sgd	0.001	constant	0.77	0.6
(100, 50, 25)	100	tanh	sgd	0.001	adaptive	0.77	0.6
(100, 50, 25)	100	tanh	sgd	0.01	constant	0.77	0.6
(100, 50, 25)	100	tanh	sgd	0.01	adaptive	0.77	0.6
(100, 50, 25)	100	tanh	adam	0.0001	constant	0.77	0.6
(100, 50, 25)	100	tanh	adam	0.0001	adaptive	0.77	0.6
(100, 50, 25)	100	tanh	adam	0.001	constant	0.77	0.6
(100, 50, 25)	100	tanh	adam	0.001	adaptive	0.77	0.6
(100, 50, 25)	100	tanh	adam	0.01	constant	0.77	0.6
(100, 50, 25)	100	tanh	adam	0.01	adaptive	0.77	0.6
(100, 50, 25)	120	tanh	adam	0.01	adaptive	0.76	0.6
(100, 50, 25)	120	relu	sgd	0.0001	constant	0.76	0.6
(100, 50, 25)	120	relu	sgd	0.0001	adaptive	0.76	0.6
(100, 50, 25)	120	relu	sgd	0.001	constant	0.76	0.6
(100, 50, 25)	120	relu	sgd	0.001	adaptive	0.76	0.6
(120, 80, 40)	100	tanh	adam	0.001	adaptive	0.75	0.6
(120, 80, 40)	100	tanh	adam	0.01	constant	0.75	0.6
(120, 80, 40)	100	tanh	adam	0.01	adaptive	0.75	0.6
(120, 80, 40)	100	relu	sgd	0.0001	constant	0.75	0.6
(120, 80, 40)	100	relu	sgd	0.0001	adaptive	0.75	0.6
(100, 50, 25)	50	tanh	sgd	0.0001	constant	0.8	0.59
(100, 50, 25)	50	tanh	sgd	0.001	adaptive	0.79	0.59
(100, 50, 25)	50	tanh	sgd	0.01	constant	0.79	0.59
(100, 50, 25)	50	tanh	sgd	0.01	adaptive	0.79	0.59
(100, 50, 25)	50	tanh	adam	0.01	adaptive	0.78	0.59
(100, 50, 25)	50	relu	sgd	0.0001	constant	0.78	0.59
(100, 50, 25)	100	relu	sgd	0.0001	constant	0.77	0.59
(100, 50, 25)	100	relu	sgd	0.0001	adaptive	0.77	0.59
(100, 50, 25)	100	relu	sgd	0.001	constant	0.77	0.59
(100, 50, 25)	100	relu	sgd	0.001	adaptive	0.77	0.59
(100, 50, 25)	100	relu	sgd	0.01	constant	0.77	0.59
(100, 50, 25)	100	relu	sgd	0.01	adaptive	0.77	0.59
(100, 50, 25)	100	relu	adam	0.0001	constant	0.77	0.59
(100, 50, 25)	100	relu	adam	0.0001	adaptive	0.77	0.59
(100, 50, 25)	100	relu	adam	0.001	constant	0.77	0.59
(100, 50, 25)	120	relu	sgd	0.01	constant	0.76	0.59
(100, 50, 25)	120	relu	sgd	0.01	adaptive	0.76	0.59
(100, 50, 25)	120	relu	adam	0.0001	constant	0.76	0.59
(100, 50, 25)	120	relu	adam	0.0001	adaptive	0.76	0.59
(100, 50, 25)	120	relu	adam	0.001	constant	0.76	0.59
(100, 50, 25)	120	relu	adam	0.001	adaptive	0.76	0.59
(100, 50, 25)	120	relu	adam	0.01	constant	0.76	0.59
(100, 50, 25)	120	relu	adam	0.01	adaptive	0.76	0.59
(120, 80, 40)	50	tanh	sgd	0.0001	constant	0.76	0.59
(120, 80, 40)	50	tanh	sgd	0.0001	adaptive	0.76	0.59
(120, 80, 40)	50	tanh	sgd	0.001	constant	0.76	0.59
(120, 80, 40)	50	tanh	sgd	0.001	adaptive	0.76	0.59
(120, 80, 40)	50	tanh	sgd	0.01	constant	0.76	0.59
(120, 80, 40)	50	tanh	sgd	0.01	adaptive	0.76	0.59
(120, 80, 40)	50	tanh	adam	0.0001	constant	0.76	0.59
(120, 80, 40)	50	tanh	adam	0.0001	adaptive	0.76	0.59
(120, 80, 40)	50	tanh	adam	0.001	constant	0.76	0.59
(120, 80, 40)	50	tanh	adam	0.001	adaptive	0.76	0.59
(120, 80, 40)	100	relu	sgd	0.001	constant	0.75	0.59
(120, 80, 40)	100	relu	sgd	0.001	adaptive	0.75	0.59
(120, 80, 40)	100	relu	sgd	0.01	constant	0.75	0.59
(120, 80, 40)	100	relu	sgd	0.01	adaptive	0.75	0.59
(120, 80, 40)	100	relu	adam	0.0001	constant	0.75	0.59
(120, 80, 40)	100	relu	adam	0.0001	adaptive	0.75	0.59
(120, 80, 40)	100	relu	adam	0.001	constant	0.75	0.59
(100, 50, 25)	50	tanh	adam	0.0001	constant	0.79	0.58
(100, 50, 25)	50	relu	sgd	0.0001	adaptive	0.78	0.58
(100, 50, 25)	100	relu	adam	0.001	adaptive	0.77	0.58
(100, 50, 25)	100	relu	adam	0.01	constant	0.77	0.58
(100, 50, 25)	100	relu	adam	0.01	adaptive	0.77	0.58
(100, 50, 25)	120	tanh	sgd	0.0001	constant	0.77	0.58
(100, 50, 25)	120	tanh	sgd	0.0001	adaptive	0.77	0.58
(100, 50, 25)	120	tanh	sgd	0.001	constant	0.77	0.58
(100, 50, 25)	120	tanh	sgd	0.001	adaptive	0.77	0.58
(100, 50, 25)	120	tanh	sgd	0.01	constant	0.77	0.58
(100, 50, 25)	120	tanh	sgd	0.01	adaptive	0.77	0.58
(100, 50, 25)	120	tanh	adam	0.0001	constant	0.77	0.58
(100, 50, 25)	120	tanh	adam	0.0001	adaptive	0.77	0.58
(100, 50, 25)	120	tanh	adam	0.001	constant	0.77	0.58
(120, 80, 40)	50	tanh	adam	0.01	constant	0.76	0.58
(120, 80, 40)	50	tanh	adam	0.01	adaptive	0.76	0.58
(120, 80, 40)	50	relu	sgd	0.0001	constant	0.76	0.58
(120, 80, 40)	50	relu	sgd	0.0001	adaptive	0.76	0.58
(120, 80, 40)	50	relu	sgd	0.001	constant	0.76	0.58
(120, 80, 40)	50	relu	sgd	0.001	adaptive	0.76	0.58
(120, 80, 40)	50	relu	sgd	0.01	constant	0.76	0.58
(120, 80, 40)	50	relu	sgd	0.01	adaptive	0.76	0.58
(120, 80, 40)	50	relu	adam	0.0001	constant	0.76	0.58
(120, 80, 40)	50	relu	adam	0.0001	adaptive	0.76	0.58
(120, 80, 40)	50	relu	adam	0.001	constant	0.76	0.58
(120, 80, 40)	50	relu	adam	0.001	adaptive	0.76	0.58
(120, 80, 40)	100	relu	adam	0.001	adaptive	0.75	0.58
(120, 80, 40)	100	relu	adam	0.01	constant	0.75	0.58
(120, 80, 40)	100	relu	adam	0.01	adaptive	0.75	0.58
(120, 80, 40)	120	tanh	sgd	0.0001	constant	0.75	0.58
(120, 80, 40)	120	tanh	sgd	0.0001	adaptive	0.75	0.58
(120, 80, 40)	120	tanh	sgd	0.001	constant	0.75	0.58
(120, 80, 40)	120	tanh	sgd	0.001	adaptive	0.75	0.58
(120, 80, 40)	120	tanh	sgd	0.01	constant	0.75	0.58
(120, 80, 40)	120	tanh	sgd	0.01	adaptive	0.75	0.58
(120, 80, 40)	120	tanh	adam	0.0001	constant	0.75	0.58
(120, 80, 40)	120	tanh	adam	0.0001	adaptive	0.75	0.58
(120, 80, 40)	120	tanh	adam	0.001	constant	0.75	0.58
(120, 80, 40)	120	tanh	adam	0.001	adaptive	0.75	0.58
(120, 80, 40)	120	tanh	adam	0.01	constant	0.75	0.58
(120, 80, 40)	120	tanh	adam	0.01	adaptive	0.75	0.58
(120, 80, 40)	120	relu	sgd	0.0001	constant	0.75	0.58
(120, 80, 40)	120	relu	sgd	0.0001	adaptive	0.75	0.58
(120, 80, 40)	120	relu	sgd	0.001	constant	0.75	0.58
(120, 80, 40)	120	relu	sgd	0.001	adaptive	0.75	0.58
(120, 80, 40)	120	relu	sgd	0.01	constant	0.75	0.58
(120, 80, 40)	120	relu	sgd	0.01	adaptive	0.75	0.58
(120, 80, 40)	120	relu	adam	0.0001	constant	0.75	0.58
(120, 80, 40)	120	relu	adam	0.0001	adaptive	0.75	0.58
(120, 80, 40)	120	relu	adam	0.001	constant	0.75	0.58
(150, 75, 25)	50	tanh	sgd	0.01	adaptive	0.74	0.58
(150, 75, 25)	50	tanh	adam	0.0001	constant	0.74	0.58
(150, 75, 25)	50	tanh	adam	0.0001	adaptive	0.74	0.58
(150, 75, 25)	50	tanh	adam	0.001	constant	0.74	0.58
(150, 75, 25)	50	tanh	adam	0.001	adaptive	0.74	0.58
(150, 75, 25)	50	tanh	adam	0.01	constant	0.74	0.58
(150, 75, 25)	50	tanh	adam	0.01	adaptive	0.74	0.58
(150, 75, 25)	50	relu	sgd	0.0001	constant	0.74	0.58
(150, 75, 25)	50	relu	sgd	0.0001	adaptive	0.74	0.58
(150, 75, 25)	50	relu	sgd	0.001	constant	0.74	0.58
(150, 75, 25)	50	relu	sgd	0.001	adaptive	0.74	0.58
(150, 75, 25)	50	relu	sgd	0.01	constant	0.74	0.58
(150, 75, 25)	50	relu	sgd	0.01	adaptive	0.74	0.58
(150, 75, 25)	50	relu	adam	0.0001	constant	0.74	0.58
(150, 75, 25)	100	tanh	adam	0.01	adaptive	0.73	0.58

Cross validation :

MLP : hidden_layer_sizes =(100, 50, 25) , max_iterations= 50, activation = tanh, solver = adam, alpha = 0.001, learning_rate = constant

K	Test Accuracy	Test ROC-AUC	Test Balanced_acc	Test MCC	Test Precision	Test Recall	Test F1 score	Test Cohen's Kappa
1	0.93	0.88	0.75	0.56	0.67	0.53	0.59	0.57
2	0.93	0.9	0.75	0.55	0.67	0.52	0.59	0.54
3	0.93	0.9	0.77	0.55	0.6	0.57	0.59	0.54
4	0.93	0.88	0.74	0.54	0.66	0.51	0.58	0.53
5	0.93	0.86	0.74	0.55	0.69	0.49	0.57	0.51
Mean	0.93	0.88	0.75	0.55	0.66	0.52	0.58	0.54
SD	0	0.017	0.01	0.01	0.03	0.03	0.01	0.022
IC	0	0.88 ±1.7E-4	0.75 ±9.9E-5	0.55 ±9.9E-5	0.66 ±3.0E-4	0.52 ±3.0E-4	0.58 ±9.9E-5	0.54 ±2.1E-4

(150, 75, 25)	100	relu	sgd	0.0001	constant	0.73	0.58
(100, 50, 25)	50	tanh	sgd	0.0001	adaptive	0.8	0.57
(100, 50, 25)	50	tanh	adam	0.0001	adaptive	0.79	0.57
(100, 50, 25)	120	tanh	adam	0.001	adaptive	0.77	0.57
(120, 80, 40)	50	relu	adam	0.01	constant	0.76	0.57
(120, 80, 40)	50	relu	adam	0.01	adaptive	0.76	0.57
(120, 80, 40)	100	tanh	sgd	0.0001	constant	0.76	0.57
(120, 80, 40)	100	tanh	sgd	0.0001	adaptive	0.76	0.57
(120, 80, 40)	100	tanh	sgd	0.001	constant	0.76	0.57
(120, 80, 40)	100	tanh	sgd	0.001	adaptive	0.76	0.57
(120, 80, 40)	100	tanh	sgd	0.01	constant	0.76	0.57
(120, 80, 40)	100	tanh	sgd	0.01	adaptive	0.76	0.57
(120, 80, 40)	100	tanh	adam	0.0001	constant	0.76	0.57
(120, 80, 40)	100	tanh	adam	0.0001	adaptive	0.76	0.57
(120, 80, 40)	100	tanh	adam	0.001	constant	0.76	0.57
(120, 80, 40)	120	relu	adam	0.001	adaptive	0.75	0.57
(120, 80, 40)	120	relu	adam	0.01	constant	0.75	0.57
(120, 80, 40)	120	relu	adam	0.01	adaptive	0.75	0.57
(150, 75, 25)	50	tanh	sgd	0.0001	constant	0.75	0.57
(150, 75, 25)	50	tanh	sgd	0.0001	adaptive	0.75	0.57
(150, 75, 25)	50	tanh	sgd	0.001	constant	0.75	0.57
(150, 75, 25)	50	tanh	sgd	0.001	adaptive	0.75	0.57
(150, 75, 25)	50	tanh	sgd	0.01	constant	0.75	0.57
(150, 75, 25)	50	relu	adam	0.0001	adaptive	0.74	0.57
(150, 75, 25)	50	relu	adam	0.001	constant	0.74	0.57
(150, 75, 25)	50	relu	adam	0.001	adaptive	0.74	0.57
(150, 75, 25)	50	relu	adam	0.01	constant	0.74	0.57
(150, 75, 25)	50	relu	adam	0.01	adaptive	0.74	0.57
(150, 75, 25)	100	tanh	sgd	0.0001	constant	0.74	0.57
(150, 75, 25)	100	tanh	sgd	0.0001	adaptive	0.74	0.57
(150, 75, 25)	100	tanh	sgd	0.001	constant	0.74	0.57
(150, 75, 25)	100	tanh	sgd	0.001	adaptive	0.74	0.57
(150, 75, 25)	100	tanh	sgd	0.01	constant	0.74	0.57
(150, 75, 25)	100	tanh	sgd	0.01	adaptive	0.74	0.57
(150, 75, 25)	100	tanh	adam	0.0001	constant	0.74	0.57
(150, 75, 25)	100	relu	sgd	0.0001	adaptive	0.73	0.57
(150, 75, 25)	100	relu	sgd	0.001	constant	0.73	0.57
(150, 75, 25)	100	relu	sgd	0.001	adaptive	0.73	0.57
(150, 75, 25)	100	relu	sgd	0.01	constant	0.73	0.57
(150, 75, 25)	100	relu	sgd	0.01	adaptive	0.73	0.57
(150, 75, 25)	100	relu	adam	0.0001	constant	0.73	0.57
(150, 75, 25)	100	relu	adam	0.0001	adaptive	0.73	0.57
(100, 50, 25)	120	tanh	adam	0.01	constant	0.77	0.56
(150, 75, 25)	100	tanh	adam	0.0001	adaptive	0.74	0.56
(150, 75, 25)	100	tanh	adam	0.001	constant	0.74	0.56
(150, 75, 25)	100	tanh	adam	0.001	adaptive	0.74	0.56
(150, 75, 25)	100	tanh	adam	0.01	constant	0.74	0.56
(150, 75, 25)	100	relu	adam	0.001	constant	0.73	0.56
(150, 75, 25)	100	relu	adam	0.001	adaptive	0.73	0.56
(150, 75, 25)	100	relu	adam	0.01	constant	0.73	0.56
(150, 75, 25)	100	relu	adam	0.01	adaptive	0.73	0.56
(150, 75, 25)	120	tanh	sgd	0.0001	constant	0.73	0.56
(150, 75, 25)	120	tanh	sgd	0.0001	adaptive	0.73	0.56
(150, 75, 25)	120	tanh	sgd	0.001	constant	0.73	0.56
(150, 75, 25)	120	tanh	sgd	0.001	adaptive	0.73	0.56
(150, 75, 25)	120	tanh	sgd	0.01	constant	0.73	0.56
(150, 75, 25)	120	tanh	sgd	0.01	adaptive	0.73	0.56
(150, 75, 25)	120	tanh	adam	0.0001	constant	0.73	0.56
(150, 75, 25)	120	tanh	adam	0.0001	adaptive	0.73	0.56
(150, 75, 25)	120	tanh	adam	0.01	constant	0.72	0.56
(150, 75, 25)	120	tanh	adam	0.01	adaptive	0.72	0.56
(150, 75, 25)	120	relu	sgd	0.0001	constant	0.72	0.56
(150, 75, 25)	120	relu	sgd	0.0001	adaptive	0.72	0.56
(150, 75, 25)	120	relu	sgd	0.001	constant	0.72	0.56
(150, 75, 25)	120	tanh	adam	0.001	constant	0.73	0.55
(150, 75, 25)	120	tanh	adam	0.001	adaptive	0.73	0.55
(150, 75, 25)	120	relu	sgd	0.001	adaptive	0.72	0.55
(150, 75, 25)	120	relu	sgd	0.01	constant	0.72	0.55
(150, 75, 25)	120	relu	sgd	0.01	adaptive	0.72	0.55
(150, 75, 25)	120	relu	adam	0.0001	constant	0.72	0.55
(150, 75, 25)	120	relu	adam	0.0001	adaptive	0.72	0.55
(150, 75, 25)	120	relu	adam	0.001	constant	0.71	0.55
(150, 75, 25)	120	relu	adam	0.001	adaptive	0.71	0.54
(150, 75, 25)	120	relu	adam	0.01	constant	0.71	0.54
(150, 75, 25)	120	relu	adam	0.01	adaptive	0.7	0.53

XGBoost trained on Trypanosomiasis dataset

learning_rate	n_estimators	subsample	max_depth	colsample_bytree	Balanced Accuracy	F1 -score
0.5	50	1	6	1	0.8	0.7
0.5	50	0.5	6	0.5	0.8	0.69
1	50	1	6	1	0.79	0.69
1	50	0.8	6	1	0.79	0.67
0.5	50	0.8	6	1	0.78	0.67
0.25	50	1	6	1	0.76	0.67
1	50	0.8	6	0.5	0.78	0.66
1	50	1	6	0.8	0.78	0.66
0.5	50	1	4	1	0.77	0.66
0.5	50	0.8	6	0.8	0.77	0.66
0.25	50	0.5	6	0.8	0.76	0.66
0.25	50	0.8	6	1	0.76	0.66
0.5	20	0.5	6	0.5	0.78	0.65
1	50	0.8	6	0.8	0.78	0.65
1	50	1	6	0.5	0.77	0.65
0.25	50	0.5	6	0.5	0.76	0.65
0.5	50	0.8	6	0.5	0.76	0.65
0.25	50	0.8	6	0.8	0.76	0.65
0.25	50	1	6	0.5	0.75	0.65
0.25	50	1	6	0.8	0.75	0.65
1	50	1	4	0.5	0.78	0.64
1	50	1	4	1	0.78	0.64
0.5	50	0.5	6	1	0.77	0.64
1	20	1	6	0.8	0.76	0.64
0.5	20	1	6	1	0.76	0.64
0.5	50	1	6	0.8	0.76	0.64
0.25	50	0.5	6	1	0.75	0.64
0.25	50	0.8	6	0.5	0.75	0.64
1	50	1	4	0.8	0.78	0.63
1	20	0.8	6	1	0.77	0.63
0.5	50	0.5	4	1	0.77	0.63
0.5	50	0.5	6	0.8	0.76	0.63
0.5	50	1	6	0.5	0.75	0.63
1	10	1	6	0.8	0.77	0.62
1	50	0.8	4	1	0.77	0.62
1	20	1	6	1	0.76	0.62
0.5	20	0.8	6	0.8	0.75	0.62
0.5	50	1	4	0.5	0.75	0.62
0.5	50	1	4	0.8	0.75	0.62
0.5	20	0.8	6	1	0.74	0.62
0.5	50	0.8	4	0.8	0.74	0.62
1	20	0.8	6	0.8	0.77	0.61
1	8	1	6	0.8	0.76	0.61
0.5	50	0.8	4	1	0.74	0.61
1	10	1	6	1	0.76	0.6
1	20	0.8	6	0.5	0.76	0.6
1	20	1	4	1	0.75	0.6
0.5	50	0.5	4	0.5	0.75	0.6
0.25	50	1	4	0.5	0.73	0.6
0.25	50	1	4	0.8	0.73	0.6
0.5	50	0.5	4	0.8	0.74	0.59
0.5	20	1	6	0.5	0.73	0.59
0.5	20	1	6	0.8	0.73	0.59
0.25	50	1	4	1	0.72	0.59
1	50	0.8	4	0.5	0.77	0.58
1	50	0.5	6	0.5	0.76	0.58
1	10	0.8	6	1	0.75	0.58
1	50	1	2	0.8	0.74	0.58
1	10	1	4	1	0.73	0.58
0.5	20	0.5	6	1	0.73	0.58
0.25	50	0.5	4	0.8	0.72	0.58
0.5	50	0.8	4	0.5	0.72	0.58
0.25	50	0.8	4	0.8	0.72	0.58
0.25	50	0.8	4	1	0.72	0.58
1	50	0.5	6	0.8	0.76	0.57
1	50	0.8	4	0.8	0.75	0.57
1	20	1	4	0.5	0.74	0.57
1	20	1	6	0.5	0.74	0.57
0.5	20	0.5	6	0.8	0.73	0.57
0.5	10	0.5	6	0.5	0.72	0.57
0.25	20	0.5	6	1	0.71	0.57
0.25	50	0.8	4	0.5	0.71	0.57
0.1	50	0.5	6	0.8	0.71	0.57
0.1	50	1	6	1	0.71	0.57
1	50	0.8	2	0.8	0.74	0.56
0.5	10	1	6	1	0.72	0.56
0.25	50	0.5	4	1	0.71	0.56
0.25	20	1	6	0.8	0.7	0.56
0.1	50	0.5	6	1	0.7	0.56
0.1	50	0.8	6	0.8	0.7	0.56
0.1	50	0.8	6	1	0.7	0.56
1	10	0.8	6	0.5	0.74	0.55
1	8	0.8	6	1	0.73	0.55
1	8	1	6	1	0.73	0.55
1	20	1	4	0.8	0.73	0.55
1	50	0.8	2	1	0.72	0.55
0.5	8	1	6	0.8	0.71	0.55
0.5	10	0.8	6	0.8	0.71	0.55
0.5	10	0.8	6	1	0.71	0.55
0.5	20	1	4	0.5	0.71	0.55
0.5	20	1	4	1	0.71	0.55
0.5	20	0.8	6	0.5	0.71	0.55
0.25	50	0.5	4	0.5	0.71	0.55
0.25	20	0.5	6	0.5	0.7	0.55
0.25	20	0.5	6	0.8	0.7	0.55
0.5	50	0.5	2	0.5	0.7	0.55
1	8	0.8	6	0.5	0.73	0.54
1	8	1	6	0.5	0.72	0.54
0.5	10	0.5	6	0.8	0.71	0.54
0.5	10	1	6	0.8	0.7	0.54
0.5	20	0.5	4	0.8	0.7	0.54
0.5	20	0.5	4	1	0.7	0.54
0.5	20	0.8	4	0.8	0.7	0.54
0.25	20	1	6	1	0.7	0.54
0.25	20	0.8	6	1	0.69	0.54
0.1	50	0.5	6	0.5	0.69	0.54
0.1	50	1	6	0.5	0.69	0.54
1	20	0.5	6	0.8	0.75	0.53
1	50	0.5	6	1	0.74	0.53
1	10	0.8	6	0.8	0.73	0.53
1	20	0.8	4	1	0.73	0.53
1	10	1	6	0.5	0.72	0.53
1	50	0.5	2	0.5	0.71	0.53
1	50	1	2	1	0.71	0.53
0.5	8	0.8	6	1	0.7	0.53
0.5	50	0.5	2	0.8	0.7	0.53
0.25	20	0.8	6	0.5	0.69	0.53
0.5	50	0.5	2	1	0.69	0.53
0.25	20	1	6	0.5	0.68	0.53
1	20	0.5	4	0.5	0.74	0.52
1	50	0.5	4	0.8	0.74	0.52

Cross validation :

XGBoost : learning_rate = 0,5, n_estimators = 50, subsample= 1, max_depth = 6, colsample_bytree = 1

K	Test Accuracy	Test ROC-AUC	Test Balanced_acc	Test MCC	Test Precision	Test Recall	Test F1 score	Test Cohen's Kappa
1	0.95	0.9	0.74	0.61	0.8	0.5	0.61	0.59
2	0.95	0.9	0.74	0.62	0.85	0.49	0.62	0.59
3	0.96	0.89	0.75	0.64	0.87	0.5	0.64	0.61
4	0.95	0.92	0.76	0.61	0.76	0.53	0.63	0.56
5	0.96	0.89	0.75	0.64	0.87	0.5	0.64	0.61
Mean	0.95	0.9	0.75	0.62	0.83	0.5	0.63	0.59
SD	0.005	0.012	0.01	0.015	0.05	0.015	0.013	0.02
IC	0.95 ±7.73E-05	0.90 ±1.8E-4	0.75 ±1.2E-4	0.62 ±2.3E-4	0.83 ±7.4E-4	0.50 ±2.3E-4	0.63 ±2.0E-4	0.59 ±3.0E-4

1	10	0.5	4	0.8	0.72	0.52
1	20	0.8	4	0.5	0.72	0.52
1	8	0.8	6	0.8	0.71	0.52
1	10	0.8	4	0.8	0.71	0.52
1	10	1	4	0.5	0.71	0.52
1	20	0.8	4	0.8	0.71	0.52
1	50	0.8	2	0.5	0.71	0.52
1	8	1	4	1	0.7	0.52
1	50	1	2	0.5	0.7	0.52
0.5	10	0.5	6	1	0.69	0.52
0.1	50	0.8	6	0.5	0.68	0.52
0.1	50	1	6	0.8	0.68	0.52
1	50	0.5	4	1	0.74	0.51
1	10	0.8	4	0.5	0.7	0.51
0.5	8	0.8	6	0.8	0.69	0.51
0.5	20	0.8	4	0.5	0.69	0.51
0.5	20	0.8	4	1	0.69	0.51
0.5	20	1	4	0.8	0.69	0.51
0.5	10	0.8	6	0.5	0.68	0.51
1	50	0.5	4	0.5	0.73	0.5
1	50	0.5	2	0.8	0.72	0.5
1	8	0.5	4	0.8	0.71	0.5
1	50	0.5	2	1	0.71	0.5
1	10	0.8	4	1	0.7	0.5
0.5	20	0.5	4	0.5	0.69	0.5
0.5	8	0.5	6	0.5	0.68	0.5
0.25	20	0.8	6	0.8	0.68	0.5
0.5	50	0.8	2	1	0.68	0.5
0.5	50	1	2	1	0.68	0.5
1	20	0.5	6	0.5	0.72	0.49
1	20	0.5	6	1	0.72	0.49
1	8	0.5	6	0.5	0.71	0.49
1	10	0.5	4	1	0.7	0.49
1	8	0.5	4	1	0.69	0.49
1	20	0.8	2	1	0.68	0.49
0.5	8	1	6	1	0.67	0.49
0.5	50	0.8	2	0.5	0.67	0.49
1	20	0.5	4	1	0.72	0.48
1	10	0.5	6	0.5	0.71	0.48
1	8	0.8	4	0.8	0.69	0.48
1	20	0.5	2	1	0.69	0.48
1	8	1	4	0.5	0.68	0.48
0.5	8	0.5	6	0.8	0.67	0.48
0.5	8	0.5	6	1	0.67	0.48
0.5	8	1	6	0.5	0.67	0.48
0.5	10	1	6	0.5	0.67	0.48
0.5	50	1	2	0.8	0.67	0.48
1	8	1	4	0.8	0.68	0.47
0.5	8	0.8	6	0.5	0.66	0.47
1	20	0.5	4	0.8	0.7	0.46
1	10	0.5	6	0.8	0.69	0.46
1	2	0.8	6	1	0.68	0.46
1	2	1	6	1	0.68	0.46
1	8	0.5	4	0.5	0.68	0.46
1	8	0.8	4	1	0.68	0.46
1	2	1	6	0.5	0.67	0.46
1	10	1	4	0.8	0.67	0.46
1	20	0.5	2	0.5	0.67	0.46
1	20	1	2	0.5	0.66	0.46
1	10	0.5	6	1	0.7	0.45
1	8	0.5	6	0.8	0.69	0.45
1	8	0.5	6	1	0.69	0.45
1	20	1	2	1	0.67	0.45
0.25	10	0.5	6	1	0.65	0.45
1	20	0.8	2	0.8	0.67	0.44
1	8	0.8	4	0.5	0.66	0.44
0.5	10	0.5	4	1	0.65	0.44
0.25	10	0.8	6	1	0.65	0.44
0.25	20	1	4	0.5	0.65	0.44
0.5	50	0.8	2	0.8	0.65	0.44
0.5	10	1	4	1	0.64	0.44
1	2	0.5	6	0.8	0.68	0.43
1	10	0.5	4	0.5	0.67	0.43
0.5	10	0.5	4	0.8	0.65	0.43
0.5	10	0.8	4	1	0.64	0.43
0.25	10	0.5	6	0.8	0.64	0.43
0.25	10	0.8	6	0.5	0.64	0.43
0.25	20	0.5	4	1	0.64	0.43
0.25	20	1	4	0.8	0.64	0.43
0.5	10	0.5	4	0.5	0.64	0.42
0.25	20	1	4	1	0.64	0.42
1	2	0.8	6	0.8	0.65	0.41
1	20	0.5	2	0.8	0.65	0.41
0.5	10	1	4	0.8	0.64	0.41
1	20	0.8	2	0.5	0.64	0.41
1	20	1	2	0.8	0.64	0.41
0.5	8	1	4	1	0.63	0.41
0.25	50	0.5	2	0.8	0.63	0.41
0.1	50	0.5	4	0.8	0.63	0.41
0.1	50	0.5	4	1	0.63	0.41
0.1	50	0.8	4	1	0.63	0.41
1	2	0.5	6	0.5	0.66	0.4
1	10	0.5	2	1	0.64	0.4
0.5	10	0.8	4	0.8	0.64	0.4
0.5	8	0.5	4	0.5	0.63	0.4
0.25	10	1	6	0.8	0.63	0.4
0.25	10	1	6	1	0.63	0.4
0.25	20	0.8	4	0.5	0.63	0.4
0.5	50	1	2	0.5	0.63	0.4
1	2	1	6	0.8	0.65	0.39
1	10	0.8	2	0.8	0.63	0.39
0.5	8	1	4	0.8	0.62	0.39
0.25	8	0.8	6	1	0.62	0.39
0.25	20	0.5	4	0.8	0.62	0.39
0.25	20	0.8	4	0.8	0.62	0.39
1	2	0.5	6	1	0.65	0.38
0.5	8	0.5	4	0.8	0.62	0.38
0.25	8	0.5	6	1	0.62	0.38
0.5	10	0.8	4	0.5	0.62	0.38
0.25	10	0.5	6	0.5	0.62	0.38
0.25	10	1	6	0.5	0.62	0.38
0.1	50	0.8	4	0.5	0.62	0.38
1	10	0.8	2	1	0.62	0.37
0.5	10	1	4	0.5	0.62	0.37
0.25	20	0.8	4	1	0.62	0.37
0.1	20	0.5	6	0.8	0.62	0.37
0.25	50	0.5	2	1	0.62	0.37
0.1	50	0.8	4	0.8	0.62	0.37
0.1	50	1	4	1	0.62	0.37
0.25	8	1	6	0.8	0.61	0.37
0.5	20	0.5	2	1	0.61	0.37
0.1	20	0.8	6	0.5	0.61	0.37
1	10	0.5	2	0.8	0.62	0.36
0.5	2	0.5	6	0.8	0.61	0.36

0.5	8	0.5	4	1	0.61	0.36
0.25	8	0.5	6	0.5	0.61	0.36
0.25	8	0.5	6	0.8	0.61	0.36
0.1	20	0.8	6	0.8	0.61	0.36
0.1	20	1	6	0.5	0.61	0.36
0.25	50	0.8	2	1	0.61	0.36
0.1	50	0.5	4	0.5	0.61	0.36
0.1	50	1	4	0.8	0.61	0.36
0.5	2	0.8	6	1	0.61	0.35
0.5	20	0.5	2	0.8	0.61	0.35
0.1	20	0.5	6	1	0.61	0.35
1	2	0.8	6	0.5	0.62	0.34
0.5	2	0.8	6	0.8	0.61	0.34
0.5	8	0.8	4	0.5	0.61	0.34
0.25	20	0.5	4	0.5	0.61	0.34
0.25	8	0.8	6	0.5	0.6	0.34
0.25	8	1	6	0.5	0.6	0.34
0.25	50	1	2	1	0.6	0.34
0.1	50	1	4	0.5	0.6	0.34
0.5	8	0.8	4	1	0.6	0.33
0.5	20	0.8	2	1	0.6	0.33
0.1	20	0.5	6	0.5	0.6	0.33
0.1	20	0.8	6	1	0.6	0.33
0.1	20	1	6	1	0.6	0.33
0.25	50	1	2	0.5	0.6	0.33
0.25	50	1	2	0.8	0.6	0.33
1	2	0.5	4	0.5	0.61	0.32
1	2	0.8	4	1	0.61	0.32
0.5	2	0.5	6	0.5	0.6	0.32
1	8	0.5	2	0.5	0.6	0.32
1	8	0.5	2	1	0.6	0.32
0.5	8	0.8	4	0.8	0.6	0.32
0.5	8	1	4	0.5	0.6	0.32
0.25	8	1	6	1	0.6	0.32
1	10	0.5	2	0.5	0.6	0.32
0.25	10	0.8	6	0.8	0.6	0.32
0.5	20	1	2	0.5	0.6	0.32
0.5	20	1	2	1	0.6	0.32
0.1	20	1	6	0.8	0.6	0.32
0.25	50	0.5	2	0.5	0.6	0.32
0.25	50	0.8	2	0.8	0.6	0.32
0.5	2	0.5	6	1	0.6	0.31
0.5	2	1	6	1	0.6	0.31
1	10	1	2	0.8	0.6	0.31
1	10	1	2	1	0.6	0.31
0.5	20	1	2	0.8	0.59	0.31
1	2	0.8	4	0.5	0.6	0.3
1	8	0.5	2	0.8	0.59	0.3
0.25	8	0.8	6	0.8	0.59	0.3
0.5	20	0.5	2	0.5	0.59	0.3
0.5	20	0.8	2	0.8	0.59	0.3
0.5	2	1	6	0.8	0.59	0.29
0.5	20	0.8	2	0.5	0.59	0.29
0.25	50	0.8	2	0.5	0.59	0.29
1	2	0.5	4	1	0.59	0.28
1	10	1	2	0.5	0.59	0.28
1	8	0.8	2	1	0.58	0.28
1	8	0.8	2	0.8	0.58	0.27
1	8	1	2	0.8	0.58	0.27
1	8	1	2	1	0.58	0.27
1	10	0.8	2	0.5	0.58	0.27
0.25	10	0.5	4	0.8	0.58	0.27
1	2	0.5	4	0.8	0.58	0.26
1	2	1	4	0.8	0.58	0.26
1	2	1	4	1	0.58	0.26
0.5	2	1	6	0.5	0.58	0.26
0.25	10	0.8	4	1	0.57	0.26
1	8	0.8	2	0.5	0.57	0.25
1	2	0.8	4	0.8	0.57	0.24
1	8	1	2	0.5	0.57	0.24
0.25	10	1	4	1	0.57	0.24
0.25	10	0.8	4	0.5	0.57	0.23
0.5	2	0.5	4	0.5	0.56	0.23
0.25	10	0.5	4	0.5	0.56	0.21
0.25	10	0.8	4	0.8	0.56	0.2
0.25	10	0.5	4	1	0.55	0.19
0.25	10	1	4	0.8	0.55	0.19
0.1	10	0.5	6	0.8	0.55	0.19
0.25	8	0.8	4	1	0.55	0.18
0.25	10	1	4	0.5	0.55	0.18
0.5	2	0.8	6	0.5	0.55	0.17
0.5	10	0.5	2	0.5	0.54	0.15
0.1	10	0.5	6	0.5	0.54	0.15
1	2	1	4	0.5	0.54	0.14
0.25	8	0.5	4	0.8	0.54	0.14
0.25	8	1	4	1	0.54	0.14
0.1	20	0.5	4	0.8	0.54	0.14
0.1	10	0.5	6	1	0.54	0.13
0.1	20	0.5	4	0.5	0.54	0.13
0.1	20	0.5	4	1	0.54	0.13
0.25	8	0.5	4	0.5	0.53	0.13
0.1	10	0.8	6	1	0.53	0.13
0.1	10	1	6	0.8	0.53	0.13
0.5	2	0.5	4	1	0.53	0.12
0.1	10	1	6	1	0.53	0.12
0.25	20	0.5	2	1	0.53	0.12
0.25	8	0.5	4	1	0.53	0.11
0.25	8	0.8	4	0.8	0.53	0.11
0.25	8	1	4	0.5	0.53	0.11
0.25	8	1	4	0.8	0.53	0.11
0.5	10	0.5	2	1	0.53	0.11
0.1	8	1	6	1	0.52	0.09
0.5	10	0.5	2	0.8	0.52	0.09
0.5	10	0.8	2	1	0.52	0.09
0.1	10	0.8	6	0.5	0.52	0.09
0.1	10	0.8	6	0.8	0.52	0.09
0.25	20	0.5	2	0.8	0.52	0.09
0.1	20	0.8	4	0.5	0.52	0.09
0.1	20	0.8	4	0.8	0.52	0.09
0.1	20	0.8	4	1	0.52	0.09
0.1	20	1	4	0.5	0.52	0.09
0.5	2	0.5	4	0.8	0.52	0.08
0.25	8	0.8	4	0.5	0.52	0.08
0.5	10	0.8	2	0.5	0.52	0.08
0.1	10	1	6	0.5	0.52	0.08
0.25	20	0.5	2	0.5	0.52	0.08
0.1	20	1	4	1	0.52	0.08
0.5	10	0.8	2	0.8	0.52	0.07
0.5	10	1	2	1	0.52	0.07
0.1	20	1	4	0.8	0.52	0.07
0.1	50	0.5	2	0.8	0.52	0.07
0.1	50	0.5	2	1	0.52	0.07
0.1	8	0.5	6	0.8	0.51	0.05
0.5	10	1	2	0.8	0.51	0.05

0.5	8	0.5	2	1	0.51	0.04
0.1	8	0.5	6	0.5	0.51	0.04
0.5	10	1	2	0.5	0.51	0.04
0.25	20	0.8	2	1	0.51	0.04
0.25	20	1	2	1	0.51	0.04
0.1	50	0.8	2	0.8	0.51	0.04
0.5	8	0.5	2	0.5	0.51	0.03
0.5	8	1	2	1	0.51	0.03
0.1	8	0.5	6	1	0.51	0.03
0.1	8	0.8	6	0.8	0.51	0.03
0.1	8	0.8	6	1	0.51	0.03
0.25	20	0.8	2	0.8	0.51	0.03
0.25	20	1	2	0.5	0.51	0.03
0.25	20	1	2	0.8	0.51	0.03
0.1	50	0.5	2	0.5	0.51	0.03
0.1	50	0.8	2	0.5	0.51	0.03
0.1	50	0.8	2	1	0.51	0.03
0.1	50	1	2	0.8	0.51	0.03
0.1	50	1	2	1	0.51	0.03
0.5	8	0.5	2	0.8	0.5	0.01
0.5	8	0.8	2	0.5	0.5	0.01
0.5	8	0.8	2	0.8	0.5	0.01
0.5	8	0.8	2	1	0.5	0.01
0.5	8	1	2	0.5	0.5	0.01
0.1	8	0.8	6	0.5	0.5	0.01
0.1	8	1	6	0.5	0.5	0.01
0.1	8	1	6	0.8	0.5	0.01
0.25	20	0.8	2	0.5	0.5	0.01
0.1	50	1	2	0.5	0.5	0.01
0.01	2	0.5	2	0.5	0.5	0
0.1	2	0.5	2	0.5	0.5	0
0.25	2	0.5	2	0.5	0.5	0
0.5	2	0.5	2	0.5	0.5	0
1	2	0.5	2	0.5	0.5	0
0.01	2	0.5	2	0.8	0.5	0
0.1	2	0.5	2	0.8	0.5	0
0.25	2	0.5	2	0.8	0.5	0
0.5	2	0.5	2	0.8	0.5	0
1	2	0.5	2	0.8	0.5	0
0.01	2	0.5	2	1	0.5	0
0.1	2	0.5	2	1	0.5	0
0.25	2	0.5	2	1	0.5	0
0.5	2	0.5	2	1	0.5	0
1	2	0.5	2	1	0.5	0
0.01	2	0.8	2	0.5	0.5	0
0.1	2	0.8	2	0.5	0.5	0
0.25	2	0.8	2	0.5	0.5	0
0.5	2	0.8	2	0.5	0.5	0
1	2	0.8	2	0.5	0.5	0
0.01	2	0.8	2	0.8	0.5	0
0.1	2	0.8	2	0.8	0.5	0
0.25	2	0.8	2	0.8	0.5	0
0.5	2	0.8	2	0.8	0.5	0
1	2	0.8	2	0.8	0.5	0
0.01	2	0.8	2	1	0.5	0
0.1	2	0.8	2	1	0.5	0
0.25	2	0.8	2	1	0.5	0
0.5	2	0.8	2	1	0.5	0
1	2	0.8	2	1	0.5	0
0.01	2	1	2	0.5	0.5	0
0.1	2	1	2	0.5	0.5	0
0.25	2	1	2	0.5	0.5	0
0.5	2	1	2	0.5	0.5	0
1	2	1	2	0.5	0.5	0
0.01	2	1	2	0.8	0.5	0
0.1	2	1	2	0.8	0.5	0
0.25	2	1	2	0.8	0.5	0
0.5	2	1	2	0.8	0.5	0
1	2	1	2	0.8	0.5	0
0.01	2	1	2	1	0.5	0
0.1	2	1	2	1	0.5	0
0.25	2	1	2	1	0.5	0
0.5	2	1	2	1	0.5	0
1	2	1	2	1	0.5	0
0.01	2	0.5	4	0.5	0.5	0
0.1	2	0.5	4	0.5	0.5	0
0.25	2	0.5	4	0.5	0.5	0
0.01	2	0.5	4	0.8	0.5	0
0.1	2	0.5	4	0.8	0.5	0
0.25	2	0.5	4	0.8	0.5	0
0.01	2	0.5	4	1	0.5	0
0.1	2	0.5	4	1	0.5	0
0.25	2	0.5	4	1	0.5	0
0.01	2	0.8	4	0.5	0.5	0
0.1	2	0.8	4	0.5	0.5	0
0.25	2	0.8	4	0.5	0.5	0
0.5	2	0.8	4	0.5	0.5	0
0.01	2	0.8	4	0.8	0.5	0
0.1	2	0.8	4	0.8	0.5	0
0.25	2	0.8	4	0.8	0.5	0
0.5	2	0.8	4	0.8	0.5	0
0.01	2	0.8	4	1	0.5	0
0.1	2	0.8	4	1	0.5	0
0.25	2	0.8	4	1	0.5	0
0.5	2	0.8	4	1	0.5	0
0.01	2	1	4	0.5	0.5	0
0.1	2	1	4	0.5	0.5	0
0.25	2	1	4	0.5	0.5	0
0.5	2	1	4	0.5	0.5	0
0.01	2	1	4	0.8	0.5	0
0.1	2	1	4	0.8	0.5	0
0.25	2	1	4	0.8	0.5	0
0.5	2	1	4	0.8	0.5	0
0.01	2	1	4	1	0.5	0
0.1	2	1	4	1	0.5	0
0.25	2	1	4	1	0.5	0
0.5	2	1	4	1	0.5	0
0.01	2	0.5	6	0.5	0.5	0
0.1	2	0.5	6	0.5	0.5	0
0.25	2	0.5	6	0.5	0.5	0
0.01	2	0.5	6	0.8	0.5	0
0.1	2	0.5	6	0.8	0.5	0
0.25	2	0.5	6	0.8	0.5	0
0.01	2	0.5	6	1	0.5	0
0.1	2	0.5	6	1	0.5	0
0.25	2	0.5	6	1	0.5	0
0.01	2	0.8	6	0.5	0.5	0
0.1	2	0.8	6	0.5	0.5	0
0.25	2	0.8	6	0.5	0.5	0
0.01	2	0.8	6	0.8	0.5	0
0.1	2	0.8	6	0.8	0.5	0
0.25	2	0.8	6	0.8	0.5	0
0.01	2	0.8	6	1	0.5	0
0.1	2	0.8	6	1	0.5	0

0.25	2	0.8	6	1	0.5	0
0.01	2	1	6	0.5	0.5	0
0.1	2	1	6	0.5	0.5	0
0.25	2	1	6	0.5	0.5	0
0.01	2	1	6	0.8	0.5	0
0.1	2	1	6	0.8	0.5	0
0.25	2	1	6	0.8	0.5	0
0.01	2	1	6	1	0.5	0
0.1	2	1	6	1	0.5	0
0.25	2	1	6	1	0.5	0
0.01	8	0.5	2	0.5	0.5	0
0.1	8	0.5	2	0.5	0.5	0
0.25	8	0.5	2	0.5	0.5	0
0.01	8	0.5	2	0.8	0.5	0
0.1	8	0.5	2	0.8	0.5	0
0.25	8	0.5	2	0.8	0.5	0
0.01	8	0.5	2	1	0.5	0
0.1	8	0.5	2	1	0.5	0
0.25	8	0.5	2	1	0.5	0
0.01	8	0.8	2	0.5	0.5	0
0.1	8	0.8	2	0.5	0.5	0
0.25	8	0.8	2	0.5	0.5	0
0.01	8	0.8	2	0.8	0.5	0
0.1	8	0.8	2	0.8	0.5	0
0.25	8	0.8	2	0.8	0.5	0
0.01	8	0.8	2	1	0.5	0
0.1	8	0.8	2	1	0.5	0
0.25	8	0.8	2	1	0.5	0
0.01	8	1	2	0.5	0.5	0
0.1	8	1	2	0.5	0.5	0
0.25	8	1	2	0.5	0.5	0
0.01	8	1	2	0.8	0.5	0
0.1	8	1	2	0.8	0.5	0
0.25	8	1	2	0.8	0.5	0
0.5	8	1	2	0.8	0.5	0
0.01	8	1	2	1	0.5	0
0.1	8	1	2	1	0.5	0
0.25	8	1	2	1	0.5	0
0.01	8	0.5	4	0.5	0.5	0
0.1	8	0.5	4	0.5	0.5	0
0.01	8	0.5	4	0.8	0.5	0
0.1	8	0.5	4	0.8	0.5	0
0.01	8	0.5	4	1	0.5	0
0.1	8	0.5	4	1	0.5	0
0.01	8	0.8	4	0.5	0.5	0
0.1	8	0.8	4	0.5	0.5	0
0.01	8	0.8	4	0.8	0.5	0
0.1	8	0.8	4	0.8	0.5	0
0.01	8	0.8	4	1	0.5	0
0.1	8	0.8	4	1	0.5	0
0.01	8	1	4	0.5	0.5	0
0.1	8	1	4	0.5	0.5	0
0.01	8	1	4	0.8	0.5	0
0.1	8	1	4	0.8	0.5	0
0.01	8	1	4	1	0.5	0
0.1	8	1	4	1	0.5	0
0.01	8	0.5	6	0.5	0.5	0
0.01	8	0.5	6	0.8	0.5	0
0.01	8	0.5	6	1	0.5	0
0.01	8	0.8	6	0.5	0.5	0
0.01	8	0.8	6	0.8	0.5	0
0.01	8	0.8	6	1	0.5	0
0.01	8	1	6	0.5	0.5	0
0.01	8	1	6	0.8	0.5	0
0.01	8	1	6	1	0.5	0
0.01	10	0.5	2	0.5	0.5	0
0.1	10	0.5	2	0.5	0.5	0
0.25	10	0.5	2	0.5	0.5	0
0.01	10	0.5	2	0.8	0.5	0
0.1	10	0.5	2	0.8	0.5	0
0.25	10	0.5	2	0.8	0.5	0
0.01	10	0.5	2	1	0.5	0
0.1	10	0.5	2	1	0.5	0
0.25	10	0.5	2	1	0.5	0
0.01	10	0.8	2	0.5	0.5	0
0.1	10	0.8	2	0.5	0.5	0
0.25	10	0.8	2	0.5	0.5	0
0.01	10	0.8	2	0.8	0.5	0
0.1	10	0.8	2	0.8	0.5	0
0.25	10	0.8	2	0.8	0.5	0
0.01	10	0.8	2	1	0.5	0
0.1	10	0.8	2	1	0.5	0
0.25	10	0.8	2	1	0.5	0
0.01	10	1	2	0.5	0.5	0
0.1	10	1	2	0.5	0.5	0
0.25	10	1	2	0.5	0.5	0
0.01	10	1	2	0.8	0.5	0
0.1	10	1	2	0.8	0.5	0
0.25	10	1	2	0.8	0.5	0
0.01	10	1	2	1	0.5	0
0.1	10	1	2	1	0.5	0
0.25	10	1	2	1	0.5	0
0.01	10	0.5	4	0.5	0.5	0
0.1	10	0.5	4	0.5	0.5	0
0.01	10	0.5	4	0.8	0.5	0
0.1	10	0.5	4	0.8	0.5	0
0.01	10	0.5	4	1	0.5	0
0.1	10	0.5	4	1	0.5	0
0.01	10	0.8	4	0.5	0.5	0
0.1	10	0.8	4	0.5	0.5	0
0.01	10	0.8	4	0.8	0.5	0
0.1	10	0.8	4	0.8	0.5	0
0.01	10	0.8	4	1	0.5	0
0.1	10	0.8	4	1	0.5	0
0.01	10	1	4	0.5	0.5	0
0.1	10	1	4	0.5	0.5	0
0.01	10	1	4	0.8	0.5	0
0.1	10	1	4	0.8	0.5	0
0.01	10	1	4	1	0.5	0
0.1	10	1	4	1	0.5	0
0.01	10	0.5	6	0.5	0.5	0
0.01	10	0.5	6	0.8	0.5	0
0.01	10	0.5	6	1	0.5	0
0.01	10	0.8	6	0.5	0.5	0
0.01	10	0.8	6	0.8	0.5	0
0.01	10	0.8	6	1	0.5	0
0.01	10	1	6	0.5	0.5	0
0.01	10	1	6	0.8	0.5	0
0.01	10	1	6	1	0.5	0
0.01	20	0.5	2	0.5	0.5	0
0.1	20	0.5	2	0.5	0.5	0
0.01	20	0.5	2	0.8	0.5	0
0.1	20	0.5	2	0.8	0.5	0
0.01	20	0.5	2	1	0.5	0

0.1	20	0.5	2	1	0.5	0
0.01	20	0.8	2	0.5	0.5	0
0.1	20	0.8	2	0.5	0.5	0
0.01	20	0.8	2	0.8	0.5	0
0.1	20	0.8	2	0.8	0.5	0
0.01	20	0.8	2	1	0.5	0
0.1	20	0.8	2	1	0.5	0
0.01	20	1	2	0.5	0.5	0
0.1	20	1	2	0.5	0.5	0
0.01	20	1	2	0.8	0.5	0
0.1	20	1	2	0.8	0.5	0
0.01	20	1	2	1	0.5	0
0.1	20	1	2	1	0.5	0
0.01	20	0.5	4	0.5	0.5	0
0.01	20	0.5	4	0.8	0.5	0
0.01	20	0.5	4	1	0.5	0
0.01	20	0.8	4	0.5	0.5	0
0.01	20	0.8	4	0.8	0.5	0
0.01	20	0.8	4	1	0.5	0
0.01	20	1	4	0.5	0.5	0
0.01	20	1	4	0.8	0.5	0
0.01	20	1	4	1	0.5	0
0.01	20	0.5	6	0.5	0.5	0
0.01	20	0.5	6	0.8	0.5	0
0.01	20	0.5	6	1	0.5	0
0.01	20	0.8	6	0.5	0.5	0
0.01	20	0.8	6	0.8	0.5	0
0.01	20	0.8	6	1	0.5	0
0.01	20	1	6	0.5	0.5	0
0.01	20	1	6	0.8	0.5	0
0.01	20	1	6	1	0.5	0
0.01	50	0.5	2	0.5	0.5	0
0.01	50	0.5	2	0.8	0.5	0
0.01	50	0.5	2	1	0.5	0
0.01	50	0.8	2	0.5	0.5	0
0.01	50	0.8	2	0.8	0.5	0
0.01	50	0.8	2	1	0.5	0
0.01	50	1	2	0.5	0.5	0
0.01	50	1	2	0.8	0.5	0
0.01	50	1	2	1	0.5	0
0.01	50	0.5	4	0.5	0.5	0
0.01	50	0.5	4	0.8	0.5	0
0.01	50	0.5	4	1	0.5	0
0.01	50	0.8	4	0.5	0.5	0
0.01	50	0.8	4	0.8	0.5	0
0.01	50	0.8	4	1	0.5	0
0.01	50	1	4	0.5	0.5	0
0.01	50	1	4	0.8	0.5	0
0.01	50	1	4	1	0.5	0
0.01	50	0.5	6	0.5	0.5	0
0.01	50	0.5	6	0.8	0.5	0
0.01	50	0.5	6	1	0.5	0
0.01	50	0.8	6	0.5	0.5	0
0.01	50	0.8	6	0.8	0.5	0
0.01	50	0.8	6	1	0.5	0
0.01	50	1	6	0.5	0.5	0
0.01	50	1	6	0.8	0.5	0
0.01	50	1	6	1	0.5	0

AFP trained on Trypanosomiasis dataset

Epochs	Dropout	Learning_rate	num_layers	num_timesteps	Batch_size	Balanced Accuracy	F1 -score
60	0.15	0.001	2	2	16	**0.81**	**0.67**
60	0.15	0.001	4	4	16	0.79	0.66
60	0.1	0.001	2	2	16	0.79	0.66
60	0.1	0.001	2	3	16	0.79	0.65
60	0.15	0.001	3	2	16	0.8	0.64
60	0.15	0.001	3	3	16	0.8	0.64
60	0.1	0.001	2	4	16	0.79	0.64
60	0.1	0.001	3	2	16	0.79	0.64
60	0.1	0.001	3	3	16	0.79	0.64
60	0.1	0.001	3	4	16	0.79	0.64
60	0.15	0.001	2	3	32	0.78	0.64
60	0.15	0.001	2	4	32	0.78	0.64
60	0.15	0.001	3	4	16	0.8	0.63
60	0.15	0.001	3	2	32	0.78	0.63
60	0.15	0.001	3	3	32	0.78	0.63
60	0.1	0.001	4	4	32	0.77	0.63
60	0.15	0.001	2	3	16	0.81	0.62
60	0.15	0.001	4	2	16	0.8	0.62
60	0.1	0.001	4	2	16	0.79	0.62
60	0.1	0.001	4	3	16	0.79	0.62
60	0.1	0.001	4	4	16	0.79	0.62
60	0.15	0.001	3	4	32	0.78	0.62
60	0.15	0.001	4	2	32	0.78	0.62
60	0.15	0.001	4	3	32	0.78	0.62
60	0.15	0.001	4	4	32	0.78	0.62
80	0.15	0.001	2	2	16	0.77	0.62
80	0.1	0.001	3	2	16	0.76	0.62
60	0.15	0.001	2	2	32	0.79	0.61
60	0.1	0.001	2	2	32	0.78	0.61
60	0.1	0.001	2	3	32	0.78	0.61
60	0.1	0.001	2	4	32	0.78	0.61
60	0.1	0.001	3	2	32	0.78	0.61
60	0.1	0.001	3	3	32	0.78	0.61
80	0.15	0.001	2	3	16	0.77	0.61
80	0.15	0.001	2	4	16	0.77	0.61
80	0.15	0.001	3	2	16	0.77	0.61
60	0.15	0.001	2	4	16	0.81	0.6
60	0.1	0.001	3	4	32	0.78	0.6
60	0.1	0.001	4	2	32	0.78	0.6
80	0.15	0.001	3	3	16	0.77	0.6
80	0.15	0.001	3	4	16	0.77	0.6
80	0.15	0.001	4	2	16	0.77	0.6
80	0.1	0.001	3	3	16	0.76	0.6
80	0.1	0.001	3	4	16	0.76	0.6
80	0.1	0.001	4	2	16	0.76	0.6
80	0.1	0.001	4	3	16	0.76	0.6
80	0.15	0.001	2	3	32	0.75	0.6
80	0.15	0.001	2	4	32	0.75	0.6
80	0.15	0.001	4	3	16	0.77	0.59
80	0.15	0.001	4	4	16	0.77	0.59
80	0.1	0.001	2	2	16	0.77	0.59
80	0.1	0.001	4	4	16	0.76	0.59
80	0.15	0.001	3	2	32	0.75	0.59
80	0.15	0.001	3	3	32	0.75	0.59
60	0.15	0.001	4	3	16	0.8	0.58
60	0.1	0.001	4	3	32	0.78	0.58
80	0.1	0.001	2	3	16	0.77	0.58
80	0.15	0.001	3	4	32	0.75	0.58
80	0.15	0.001	4	2	32	0.75	0.58
80	0.1	0.001	2	3	32	0.74	0.58
80	0.15	0.001	4	3	32	0.75	0.57
80	0.15	0.001	4	4	32	0.75	0.57
80	0.1	0.001	2	4	32	0.74	0.57
80	0.1	0.001	3	2	32	0.74	0.57
80	0.1	0.001	3	3	32	0.74	0.57
80	0.1	0.001	2	4	16	0.77	0.56
80	0.15	0.001	2	2	32	0.76	0.56
80	0.1	0.001	2	2	32	0.75	0.56
80	0.1	0.001	3	4	32	0.74	0.56
80	0.1	0.001	4	3	32	0.72	0.54
80	0.1	0.001	4	2	32	0.73	0.52
80	0.1	0.001	4	4	32	0.69	0.49

Cross validation :

AFP : Epoch = 60, Dropout = 0.15 Learning rate = 0.001, Batch size = 16, num_layers=2, num_timesteps= 2

K	Test Accuracy	Test ROC-AUC	Test Balanced_acc	Test MCC	Test Precision	Test Recall	Test F1 score	Test Cohen's Kappa
1	0.93	0.88	0.77	0.57	0.66	0.56	0.61	0.52
2	0.94	0.89	0.78	0.59	0.67	0.58	0.62	0.56
3	0.94	0.88	0.77	0.57	0.65	0.56	0.6	0.52
4	0.93	0.9	0.79	0.59	0.64	0.62	0.63	0.51
5	0.93	0.88	0.78	0.58	0.65	0.6	0.62	0.57
Mean	0.93	0.89	0.78	0.58	0.65	0.58	0.62	0.54
SD	0.0055	0.0089	0.008	0.01	0.01	0.03	0.01	0.03
IC	0.93 ±8.50E-05	0.89 ±0.00014	0.78 ±1.2E-4	0.58 ±1.5E-4	0.65 ±1.5E-4	0.58 ±4.6E-4	0.62 ±1.5E-4	0.54 ±4.6E-4

MLP trained on Trypanosomiasis dataset

_hidden_laye	Max_iteration	Activation	solver	alpha	Learning rate	Balanced Accuracy	F1 -score
(100, 50, 25)	50	tanh	adam	0.01	adaptive	**0.82**	**0.67**
(100, 50, 25)	50	relu	sgd	0.0001	constant	0.82	0.66
(100, 50, 25)	100	tanh	sgd	0.001	constant	0.81	0.66
(100, 50, 25)	100	tanh	sgd	0.001	adaptive	0.81	0.66
(100, 50, 25)	100	relu	adam	0.001	constant	0.8	0.66
(100, 50, 25)	100	relu	adam	0.001	adaptive	0.8	0.66
(100, 50, 25)	120	relu	adam	0.001	constant	0.79	0.66
(100, 50, 25)	50	tanh	sgd	0.0001	constant	0.84	0.65
(100, 50, 25)	50	relu	sgd	0.0001	adaptive	0.82	0.65
(100, 50, 25)	100	tanh	sgd	0.01	constant	0.81	0.65
(100, 50, 25)	100	tanh	sgd	0.01	adaptive	0.81	0.65
(100, 50, 25)	100	tanh	adam	0.0001	constant	0.81	0.65
(100, 50, 25)	100	tanh	adam	0.0001	adaptive	0.81	0.65
(100, 50, 25)	100	tanh	adam	0.001	constant	0.81	0.65
(100, 50, 25)	100	tanh	adam	0.001	adaptive	0.81	0.65
(100, 50, 25)	100	tanh	adam	0.01	constant	0.81	0.65
(100, 50, 25)	100	relu	adam	0.01	constant	0.8	0.65
(100, 50, 25)	120	relu	adam	0.001	adaptive	0.79	0.65
(150, 75, 25)	50	tanh	adam	0.0001	constant	0.77	0.65
(100, 50, 25)	50	tanh	sgd	0.001	adaptive	0.83	0.64
(100, 50, 25)	50	relu	sgd	0.001	constant	0.82	0.64
(100, 50, 25)	50	relu	sgd	0.001	adaptive	0.82	0.64
(100, 50, 25)	50	relu	sgd	0.01	constant	0.82	0.64
(100, 50, 25)	50	relu	sgd	0.01	adaptive	0.82	0.64
(100, 50, 25)	50	relu	adam	0.0001	constant	0.82	0.64
(100, 50, 25)	100	tanh	adam	0.01	adaptive	0.81	0.64
(100, 50, 25)	100	relu	sgd	0.0001	constant	0.81	0.64
(100, 50, 25)	100	relu	adam	0.01	adaptive	0.8	0.64
(100, 50, 25)	120	tanh	sgd	0.0001	constant	0.8	0.64
(100, 50, 25)	120	tanh	sgd	0.0001	adaptive	0.8	0.64
(100, 50, 25)	120	tanh	sgd	0.001	constant	0.8	0.64
(100, 50, 25)	120	tanh	sgd	0.001	adaptive	0.8	0.64
(100, 50, 25)	120	tanh	sgd	0.01	constant	0.8	0.64
(100, 50, 25)	120	tanh	sgd	0.01	adaptive	0.8	0.64
(100, 50, 25)	120	tanh	adam	0.0001	constant	0.8	0.64
(100, 50, 25)	120	relu	adam	0.01	constant	0.79	0.64
(100, 50, 25)	120	relu	adam	0.01	adaptive	0.79	0.64
(120, 80, 40)	50	tanh	sgd	0.0001	constant	0.79	0.64
(120, 80, 40)	50	tanh	sgd	0.0001	adaptive	0.79	0.64
(120, 80, 40)	100	relu	sgd	0.001	constant	0.78	0.64
(100, 50, 25)	50	tanh	sgd	0.01	constant	0.83	0.63
(100, 50, 25)	50	relu	adam	0.0001	adaptive	0.82	0.63
(100, 50, 25)	50	relu	adam	0.001	constant	0.82	0.63
(100, 50, 25)	100	relu	sgd	0.0001	adaptive	0.81	0.63
(100, 50, 25)	100	relu	sgd	0.001	constant	0.81	0.63
(100, 50, 25)	100	relu	sgd	0.001	adaptive	0.81	0.63
(100, 50, 25)	120	tanh	adam	0.0001	adaptive	0.8	0.63
(100, 50, 25)	120	tanh	adam	0.001	constant	0.8	0.63
(100, 50, 25)	120	tanh	adam	0.001	adaptive	0.8	0.63
(100, 50, 25)	120	tanh	adam	0.01	constant	0.8	0.63
(100, 50, 25)	120	tanh	adam	0.01	adaptive	0.8	0.63
(100, 50, 25)	120	relu	sgd	0.0001	constant	0.8	0.63
(100, 50, 25)	120	relu	sgd	0.0001	adaptive	0.8	0.63
(120, 80, 40)	50	tanh	sgd	0.001	constant	0.79	0.63
(120, 80, 40)	50	tanh	sgd	0.001	adaptive	0.79	0.63
(120, 80, 40)	50	tanh	sgd	0.01	constant	0.79	0.63
(120, 80, 40)	50	tanh	sgd	0.01	adaptive	0.79	0.63
(120, 80, 40)	50	tanh	adam	0.0001	constant	0.79	0.63
(120, 80, 40)	50	tanh	adam	0.0001	adaptive	0.79	0.63
(120, 80, 40)	50	tanh	adam	0.001	constant	0.79	0.63
(120, 80, 40)	50	tanh	adam	0.001	adaptive	0.79	0.63
(120, 80, 40)	50	tanh	adam	0.01	constant	0.79	0.63
(120, 80, 40)	100	relu	sgd	0.001	adaptive	0.78	0.63
(120, 80, 40)	100	relu	sgd	0.01	constant	0.78	0.63
(120, 80, 40)	100	relu	sgd	0.01	adaptive	0.78	0.63
(120, 80, 40)	100	relu	adam	0.0001	constant	0.78	0.63
(120, 80, 40)	100	relu	adam	0.0001	adaptive	0.78	0.63
(120, 80, 40)	100	relu	adam	0.001	constant	0.78	0.63
(150, 75, 25)	50	tanh	adam	0.0001	adaptive	0.77	0.63
(100, 50, 25)	50	tanh	sgd	0.0001	adaptive	0.84	0.62
(100, 50, 25)	50	tanh	sgd	0.01	adaptive	0.83	0.62
(100, 50, 25)	50	tanh	adam	0.0001	constant	0.83	0.62
(100, 50, 25)	50	relu	adam	0.001	adaptive	0.82	0.62
(100, 50, 25)	100	relu	sgd	0.01	constant	0.81	0.62
(100, 50, 25)	100	relu	sgd	0.01	adaptive	0.81	0.62
(100, 50, 25)	100	relu	adam	0.0001	constant	0.81	0.62
(100, 50, 25)	100	relu	adam	0.0001	adaptive	0.81	0.62
(100, 50, 25)	120	relu	sgd	0.001	constant	0.8	0.62
(100, 50, 25)	120	relu	sgd	0.001	adaptive	0.8	0.62
(100, 50, 25)	120	relu	sgd	0.01	constant	0.8	0.62
(100, 50, 25)	120	relu	sgd	0.01	adaptive	0.8	0.62
(120, 80, 40)	50	tanh	adam	0.01	adaptive	0.79	0.62
(120, 80, 40)	50	relu	sgd	0.0001	constant	0.79	0.62
(120, 80, 40)	50	relu	sgd	0.0001	adaptive	0.79	0.62
(120, 80, 40)	50	relu	sgd	0.001	constant	0.79	0.62
(120, 80, 40)	50	relu	sgd	0.001	adaptive	0.79	0.62
(120, 80, 40)	50	relu	sgd	0.01	constant	0.79	0.62
(120, 80, 40)	50	relu	sgd	0.01	adaptive	0.79	0.62
(120, 80, 40)	50	relu	adam	0.0001	constant	0.79	0.62
(120, 80, 40)	50	relu	adam	0.0001	adaptive	0.79	0.62
(120, 80, 40)	50	relu	adam	0.001	constant	0.79	0.62
(120, 80, 40)	50	relu	adam	0.001	adaptive	0.79	0.62
(120, 80, 40)	50	relu	adam	0.01	constant	0.79	0.62
(120, 80, 40)	50	relu	adam	0.01	adaptive	0.79	0.62
(120, 80, 40)	100	tanh	sgd	0.0001	constant	0.79	0.62
(120, 80, 40)	100	tanh	sgd	0.0001	adaptive	0.79	0.62
(120, 80, 40)	100	relu	adam	0.001	adaptive	0.78	0.62
(120, 80, 40)	100	relu	adam	0.01	constant	0.78	0.62
(120, 80, 40)	100	relu	adam	0.01	adaptive	0.78	0.62
(120, 80, 40)	120	tanh	sgd	0.0001	constant	0.78	0.62
(120, 80, 40)	120	tanh	sgd	0.0001	adaptive	0.78	0.62
(120, 80, 40)	120	tanh	sgd	0.001	constant	0.78	0.62
(120, 80, 40)	120	tanh	sgd	0.001	adaptive	0.78	0.62
(120, 80, 40)	120	tanh	sgd	0.01	constant	0.78	0.62
(120, 80, 40)	120	tanh	sgd	0.01	adaptive	0.78	0.62
(120, 80, 40)	120	tanh	adam	0.0001	constant	0.78	0.62
(120, 80, 40)	120	tanh	adam	0.0001	adaptive	0.78	0.62
(120, 80, 40)	120	tanh	adam	0.001	constant	0.78	0.62
(120, 80, 40)	120	tanh	adam	0.001	adaptive	0.78	0.62
(120, 80, 40)	120	tanh	adam	0.01	constant	0.78	0.62
(120, 80, 40)	120	tanh	adam	0.01	adaptive	0.78	0.62
(150, 75, 25)	50	tanh	adam	0.001	constant	0.77	0.62
(150, 75, 25)	50	tanh	adam	0.001	adaptive	0.77	0.62
(150, 75, 25)	50	tanh	adam	0.01	constant	0.77	0.62
(150, 75, 25)	50	tanh	adam	0.01	adaptive	0.77	0.62
(150, 75, 25)	50	relu	sgd	0.0001	constant	0.77	0.62
(150, 75, 25)	100	tanh	adam	0.001	adaptive	0.76	0.62
(150, 75, 25)	100	tanh	adam	0.01	constant	0.76	0.62
(100, 50, 25)	50	relu	adam	0.01	constant	0.82	0.61
(100, 50, 25)	120	relu	adam	0.0001	constant	0.8	0.61
(100, 50, 25)	120	relu	adam	0.0001	adaptive	0.8	0.61
(120, 80, 40)	100	tanh	sgd	0.001	constant	0.79	0.61
(120, 80, 40)	100	tanh	sgd	0.001	adaptive	0.79	0.61
(120, 80, 40)	100	tanh	sgd	0.01	constant	0.79	0.61
(120, 80, 40)	100	tanh	sgd	0.01	adaptive	0.79	0.61
(120, 80, 40)	100	tanh	adam	0.0001	constant	0.79	0.61
(120, 80, 40)	100	tanh	adam	0.0001	adaptive	0.79	0.61
(120, 80, 40)	100	tanh	adam	0.001	constant	0.79	0.61
(120, 80, 40)	100	tanh	adam	0.001	adaptive	0.79	0.61
(120, 80, 40)	120	relu	sgd	0.0001	constant	0.78	0.61
(120, 80, 40)	120	relu	sgd	0.0001	adaptive	0.78	0.61
(120, 80, 40)	120	relu	sgd	0.001	constant	0.78	0.61
(120, 80, 40)	120	relu	sgd	0.001	adaptive	0.78	0.61
(120, 80, 40)	120	relu	sgd	0.01	constant	0.78	0.61
(120, 80, 40)	120	relu	sgd	0.01	adaptive	0.78	0.61
(120, 80, 40)	120	relu	adam	0.0001	constant	0.78	0.61
(120, 80, 40)	120	relu	adam	0.0001	adaptive	0.78	0.61
(120, 80, 40)	120	relu	adam	0.001	constant	0.78	0.61
(120, 80, 40)	120	relu	adam	0.001	adaptive	0.78	0.61
(150, 75, 25)	50	relu	sgd	0.0001	adaptive	0.77	0.61
(150, 75, 25)	50	relu	sgd	0.001	constant	0.77	0.61
(150, 75, 25)	50	relu	sgd	0.001	adaptive	0.77	0.61

Cross validation :

MLP : hidden_layer_sizes =(100, 50, 25) , max_iterations= 50, activation = tanh, solver = adam, alpha = 0.01, learning_rate = adaptive

K	Test Accuracy	Test ROC-AUC	Test Balanced_acc	Test MCC	Test Precision	Test Recall	Test F1 score	Test Cohen's Kappa
1	0.94	0.86	0.77	0.59	0.68	0.55	0.61	0.59
2	0.95	0.87	0.72	0.55	0.73	0.47	0.57	0.54
3	0.94	0.88	0.77	0.57	0.65	0.56	0.6	0.53
4	0.94	0.87	0.76	0.54	0.6	0.55	0.58	0.54
5	0.94	0.87	0.76	0.55	0.65	0.54	0.59	0.56
Mean	0.94	0.87	0.76	0.56	0.66	0.53	0.59	0.55
SD	0.004	0.007	0.021	0.02	0.05	0.03	0.016	0.02
IC	0.94 ±6.18E-05	0.87 ±0.0001	0.76 ±3.2E-4	0.56 ±3.0E-4	0.66 ±7.7E-4	0.53 ±4.6E-4	0.59 ±2.4E-4	0.55 ±3.0E-4

(150, 75, 25)	50	relu	sgd	0.01	constant	0.77	0.61
(150, 75, 25)	50	relu	sgd	0.01	adaptive	0.77	0.61
(150, 75, 25)	100	tanh	adam	0.01	adaptive	0.76	0.61
(150, 75, 25)	100	relu	sgd	0.0001	constant	0.76	0.61
(150, 75, 25)	100	relu	sgd	0.0001	adaptive	0.76	0.61
(150, 75, 25)	100	relu	sgd	0.001	constant	0.76	0.61
(100, 50, 25)	50	tanh	sgd	0.001	constant	0.84	0.6
(100, 50, 25)	50	tanh	adam	0.0001	adaptive	0.83	0.6
(100, 50, 25)	50	tanh	adam	0.001	constant	0.83	0.6
(100, 50, 25)	50	relu	adam	0.01	adaptive	0.82	0.6
(120, 80, 40)	100	tanh	adam	0.01	constant	0.79	0.6
(120, 80, 40)	100	tanh	adam	0.01	adaptive	0.79	0.6
(120, 80, 40)	100	relu	sgd	0.0001	constant	0.79	0.6
(120, 80, 40)	100	relu	sgd	0.0001	adaptive	0.79	0.6
(120, 80, 40)	120	relu	adam	0.01	constant	0.78	0.6
(120, 80, 40)	120	relu	adam	0.01	adaptive	0.78	0.6
(150, 75, 25)	50	tanh	sgd	0.0001	constant	0.78	0.6
(150, 75, 25)	50	tanh	sgd	0.0001	adaptive	0.78	0.6
(150, 75, 25)	50	tanh	sgd	0.001	constant	0.78	0.6
(150, 75, 25)	50	tanh	sgd	0.001	adaptive	0.78	0.6
(150, 75, 25)	50	tanh	sgd	0.01	constant	0.78	0.6
(150, 75, 25)	50	relu	adam	0.0001	constant	0.77	0.6
(150, 75, 25)	50	relu	adam	0.0001	adaptive	0.77	0.6
(150, 75, 25)	50	relu	adam	0.001	constant	0.77	0.6
(150, 75, 25)	50	relu	adam	0.001	adaptive	0.77	0.6
(150, 75, 25)	50	relu	adam	0.01	constant	0.77	0.6
(150, 75, 25)	50	relu	adam	0.01	adaptive	0.77	0.6
(150, 75, 25)	100	tanh	sgd	0.0001	constant	0.77	0.6
(150, 75, 25)	100	tanh	sgd	0.0001	adaptive	0.77	0.6
(150, 75, 25)	100	tanh	sgd	0.001	constant	0.77	0.6
(150, 75, 25)	100	tanh	sgd	0.001	adaptive	0.77	0.6
(150, 75, 25)	100	relu	sgd	0.001	adaptive	0.76	0.6
(150, 75, 25)	100	relu	sgd	0.01	constant	0.76	0.6
(150, 75, 25)	100	relu	sgd	0.01	adaptive	0.76	0.6
(150, 75, 25)	100	relu	adam	0.0001	constant	0.76	0.6
(150, 75, 25)	100	relu	adam	0.0001	adaptive	0.76	0.6
(150, 75, 25)	100	relu	adam	0.01	constant	0.75	0.6
(150, 75, 25)	100	relu	adam	0.01	adaptive	0.75	0.6
(150, 75, 25)	120	tanh	sgd	0.0001	constant	0.75	0.6
(150, 75, 25)	120	tanh	sgd	0.0001	adaptive	0.75	0.6
(150, 75, 25)	120	tanh	adam	0.001	constant	0.74	0.6
(150, 75, 25)	50	tanh	sgd	0.01	adaptive	0.78	0.59
(150, 75, 25)	100	tanh	sgd	0.01	constant	0.77	0.59
(150, 75, 25)	100	tanh	sgd	0.01	adaptive	0.77	0.59
(150, 75, 25)	100	tanh	adam	0.0001	constant	0.77	0.59
(150, 75, 25)	100	tanh	adam	0.0001	adaptive	0.77	0.59
(150, 75, 25)	100	relu	adam	0.001	constant	0.76	0.59
(150, 75, 25)	120	tanh	sgd	0.001	constant	0.75	0.59
(150, 75, 25)	120	tanh	sgd	0.001	adaptive	0.75	0.59
(150, 75, 25)	120	tanh	sgd	0.01	constant	0.75	0.59
(150, 75, 25)	120	tanh	sgd	0.01	adaptive	0.75	0.59
(150, 75, 25)	120	tanh	adam	0.0001	constant	0.75	0.59
(150, 75, 25)	120	tanh	adam	0.001	adaptive	0.74	0.59
(150, 75, 25)	120	tanh	adam	0.01	constant	0.74	0.59
(100, 50, 25)	50	tanh	adam	0.001	adaptive	0.83	0.58
(100, 50, 25)	100	tanh	sgd	0.0001	constant	0.82	0.58
(100, 50, 25)	100	tanh	sgd	0.0001	adaptive	0.82	0.58
(150, 75, 25)	100	tanh	adam	0.001	constant	0.77	0.58
(150, 75, 25)	100	relu	adam	0.001	adaptive	0.76	0.58
(150, 75, 25)	120	tanh	adam	0.0001	adaptive	0.75	0.58
(150, 75, 25)	120	tanh	adam	0.01	adaptive	0.74	0.58
(150, 75, 25)	120	relu	sgd	0.0001	constant	0.74	0.58
(150, 75, 25)	120	relu	sgd	0.0001	adaptive	0.74	0.58
(150, 75, 25)	120	relu	sgd	0.001	constant	0.74	0.58
(150, 75, 25)	120	relu	sgd	0.001	adaptive	0.74	0.58
(150, 75, 25)	120	relu	sgd	0.01	constant	0.74	0.58
(100, 50, 25)	50	tanh	adam	0.01	constant	0.83	0.57
(150, 75, 25)	120	relu	sgd	0.01	adaptive	0.74	0.57
(150, 75, 25)	120	relu	adam	0.0001	constant	0.73	0.57
(150, 75, 25)	120	relu	adam	0.0001	adaptive	0.72	0.56
(150, 75, 25)	120	relu	adam	0.001	constant	0.72	0.55
(150, 75, 25)	120	relu	adam	0.001	adaptive	0.71	0.55
(150, 75, 25)	120	relu	adam	0.01	constant	0.71	0.54
(150, 75, 25)	120	relu	adam	0.01	adaptive	0.69	0.53

KNN trained on Trypanosomiasis dataset

n_neighbors	weights	leaf_size	Balanced Accuracy	F1 -score
3	distance	30	**0.79**	**0.64**
3	distance	40	0.79	0.64
3	distance	50	0.79	0.64
3	distance	60	0.79	0.64
5	distance	30	0.77	0.63
5	distance	40	0.77	0.63
5	distance	50	0.77	0.63
5	distance	60	0.77	0.63
3	uniform	30	0.77	0.62
3	uniform	40	0.77	0.62
3	uniform	50	0.77	0.62
3	uniform	60	0.77	0.62
5	uniform	30	0.76	0.6
5	uniform	40	0.76	0.6
5	uniform	50	0.76	0.6
5	uniform	60	0.76	0.6
7	distance	30	0.75	0.59
7	distance	40	0.75	0.59
7	distance	50	0.75	0.59
7	distance	60	0.75	0.59
10	distance	30	0.73	0.58
10	distance	40	0.73	0.58
10	distance	50	0.73	0.58
10	distance	60	0.73	0.58
7	uniform	30	0.72	0.55
7	uniform	40	0.72	0.55
7	uniform	50	0.72	0.55
7	uniform	60	0.72	0.55
10	uniform	30	0.7	0.53
10	uniform	40	0.7	0.53
10	uniform	50	0.7	0.53
10	uniform	60	0.7	0.53

Cross validation :

KNN : n_neighbors = 3, weights= distance leaf_size=30

K	Test Accuracy	Test ROC-AUC	Test Balanced_acc	Test MCC	Test Precision	Test Recall	Test F1 score	Test Cohen's Kappa
1	0.94	0.83	0.75	0.57	0.68	0.53	0.59	0.56
2	0.94	0.87	0.75	0.57	0.68	0.53	0.6	0.54
3	0.94	0.82	0.76	0.54	0.6	0.54	0.57	0.53
4	0.94	0.83	0.75	0.56	0.68	0.51	0.58	0.55
5	0.95	0.84	0.74	0.57	0.71	0.5	0.59	0.56
Mean	0.94	0.84	0.75	0.56	0.67	0.52	0.59	0.55
SD	0.004	0.019	0.007	0.013	0.041	0.016	0.011	0.013
IC	0.94 ±6.18E-05	0.84 ±0.0003	0.75 ±1.0E-4	0.56 ±2.0E-4	0.67 ±6.3E-4	0.52 ±2.4E-4	0.59 ±1.7E-4	0.55 ±2.0E-4

AFP trained on COVID dataset

Epochs	Dropout	Learning_rate	num_layers	num_timesteps	Batch_size	Balanced Accuracy	F1 -score
80	0.1	0.001	3	2	32	**0.72**	**0.51**
60	0.1	0.001	3	4	16	0.72	0.51
60	0.1	0.001	4	2	32	0.71	0.51
60	0.1	0.001	4	2	16	0.7	0.5
80	0.15	0.001	4	2	32	0.71	0.49
60	0.15	0.001	2	3	16	0.71	0.49
60	0.1	0.001	4	4	16	0.7	0.49
80	0.15	0.001	2	2	16	0.7	0.49
80	0.15	0.001	3	2	16	0.7	0.49
80	0.15	0.001	4	2	16	0.7	0.49
80	0.15	0.001	4	4	16	0.7	0.49
80	0.1	0.001	2	4	16	0.7	0.49
80	0.1	0.001	2	2	16	0.7	0.49
60	0.15	0.001	3	3	16	0.69	0.49
80	0.1	0.001	2	2	16	0.69	0.49
80	0.1	0.001	4	4	16	0.69	0.49
80	0.15	0.001	4	4	32	0.71	0.48
80	0.1	0.001	4	3	16	0.7	0.48
60	0.1	0.001	3	4	32	0.71	0.47
80	0.15	0.001	4	3	32	0.71	0.47
60	0.1	0.001	2	4	32	0.7	0.47
80	0.15	0.001	2	3	32	0.7	0.47
80	0.15	0.001	2	4	32	0.7	0.47
80	0.15	0.001	3	3	32	0.7	0.47
80	0.1	0.001	3	4	32	0.7	0.47
60	0.15	0.001	4	2	16	0.7	0.47
60	0.15	0.001	4	3	16	0.69	0.47
60	0.15	0.001	2	2	32	0.69	0.46
60	0.15	0.001	3	3	32	0.69	0.46
60	0.1	0.001	2	2	32	0.69	0.46
80	0.1	0.001	4	4	32	0.69	0.46
60	0.1	0.001	2	3	16	0.69	0.46
80	0.15	0.001	3	4	16	0.69	0.46
80	0.15	0.001	4	3	16	0.69	0.46
60	0.15	0.001	3	2	32	0.68	0.46
60	0.15	0.001	2	2	16	0.68	0.46
60	0.1	0.001	2	2	16	0.68	0.46
60	0.1	0.001	3	3	16	0.68	0.46
80	0.15	0.001	2	3	16	0.68	0.46
60	0.15	0.001	2	4	32	0.69	0.45
60	0.15	0.001	3	4	32	0.69	0.45
80	0.15	0.001	3	4	32	0.69	0.45
80	0.1	0.001	4	3	32	0.69	0.45
60	0.1	0.001	3	3	32	0.68	0.45
80	0.15	0.001	3	2	32	0.68	0.45
60	0.15	0.001	2	4	16	0.68	0.45
60	0.15	0.001	3	4	16	0.68	0.45
60	0.1	0.001	3	2	16	0.68	0.45
80	0.1	0.001	4	2	16	0.68	0.45
60	0.1	0.001	4	3	16	0.67	0.45
80	0.1	0.001	2	3	32	0.69	0.44
80	0.1	0.001	3	3	32	0.69	0.44
60	0.15	0.001	4	2	32	0.68	0.44
80	0.1	0.001	2	4	32	0.68	0.44
80	0.1	0.001	3	2	32	0.68	0.44
80	0.1	0.001	4	2	32	0.68	0.44
80	0.1	0.001	2	3	16	0.67	0.44
60	0.1	0.001	3	2	32	0.7	0.43
60	0.1	0.001	2	3	32	0.67	0.43
80	0.15	0.001	2	2	32	0.67	0.43
80	0.15	0.001	2	4	16	0.67	0.43
80	0.15	0.001	3	3	16	0.67	0.43
60	0.1	0.001	2	4	16	0.66	0.43
60	0.15	0.001	4	4	32	0.68	0.42
60	0.15	0.001	2	3	32	0.67	0.42
60	0.15	0.001	4	3	32	0.66	0.42
60	0.15	0.001	3	2	16	0.66	0.42
60	0.1	0.001	4	3	32	0.69	0.41
80	0.1	0.001	3	3	16	0.67	0.41
80	0.1	0.001	3	4	16	0.66	0.41
60	0.15	0.001	4	4	16	0.65	0.4
60	0.1	0.001	4	4	32	0.67	0.38

Cross validation :

AFP : Epoch = 80, Dropout = 0.1 Learning rate = 0.001, Batch size = 32, num_layers=2, num_timesteps= 3

K	Test Accuracy	Test ROC-AUC	Test Balanced_acc	Test MCC	Test Precision	Test Recall	Test F1 score	Test Cohen's Kappa
1	0.93	0.81	0.73	0.54	0.66	0.49	0.56	0.54
2	0.93	0.8	0.74	0.52	0.62	0.5	0.55	0.49
3	0.93	0.78	0.71	0.49	0.61	0.45	0.52	0.48
4	0.94	0.81	0.73	0.53	0.65	0.49	0.56	0.45
5	0.93	0.82	0.71	0.46	0.55	0.47	0.51	0.44
Mean	0.93	0.80	0.72	0.51	0.62	0.48	0.54	0.48
SD	0.004	0.015	0.013	0.033	0.043	0.02	0.023	0.04
IC	0.93 ±7.13E-05	0.80 ±2.6E-4	0.72 ±2.3E-4	0.51 ±5.8E-4	0.62 ±7.6E-4	0.48 ±3.5E-4	0.54 ±4.1E-4	0.48 ±7.1E-4

MolFormer trained on COVID dataset

Epochs	Batch_size	warmup_steps	Learning_rate	weight_decay	Balanced Accuracy	F1 -score
3	16	500	0.00005	0.001	**0.92**	**0.65**
3	16	500	0.00005	0.01	0.91	0.61
5	16	500	0.00005	0.01	0.91	0.60
5	16	500	0.00005	0.001	0.90	0.59
3	32	1000	0.0005	0.001	0.88	0.53
3	16	500	0.0005	0.01	0.87	0.5
3	16	500	0.0005	0.001	0.87	0.5
3	16	1000	0.00005	0.01	0.87	0.5
3	16	1000	0.00005	0.001	0.87	0.5
3	16	1000	0.0005	0.01	0.87	0.5
3	16	1000	0.0005	0.001	0.87	0.5
3	32	500	0.00005	0.01	0.87	0.5
3	32	500	0.00005	0.001	0.87	0.5
3	32	500	0.0005	0.01	0.87	0.5
3	32	500	0.0005	0.001	0.87	0.5
3	32	1000	0.00005	0.01	0.87	0.5
3	32	1000	0.00005	0.001	0.87	0.5
3	32	1000	0.0005	0.01	0.87	0.5
5	16	500	0.0005	0.01	0.87	0.5
5	16	500	0.0005	0.001	0.87	0.5
5	16	1000	0.00005	0.01	0.87	0.5
5	16	1000	0.00005	0.001	0.87	0.5
5	16	1000	0.0005	0.01	0.87	0.5
5	16	1000	0.0005	0.001	0.87	0.5
5	32	500	0.00005	0.01	0.87	0.5
5	32	500	0.00005	0.001	0.87	0.5
5	32	500	0.0005	0.01	0.87	0.5
5	32	500	0.0005	0.001	0.87	0.5
5	32	1000	0.00005	0.01	0.87	0.5
5	32	1000	0.00005	0.001	0.87	0.5
5	32	1000	0.0005	0.01	0.87	0.5
5	32	1000	0.0005	0.001	0.87	0.5

Cross validation :

MolFormer : Epoch = 3, Batch size = 16, warmup_steps = 500, Learning rate = 0.00005, weight_decay = 0.010

K	Test Accuracy	Test ROC-AUC	Test Balanced_acc	Test MCC	Test Precision	Test Recall	Test F1 score	Test Cohen's Kappa
1	0.94	0.75	0.91	0.6	0.76	0.52	0.62	0.63
2	0.93	0.78	0.92	0.6	0.7	0.58	0.63	0.53
3	0.93	0.75	0.9	0.56	0.69	0.52	0.59	0.60
4	0.94	0.74	0.91	0.57	0.74	0.49	0.59	0.61
5	0.93	0.71	0.89	0.54	0.76	0.43	0.55	0.59
Mean	0.93	0.75	0.91	0.57	0.73	0.51	0.60	0.59
SD	0.005	0.025	0.01	0.03	0.03	0.05	0.031	0.04
IC	0.93 ±8.92E-05	0.75 ±4.0E-4	0.91 ±1.7E-4	0.57 ±5.0E-4	0.73 ±5.0E-4	0.51 ±8.0E-4	0.60 ±5.0E-4	0.59 ±7.0E-4

GCN trained on COVID dataset

Epochs	Dropout	Batch_size	Graph_layers	param_pr_hidden_feat	Balanced Accuracy	F1 -score
60	0.15	64	[512, 128, 64]	128	**0.77**	**0.64**
60	0.15	64	[512, 128, 64]	256	0.77	0.61
60	0.15	64	[512, 128]	128	0.77	0.61
60	0.15	64	[512, 128]	256	0.77	0.6
60	0.15	64	[64, 64]	128	0.76	0.6
60	0.15	64	[64, 64]	256	0.76	0.59
60	0.1	64	[512, 128, 64]	128	0.74	0.59
60	0.1	64	[512, 128, 64]	256	0.73	0.59
60	0.1	64	[512, 128]	128	0.75	0.58
60	0.1	64	[512, 128]	256	0.77	0.57
60	0.1	64	[64, 64]	128	0.76	0.57
60	0.1	64	[64, 64]	256	0.74	0.57
60	0.15	32	[512, 128, 64]	128	0.73	0.57
60	0.15	32	[512, 128, 64]	256	0.77	0.56
60	0.15	32	[512, 128]	128	0.76	0.56
60	0.15	32	[512, 128]	256	0.75	0.56
60	0.15	32	[64, 64]	128	0.74	0.56
60	0.15	32	[64, 64]	256	0.74	0.56
60	0.1	32	[512, 128, 64]	128	0.74	0.56
60	0.1	32	[512, 128, 64]	256	0.74	0.56
60	0.1	32	[512, 128]	128	0.77	0.55
60	0.1	32	[512, 128]	256	0.76	0.55
60	0.1	32	[64, 64]	128	0.76	0.55
60	0.1	32	[64, 64]	256	0.77	0.54
80	0.15	64	[512, 128, 64]	128	0.7	0.54
80	0.15	64	[512, 128, 64]	256	0.78	0.53
80	0.15	64	[512, 128]	128	0.76	0.53
80	0.15	64	[512, 128]	256	0.74	0.53
80	0.15	64	[64, 64]	128	0.74	0.52
80	0.15	64	[64, 64]	256	0.69	0.52
80	0.1	64	[512, 128, 64]	128	0.76	0.51
80	0.1	64	[512, 128, 64]	256	0.7	0.51
80	0.1	64	[512, 128]	128	0.77	0.5
80	0.1	64	[512, 128]	256	0.76	0.5
80	0.1	64	[64, 64]	128	0.76	0.5
80	0.1	64	[64, 64]	256	0.69	0.5
80	0.15	32	[512, 128, 64]	128	0.78	0.48
80	0.15	32	[512, 128, 64]	256	0.78	0.48
80	0.15	32	[512, 128]	128	0.78	0.48
80	0.15	32	[512, 128]	256	0.74	0.48
80	0.15	32	[64, 64]	128	0.74	0.48
80	0.15	32	[64, 64]	256	0.76	0.47
80	0.1	32	[512, 128, 64]	128	0.66	0.46
80	0.1	32	[512, 128, 64]	256	0.77	0.44
80	0.1	32	[512, 128]	128	0.77	0.44
80	0.1	32	[512, 128]	256	0.63	0.4
80	0.1	32	[64, 64]	128	0.62	0.37
80	0.1	32	[64, 64]	256	0.55	0.17

Cross validation :

GCN : Epoch = 60, Dropout = 0.15 , Batch size = 64, Graph layers = [512, 128,64], param_hidden_feat = 128

K	Test Accuracy	Test ROC-AUC	Test Balanced_acc	Test MCC	Test Precision	Test Recall	Test F1 score	Test Cohen's Kappa
1	0.94	0.87	0.74	0.55	0.68	0.49	0.57	0.37
2	0.95	0.9	0.75	0.61	0.79	0.51	0.62	0.35
3	0.89	0.86	0.79	0.46	0.41	0.66	0.51	0.5
4	0.94	0.91	0.77	0.59	0.68	0.57	0.62	0.53
5	0.88	0.93	0.86	0.53	0.4	0.83	0.54	0.47
Mean	0.92	0.89	0.78	0.55	0.59	0.61	0.57	0.44
SD	0.032	0.029	0.05	0.06	0.2	0.14	0.05	0.08
IC	0.92 ±5.0E-4	0.89 ±5.0E-4	0.78 ±8.0E-4	0.55 ± ±1.0E-4	0.59 ±3.5E-4	0.61 ± ±2.5E-4	0.57 ±8.0E-4	0.44 ±1.4E-4

MPNN trained on COVID dataset

Epochs	Dropout	Learning_rate	num_layer_settoset	num_step_set2set	num_step_message_passing	Batch_size	Balanced Accuracy	F1 -score
60	0.15	0.001	3	6	3	16	**0.68**	**0.47**
60	0.15	0.001	3	6	3	32	0.68	0.47
60	0.1	0.001	2	4	2	32	0.69	0.46
80	0.1	0.001	3	6	2	16	0.68	0.45
80	0.15	0.001	3	4	3	32	0.68	0.45
60	0.15	0.001	3	4	2	16	0.68	0.45
60	0.15	0.001	2	6	3	16	0.67	0.45
60	0.15	0.001	3	4	2	32	0.69	0.44
60	0.1	0.001	2	4	3	32	0.69	0.44
60	0.1	0.001	3	4	3	32	0.67	0.44
80	0.15	0.001	3	4	2	32	0.69	0.43
80	0.15	0.001	2	4	2	16	0.68	0.43
80	0.1	0.001	3	4	3	32	0.68	0.43
60	0.15	0.001	2	4	2	16	0.68	0.43
80	0.15	0.001	3	4	2	16	0.67	0.43
60	0.15	0.001	2	4	2	32	0.67	0.43
80	0.1	0.001	3	4	2	32	0.69	0.42
60	0.1	0.001	2	4	3	16	0.68	0.42
80	0.15	0.001	2	4	3	16	0.67	0.42
80	0.1	0.001	3	4	2	16	0.67	0.42
60	0.15	0.001	2	6	2	32	0.67	0.42
80	0.15	0.001	3	6	3	16	0.66	0.41
80	0.15	0.001	2	6	3	32	0.66	0.41
80	0.1	0.001	3	6	2	32	0.66	0.41
60	0.15	0.001	2	4	3	32	0.66	0.41
60	0.15	0.001	2	6	3	32	0.65	0.41
60	0.15	0.001	3	4	3	32	0.65	0.41
60	0.15	0.001	3	4	3	16	0.68	0.4
60	0.1	0.001	3	4	2	16	0.66	0.4
60	0.1	0.001	3	6	3	16	0.66	0.4
80	0.1	0.001	2	6	2	16	0.65	0.4
80	0.1	0.001	3	6	3	16	0.65	0.4
60	0.1	0.001	3	4	2	32	0.65	0.4
80	0.15	0.001	2	6	2	16	0.64	0.4
80	0.15	0.001	2	4	2	32	0.67	0.39
60	0.1	0.001	3	4	3	16	0.65	0.39
80	0.1	0.001	2	6	3	16	0.64	0.39
80	0.1	0.001	3	4	3	16	0.64	0.39
80	0.1	0.001	2	6	2	32	0.66	0.38
80	0.1	0.001	2	4	2	16	0.65	0.38
80	0.1	0.001	2	4	3	16	0.65	0.38
80	0.15	0.001	2	4	3	32	0.64	0.38
80	0.1	0.001	2	4	3	32	0.64	0.38
60	0.1	0.001	2	6	3	32	0.64	0.38
60	0.1	0.001	3	6	2	32	0.63	0.38
80	0.15	0.001	2	6	3	16	0.65	0.37
80	0.15	0.001	3	6	2	16	0.63	0.37
80	0.1	0.001	2	6	3	32	0.65	0.36
80	0.15	0.001	3	4	3	16	0.63	0.36
80	0.15	0.001	2	6	2	32	0.62	0.35
60	0.1	0.001	2	6	2	16	0.62	0.35
80	0.15	0.001	3	6	2	32	0.63	0.34
60	0.15	0.001	2	6	2	16	0.62	0.34
60	0.1	0.001	3	6	3	32	0.61	0.34
60	0.1	0.001	2	6	3	16	0.61	0.33
60	0.15	0.001	3	6	2	32	0.61	0.33
80	0.1	0.001	2	4	2	32	0.62	0.32
60	0.15	0.001	3	6	2	16	0.61	0.32
60	0.1	0.001	3	6	2	16	0.6	0.29
80	0.15	0.001	3	6	3	32	0.59	0.29
80	0.1	0.001	3	6	3	32	0.59	0.28
60	0.15	0.001	2	4	3	16	0.59	0.28
60	0.1	0.001	2	4	2	16	0.58	0.26
60	0.1	0.001	2	6	2	32	0.56	0.21

Cross validation :

MPNN : Epoch = 60, Dropout = 0.15 , Batch size = 16, num_layer_set2set = 3, num_step_set2set=6,num_step_message_passing=3

K	Test Accuracy	Test ROC-AUC	Test Balanced_acc	Test MCC	Test Precision	Test Recall	Test F1 score	Test Cohen's Kappa
1	0.93	0.9	0.72	0.62	0.92	0.42	0.56	0.36
2	0.93	0.81	0.69	0.49	0.68	0.4	0.5	0.36
3	0.93	0.79	0.67	0.47	0.7	0.36	0.47	0.44
4	0.94	0.79	0.67	0.56	0.87	0.37	0.51	0.42
5	0.93	0.87	0.56	0.56	0.8	0.43	0.56	0.41
Mean	0.93	0.83	0.66	0.54	0.79	0.4	0.52	0.4
SD	0.004	0.05	0.061	0.06	0.10	0.03	0.04	0.04
IC	0.93 ±7.13E-05	0.83 ±8.0E-4	0.66 ±0.001	0.54 ±0.001	0.79 ±1.8E-3	0.40 ±5.0E-4	0.52 ±6.0E-4	0.40 ±6.0E-4

Supplementary Table S3 : External validation of optimized models' performances in comparison with experimental results from confirmatory bioassays

**Disease**	**Data State**	**Model**	**All molecules: no confidence threshold**				**Subsection of molecules above the 80% confidence threshold**			
			**TP**	**TN**	**FP**	**FN**	**TP**	**TN**	**FP**	**FN**
**HIV**	**Original**	XGBoost	1	11	0	43	0	11	0	40
		MLP	4	11	0	40	4	11	0	38
		RF	2	11	0	42	0	10	0	38
		KNN	1	11	0	43	0	11	0	40
	**RUS**	RF	23	10	1	21	6	0	1	1
		GCN	17	10	1	27	1	2	0	1
		AFP	33	2	9	11	21	1	4	8
		MPNN	0	11	0	44	0	11	0	44
	**1:10**	XGBoost	7	10	1	37	4	9	1	33
		KNN	8	11	0	36	8	5	0	25
		AFP	9	10	1	35	7	10	0	30
		MLP	9	11	0	35	5	8	0	30
**Malaria**	**Original**	MolFormer	9	15	25	14	-	-	-	-
		MLP	12	38	2	11	12	37	2	11
		ChemBERTa	16	38	2	7	16	38	1	6
		XGBoost	12	38	2	11	6	38	1	9
	**RUS**	GCN	22	32	8	1	21	22	2	1
		XGBoost	21	18	22	2	21	8	14	1
		AFP	22	33	7	1	22	32	6	1
		RF	19	16	24	4	3	1	0	0
	**1:10**	ChemBERTa	22	38	2	1	21	36	2	1
		AFP	22	39	1	1	22	38	1	1
		XGBoost	14	38	2	9	8	34	1	3
		MLP	18	35	5	5	16	33	2	3

**Trypanoso-miasis**	**Original**	XGBoost	0	137	0	112	0	105	0	117
		MLP	74	80	32	63	67	75	29	53
		RF	37	100	12	100	12	79	1	50
		KNN	49	95	17	88	22	80	5	61
	**RUS**	MLP	128	17	95	9	128	14	89	7
		RF	126	27	85	11	78	0	33	0
		MPNN	128	8	104	9	123	5	92	4
		GCN	119	23	89	18	97	3	50	3
	**1:10**	XGBoost	91	69	43	46	69	56	28	34
		AFP	95	50	62	42	87	42	55	38
		MLP	93	69	43	44	52	49	21	25
		KNN	94	62	50	43	53	37	25	18
**COVID-19**	**Original**	MolFormer	44	2	55	0	-	-	-	-
		GCN	14	46	11	30	3	39	3	17
		MLP	37	47	10	7	35	46	7	6
		RF	38	52	5	6	14	45	0	5
	**RUS**	RF	41	20	37	3	33	0	7	0
		AFP	34	30	27	10	30	23	22	8
		GCN	38	18	39	6	21	3	22	2
		MLP	43	15	42	1	42	13	36	1
	**1:10**	AFP	39	34	23	5	37	34	17	4
		MolFormer	30	32	25	14	-	-	-	-
		GCN	25	37	20	19	11	19	13	6
		MPNN	17	46	11	27	11	35	5	20

Supplementary Table S4 : Metadata and Descriptive Information on the used bioassays

AID	Disease	Target	Assay Type	Assay Protocol	Size
AID651610	HIV	HIV Env-mediated cell fusion	Primary cell based assay HIV entry inhibition via Env-mediated fusion (reporter-gene assay)	Cell-based fusion assay	354,383
AID1822	Malaria	PFM18AAP	Primary biochemical (enzyme activity assay)	QFRET-based biochemical HTS	290,893
AID624268	Trypanosomiasis	T.Brucei MetRS	Primary biochemical (enzyme activity assay)	Luminescence-based biochemical HTS	364,131
AID1706	COVID-19	3CLPro	Primary biochemical (enzyme activity assay)	QFRET-based biochemical HTS	290,893
